# A Systematic Review of Biomarkers and Risk of Incident Type 2 Diabetes: An Overview of Epidemiological, Prediction and Aetiological Research Literature

**DOI:** 10.1371/journal.pone.0163721

**Published:** 2016-10-27

**Authors:** Ali Abbasi, Anna-Stina Sahlqvist, Luca Lotta, Julia M. Brosnan, Peter Vollenweider, Philippe Giabbanelli, Derek J. Nunez, Dawn Waterworth, Robert A. Scott, Claudia Langenberg, Nicholas J. Wareham

**Affiliations:** 1 Medical Research Council (MRC) Epidemiology Unit, University of Cambridge School of Clinical, Cambridge, United Kingdom; 2 GlaxoSmithKline, R&D, Stevenage, United Kingdom, RTP NC, King of Prussia, PA, United States of America; 3 Pfizer Inc, Cambridge, MA, United States of America; 4 Department of Medicine, Internal Medicine, CHUV, Lausanne, Switzerland; Deutsches Diabetes-Zentrum Leibniz-Zentrum fur Diabetes-Forschung, GERMANY

## Abstract

**Background:**

Blood-based or urinary biomarkers may play a role in quantifying the future risk of type 2 diabetes (T2D) and in understanding possible aetiological pathways to disease. However, no systematic review has been conducted that has identified and provided an overview of available biomarkers for incident T2D. We aimed to systematically review the associations of biomarkers with risk of developing T2D and to highlight evidence gaps in the existing literature regarding the predictive and aetiological value of these biomarkers and to direct future research in this field.

**Methods and Findings:**

We systematically searched PubMed MEDLINE (January 2000 until March 2015) and Embase (until January 2016) databases for observational studies of biomarkers and incident T2D according to the 2009 PRISMA guidelines. We also searched availability of meta-analyses, Mendelian randomisation and prediction research for the identified biomarkers. We reviewed 3910 titles (705 abstracts) and 164 full papers and included 139 papers from 69 cohort studies that described the prospective relationships between 167 blood-based or urinary biomarkers and incident T2D. Only 35 biomarkers were reported in large scale studies with more than 1000 T2D cases, and thus the evidence for association was inconclusive for the majority of biomarkers. Fourteen biomarkers have been investigated using Mendelian randomisation approaches. Only for one biomarker was there strong observational evidence of association and evidence from genetic association studies that was compatible with an underlying causal association. In additional search for T2D prediction, we found only half of biomarkers were examined with formal evidence of predictive value for a minority of these biomarkers. Most biomarkers did not enhance the strength of prediction, but the strongest evidence for prediction was for biomarkers that quantify measures of glycaemia.

**Conclusions:**

This study presents an extensive review of the current state of the literature to inform the strategy for future interrogation of existing and newly described biomarkers for T2D. Many biomarkers have been reported to be associated with the risk of developing T2D. The evidence of their value in adding to understanding of causal pathways to disease is very limited so far. The utility of most biomarkers remains largely unknown in clinical prediction. Future research should focus on providing good genetic instruments across consortia for possible biomarkers in Mendelian randomisation, prioritising biomarkers for measurement in large-scale cohort studies and examining predictive utility of biomarkers for a given context.

## Introduction

The pandemic of obesity and type 2 diabetes (T2D) is a global public health and health policy issue [1]. The development of T2D is a chronic process with approximately a decade-long latent period before the clinical onset of the disease [2]. Early identification of the diabetes risk provides an opportunity to introduce preventive interventions to stop or delay disease onset [3][4].

A large body of observational research over the past decade has shown that many biological markers or biomarkers, such as C-reactive protein, gamma-glutamyl transpeptidase or adiponectin, are associated with the risk of developing T2D [5][6][7][8][9]. Given the chronic and heterogeneous nature of diabetes, the use of biomarkers may help to better characterize diabetes risk and healthcare decision making [10][11]. In this context, the value of a new biomarker lies in whether it adds to prediction over and above simple clinical information [12]. However, biomarkers may also be of value in identifying causal pathways to diabetes risk which may in turn inform the development of new drug targets for preventive or therapeutic interventions [11]. In this situation it is not the predictive value that is of importance, but rather the extent to which any given biomarker is or is not causally associated with diabetes. Information about causal inference cannot usually be obtained from observational studies because of the problems of confounding [13][14]. Mendelian randomisation approaches can help to address this issue since the association of genetic variants with phenotypes should be unconfounded [14]. Previous reviews have reported one domain of research only, e.g., Mendelian randomisation or prediction research for a limited set of biomarkers [15][16], or the most recent study reviewed one specific class of biomarkers (i.e., metabolomics) [17]. In other words, to our knowledge, no comprehensive report has systematically identified and described a large number of biomarkers for the risk of developing T2D, highlighting what is known and unknown about many biomarkers like previously performed for prediction models for diabetes and diabetes-related outcomes [4][18,19]. Moreover, a more comprehensive approach as aggregated these domains is paramount to highlight the current state of literature of T2D biomarkers (including the most or the least studied biomarkers and the predictive and aetiological value of all these biomarkers) to guide prioritisation of future work.

We sought to systematically identify and characterize biomarkers that are associated with the risk of incident T2D in the population as a whole and within obesity strata. We did not include cross-sectional studies because of the limitations of such studies in enabling inferences about the direction of causality. A literature search was carried out to obtain the relevant studies which almost all reported biomarker-T2D associations over the recent decades. We also searched for meta-analyses, Mendelian randomisation studies, and prediction research studies on the biomarkers which were identified in the first review. We summarised the observational, prediction and aetiological evidence for all identified biomarkers in relation to T2D risk which not only summarises the existing evidence but presents a possible strategy for future studies.

## Methods

### Design, search strategy and eligibility

The systematic review was conducted according to the PRISMA guidelines (S1 Text), when applicable [20]. We searched PubMed for all published observational epidemiological studies that investigated the associations between biomarkers as the main exposure and incident (or new-onset) T2D as the primary outcome measure. The PubMed, which includes all MEDLINE and other non- MEDLINE journal titles, search was limited to date of publication from January 1^st^ 2000 until September 20^th^ 2013, and was updated in March 2015. We also searched Embase database until January 2016 (S2 Text). The three domains of search terms were formulated by three authors (AA, AS, CL) and focused on outcome (T2D), exposure (biomarkers) and study design (S3 Text). Given that there is a huge amount of literature on biomarkers in PubMed MEDLINE and Embase databases to identify relevant studies from 2000 through 2015 (S4 Text), and that three additional domains were included in our review, we can assure that updating and extending the search would not have material effect on the main strategy and conclusions.

**Fig 1:**
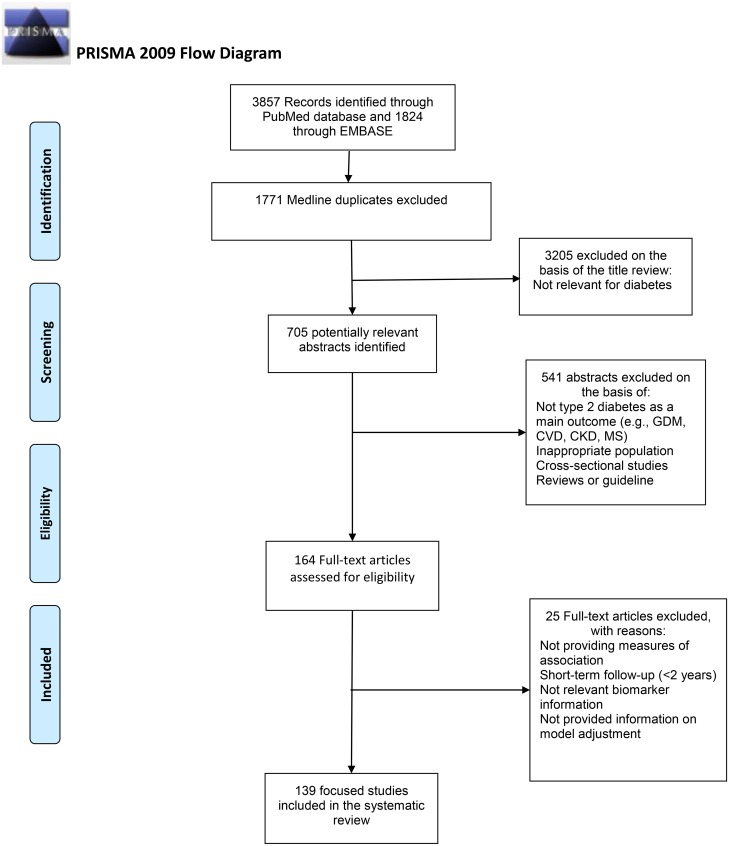
Flow Diagram of Studies Identified in the Literature Search for the Identification of Biomarkers for T2D.

We excluded studies that (i) applied a cross-sectional design (or a case-control design for prevalent diabetes); (ii) investigated only single nucleotide polymorphisms (SNPs); (iii) included only gestational diabetes mellitus or a complication of diabetes (like cardiovascular disease) as the main outcome; (iv) were restricted to individuals affected by other conditions (e.g., patients who have undergone organ transplantation, or had less than 2 years of follow-up); or (v) were narrative reviews or guidelines. Two reviewers (AA, AS) each screened half of the titles. Additionally, the reviewers (AA, AS) reviewed a random subset of 10% of each other’s studies to evaluate consistency of screening. After review of titles, three reviewers (AA, AS, LL) independently reviewed the abstracts to select relevant articles for the full text review. Differences in the studies selected by each reviewer were resolved by comparing the screened set of abstracts and repeating the reviews to reach consensus. A primary plan was made to extract necessary data from the full text of the retrieved original studies or to contact the corresponding author(s) when appropriate. Fig 1 depicts the work-flow of our systematic review.

### Data extraction and analysis

Three investigators (AA, AS, LL) extracted data from each study, including information on: 1) study characteristics (the first author’s name, name of study, year of publication, country, design, number of cases and population sample size, definition/ascertainment of T2D, duration of follow-up); 2) participants’ characteristics (measurement of biomarker(s) as the main exposure, age, sex, family history of T2D, body mass index (BMI) and smoking status, glucose level); 3) description of the study findings (biomarker assay and level, measure of association including odds ratio or hazard ratio or prognostic measures such as positive/negative predictive values, likelihood ratio or C-statistic, statistical model with or without multivariable adjustment, magnitude of an estimated effect, statistical significance, and stratified analysis by obesity strata).

Data were extracted independently and discrepancies were resolved by discussion. We tabulated extracted data on Excel spreadsheets. Descriptive analysis was performed for the participants’ characteristics and the measures of associations (between biomarker and T2D) when applicable. We calculated the relative risk difference (i.e., increase or reduction) which is approximately equal to 1 minus odds ratio or hazard ratio (when multiplied by 100) per one SD change in biomarker levels for T2D. Additionally, we searched for three other types of studies to summarize the data, including 1) meta-analyses of cohort studies that reported the observational evidence for associations between the identified biomarkers and T2D; 2) Mendelian randomisation studies that formally quantified the likelihood of a causal relationship between the identified biomarkers and T2D risk; and 3) the observational studies which examined predictive value of the identified biomarkers for T2D using classical statistical models [21].

After retrieving the relevant studies investigating the identified biomarkers in the first review, we summarized three other sources of data, termed “meta-analysis of observational studies”, “prediction research” and “Mendelian randomisation” studies. The strength of evidence for the biomarker-T2D associations and the quality of evidence were determined using the criteria for the strength and quality of studies in each domain.

#### Criteria for the strength of observational evidence

1. High: if ≥ 3 independent studies or a meta-analysis reported consistent evidence of a significant association when adjusted for level 2 that consists of age, sex (or sex-stratified), BMI (or waist circumference), and either family history of diabetes, smoking or hypertension.
2. Medium: if 1–3 independent studies reported consistent evidence of a significant association
3. Low or unknown: the remaining biomarkers

When multiple biomarkers were tested in one study, we checked whether the authors corrected for multiple testing. If not, we moved one level down. We also moved one level down if it did not meet any of items at high level.

#### Criteria for the strength of causality evidence

For the causal value of biomarkers, we applied the criteria to each biomarker as follows:

1. Potentially causal: > 50% of Mendelian randomisation studies reported causal effect of the biomarker on T2D
2. Likely non-causal: ≤ 50% of Mendelian randomisation studies reported causal effect of the biomarker on T2D
3. No evidence: There was no evidence from human studies.

**Criteria for the quality of Mendelian randomisation:** A score was calculated based on study size (0, total no. of cases <200; 1, no. of cases ≥200), number of study for each biomarker (0, only one study; 2, at least two independent studies), and reporting biomarker variance explained by genetic variant(s) (0, not reported; 1, variance reported) or power of Mendelian randomisation analysis (0, not reported; 1, reported). High quality means at least 3 out of 5 points and low quality less than 3 points.

#### Criteria for the strength of prediction evidence

For the predictive value of biomarkers, we applied the criteria to each biomarker as follows:

1. Potentially useful: There was an increase of ≥2% in the C-statistic and/or ≥ %10 NRI when a biomarker was added to the model of level 2
2. Modest/Null: There was an increase of <2% in the C-statistic, but statistically significant, up to 10% NRI or significant IDI when a biomarker was added to the model of level 2
3. No evidence: There was not clear or no statistically significant improvement in prediction

#### Criteria for the quality of prediction research

A score was calculated based on study size (0, total no. of cases <200; 1, no. of cases ≥200), number of study for each biomarker (0, only one study; 2, at least two independent studies) and measure of prediction (0, only one measure reported; 2, at least two measures reported). High quality means at least 3 out of 5 points and low quality less than 3 points.

P values <0.05 (from two-tailed tests) were considered statistically significant. For the practically of the review, we accounted for multiple comparisons using Bonferroni corrections, where evidence was reported for multiple biomarkers associated with diabetes in a single study. All statistical analyses were conducted with Stata/SE version 13.1 (Stata Corp, College Station, Texas) or R version 3.0.3 (Vienna, Austria) for Windows (http://cran.r-project.org/). The software package Tableau 8.2 was used to visualize data.

## Results

### Systematic review and study characteristics

We scanned 3910 titles and selected 705 abstracts for review. We reviewed 164 papers in detail to retrieve data from 139 relevant studies describing biomarker(s) in relation to the incident T2D (Fig 1 and S1 Table). Nine studies were excluded because they had a cross-sectional design, four had a small sample of participants affected by other conditions, and 13 did not report a formal measure of association or they did not report results for specific biomarkers (S2 Table).

**Fig 2:**
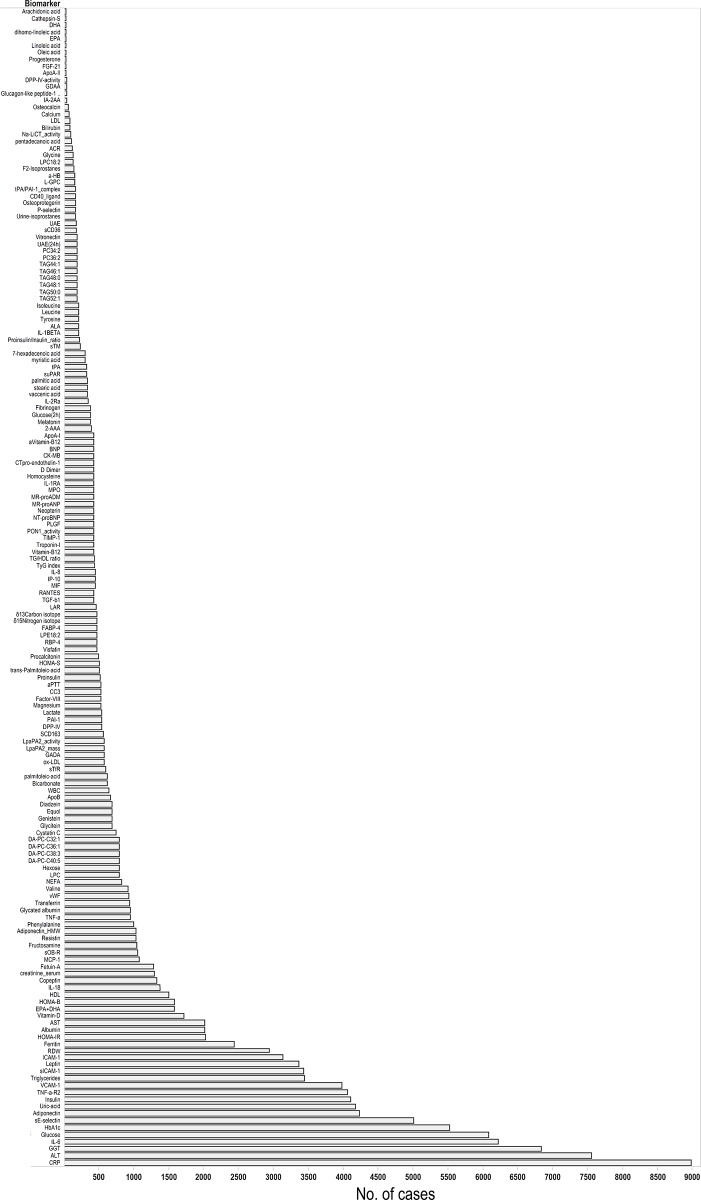
Identified Biomarkers for the Risk of Developing Type 2 Diabetes. The length of bars denotes total number of incident type 2 diabetes cases across all studies for each biomarker.

**Fig 3:**
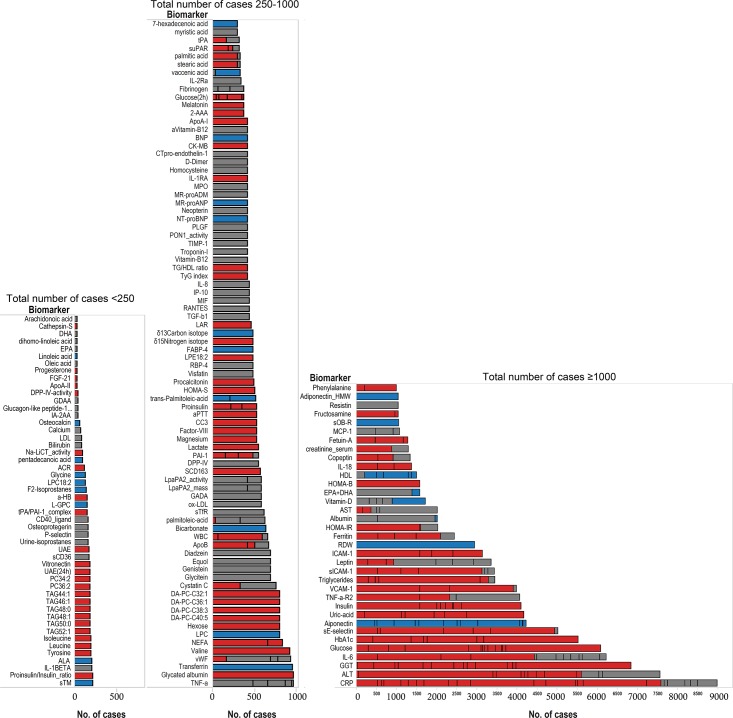
Association of Biomarkers with the Risk of Developing Type 2 Diabetes. The length of bars denotes total number of incident type 2 diabetes cases from all studies for each biomarker. Each bar is divided by the solid vertical lines which correspond to different study for each biomarker. Red colour indicates a positive statistically significance, blue colour an inverse statistically significance and grey colour a null association. Panel a) total number of cases up to 250; b) total number of cases between 250 and 1000; and panel c) total number of cases above 1000.

S1 Table summarises the characteristics of the 139 studies, 52 of which were from the US and 65 from Europe. Before 2007, between three and six studies per year were published. However, since then the publication rate has increased with 21 studies being reported in 2012 alone. From these 139 articles describing over 372 biomarkers, we identified 167 unique biomarkers which had been evaluated at least once (Figs 2 and 3; S3 Table). Among these, four studies have investigated the associations between urinary markers (including urine isoprostanes, urinary albumin excretion and urine albumin/creatinine ratio) and incident T2D (S3 Table).

Fig 2 shows the total number of T2D cases across all studies. In Fig 3, we stratify the biomarkers in those studies with a total of less than 250 cases, between 250 and 1000 cases, and more than 1000 cases. For instance, small biomarker studies correspond to the total number of cases across all studies, but not to single small studies only. The figure also shows (in the horizontal bars) the number of studies reported for each biomarker which varies from one for some biomarkers to 23 for C-reactive protein. The studies were performed in diverse populations, but most were cohort studies which included middle-aged men and women who were followed for up to 20 years with the largest study including 3,352 T2D cases and 155,904 total participants. The authors mainly used more than one information source to define incident T2D. Most studies (n = 128) provided information about BMI (the mean ranged from 23 for total to 32 for those who developed T2D), and some reported other characteristics such as family history of T2D (n = 64). Data on glucose (fasting or 2-hour glucose) were available in many of the studies (n = 90).

### Biomarkers for T2D risk

In S3 Table, we provide detailed information for the biomarkers that were measured. Most studies used enzymatic methods or enzyme-linked immunosorbent assays (ELISA) to measure biomarkers. However, we identified different methods for the same biomarker (e.g. C-reactive protein) across studies. All studies, except one, reported adjusted risk estimates for T2D per quantile and/or expressed risk for continuous data per unit, per log transformed unit or for one SD difference in the level of the biomarkers. We extracted data from the most adjusted model in each case to reduce the potential for confounding.

Fifty (29.9%) of the biomarkers were reported in a total number of cases <250 and therefore the evidence was based on small scale studies. Across this limited evidence base, 66% of the individual studies reported a statistically significant association with T2D. Eighty two (49.1%) of the biomarkers were reported from studies with a total number of cases between 250 and1000, in which 53% of individual studies described associations that reached statistical significance. The remaining 35 (21%) biomarkers were studied on a large scale with a total number of cases ≥1000 in which 76.6% of the individual studies reported associations that reached statistical significance (Fig 3). The colour coding in Fig 3 shows studies that describe a positive association (red), an inverse relationship (blue), or a relationship that is not statistically significant in either direction (grey). In the medium scale evidence category, a greater number of biomarkers have evidence which is uncertain. In the large scale evidence category, there is general consistency on the direction of association, particularly for biomarkers such as C-reactive protein and gamma-glutamyl transpeptidase which have been reported in numerous papers [5,6][8]. In other words, these associations looked similar across the earlier and later studies.

There is considerable heterogeneity in how the magnitude of association is described which makes meta-analysis across studies for any individual biomarker difficult without contacting the authors of specific papers to get additional information. In studies in which the risk estimate was expressed per standard deviation, there was considerable variation in the strength of association ranging from 2.0% to 102% (corresponding to an odds ratio of 2.02 for T2D risk per each 1-SD increment in phenylalanine [22]) per 1-SD difference in the biomarker level. In some studies, risk estimates were calculated across biomarker percentile, e.g., a 4-fold or 5-fold increased risk of T2D when comparing the top versus bottom quartile of C-reactive protein or gamma-glutamyl transpeptidase, respectively [5][6][8].

Stratified analysis within categories of BMI or a study of the interaction with BMI, was undertaken with 17 studies of 19 individual biomarkers. The definition of obesity strata varied between studies and 12 biomarkers were statistically associated with diabetes risk in the overweight or obese stratum as defined in each study (S4 Table). The studies were too different in design to meta analyse. In other words, we could not formally assess the extent of publication bias and the heterogeneity of the studies because marked differences in the populations with varied settings included, the methods used to measure biomarkers, the confounding factors considered and the approach to deriving risk estimates created significant challenges for pooling point estimates or p values across studies.

### Aetiological and predictive value of biomarkers

**Fig 4:**
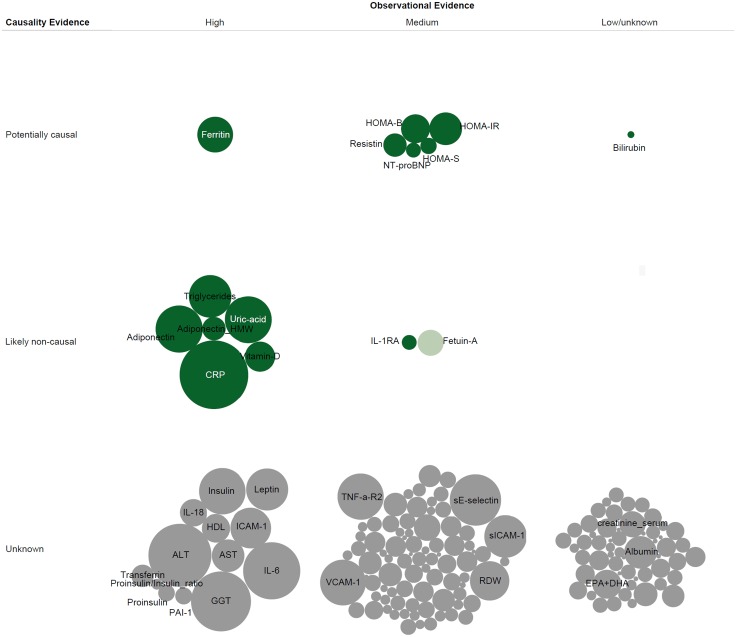
Observational and Causality Evidence for Biomarker-Type 2 Diabetes Relation. The size of circles denotes total number of incident type 2 diabetes cases from all observational studies for each biomarker. Data were synthesised from observational evidence, either from the initial review or published meta-analysis studies of the identified biomarkers, and from the Mendelian randomisation studies for the identified biomarker and the risk of developing type 2 diabetes. Dark green colour indicates high quality Mendelian randomisation study, light green colour low quality study, and grey colour no evidence in the literature as defined (see the method section).

In our review of the use of Mendelian randomisation approaches, we identified 17 studies (S5 Table) that examined the causal inference of the association of 14 blood-based biomarkers with T2D from the initial set of 167 biomarkers. Fig 4 shows the strength of evidence of association between the biomarker and T2D from the observational studies, either in the first review or published meta-analyses of the 167 identified biomarkers (S3, S6 and S7 Tables), and a summary of the evidence that the association is as a result of a causal relationship assessed using Mendelian randomisation approaches for 14 biomarkers (S5 Table). For many biomarkers, particularly those where the observational association was only recently described, such as those emerging from metabolomics studies, the causal nature of the biomarker to T2D relationship has not been investigated. These studies are coloured grey in Fig 4. Twelve of these biomarkers which have not been investigated formally using Mendelian randomisation approaches had strong observational evidence of association and would therefore be a priority set for future Mendelian randomisation studies. Of the remaining biomarkers for which Mendelian randomisation approaches have not yet been undertaken, many have only been studied in small or medium sized observational studies creating a strategy choice between investing in large observational studies or Mendelian randomisation studies where good genetic instruments exist for the given biomarker. For those few biomarkers for which the Mendelian randomisation approach has been used, the majority (adiponectin [23,24], C-reactive protein [25], Fetuin-a [26], triglycerides [27], vitamin D [28,29], IL-1Ra [30] and uric acid [31]) have evidence that is consistent with the biomarker not being causally related to T2D risk. The rest of the biomarkers, including those related to beta-cell function [32,33], HOMA-IR [33], ferritin (Transmembrane protease serine 6)[34], homocysteine [35], N-terminal pro B-type natriuretic peptide (NT-proBNP)[36], bilirubin [37] and resistin [38] were claimed to be causally associated with T2D (S5 Table).

**Fig 5:**
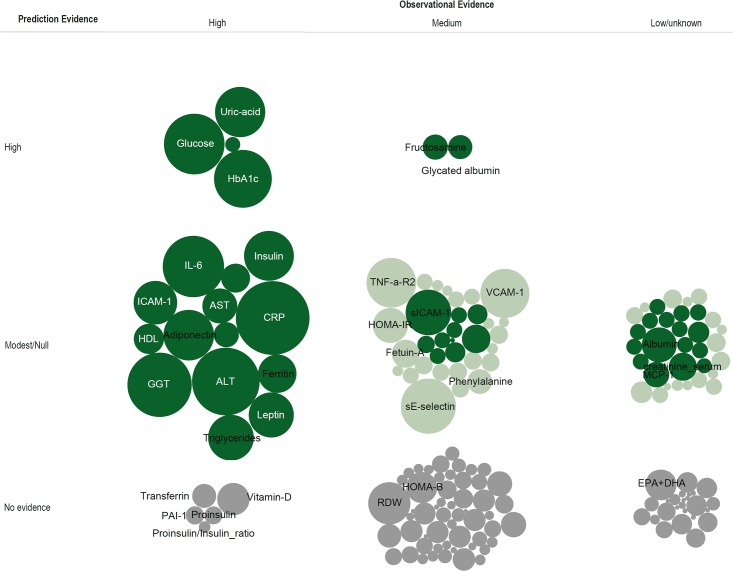
Observational and Prediction Evidence for Biomarker-Type 2 Diabetes Relation. The size of circles denotes total number of incident type 2 diabetes cases from all observational studies for each biomarker. Data were synthesised from observational evidence, either from the initial review or published meta-analysis studies of the identified biomarkers, and from the prediction research studies for the identified biomarker and the risk of developing type 2 diabetes. Dark green colour indicates high quality prediction research study, light green colour low quality study, and grey colour no evidence in the literature as defined (see the method section).

Additionally, we characterised studies which aimed to evaluate the predictive value of 85 blood-based biomarkers (out of 167) for T2D risk (S7 and S8 Tables). In Fig 5, we plotted the strength of observational evidence of association for each biomarker against an assessment of its predictive utility. In the column including those biomarkers deemed to have strong observational evidence of association, it is clear from the size of the circle (which indicates the total number of cases studied) that considerable attention has been given to undertaking repeated studies where the predictive value of the biomarkers being studied is limited. There are 5 biomarkers with strong evidence of observational association for which there is currently no formal evidence of predictive utility (transferrin, vitamin D, PAI-1, proinsulin and proinsulin to insulin ratio). There is a group of biomarkers that have strong evidence of association with T2D, but also high and medium-level predictive utility, and 5 of these 6 are markers of the glycaemic pathway, namely fasting or 2 hour glucose, HbA1c [4], fructosamine and glycated albumin [39]. Uric acid is the only non-glycaemic biomarker that has strong evidence of association and predictive utility [40][41][4]. The remaining biomarkers either had no or modest predictive value for the risk of future T2D (such as novel biomarkers such as branched chain amino acids) or have not yet been studied for prediction [22][4].

## Discussion

This systematic review of observational cohort studies of the association between biomarkers and incident T2D clearly shows that this is a rapidly developing field. Our review included 139 papers, of which 73% have been published in the past 7 years. Most papers have assessed blood-based biomarkers; and only four papers have reported the association of a urinary biomarker with incident T2D. Not only are there a greater number of papers and thus an increasing total number of cases across studies, but there is also a rapid expansion in the number of biomarkers being reported. This makes it very timely to undertake a systematic review to describe the current state of the literature, to inform the strategy for future interrogation of existing and newly described biomarkers, to direct future efforts in this field, and to provide a framework for interpreting the information presented. We have made a very deliberate distinction in this review between the interpretation of biomarkers as indicators of underlying pathophysiological pathways to diabetes, as opposed to the assessment of their utility in clinical prediction [21][42].

In the situation where the association of a biomarker with diabetes is thought to be a possible manifestation of an underlying pathway to disease, the magnitude of the association between the biomarker and diabetes is one consideration, but it is also possible for an association to be relatively weak, but still reflect a causal relationship. In this context, the question of confounding is paramount. In observational studies it is impossible to fully account for confounding factors that have been accounted for and included in the analyses because measurement error of those confounding factors will always leave the possibility of residual confounding [13][14]. There is also the problem of factors that have not been considered—unmeasured confounding [42]. Whilst randomised controlled trials are an effective strategy for dealing with both forms of confounding, the likelihood that this approach could help with the interpretation of biomarkers is limited since any intervention would need to be highly specific to the biomarker in question. In the face of these challenges, Mendelian randomisation is emerging as a useful strategy for assessing whether a biomarker-to-T2D relationship is or is not likely to be causal [13,14]. In our review, we identified such an observation for NT-proBNP for which the observed relative risk difference was 18% for T2D for a 1-SD increase in log-transformed biomarker [36]. Even though this observational evidence suggests that the relationship between NT-proBNP is relatively weak, the genetic association suggests that it is compatible with a causal relationship [36].

By contrast, in the context of clinical prediction, the strength of the association in terms of its effect size (and not the measure of statistical significance) is a very important determinant of the value that a biomarker has in adding to prediction [21][12]. Here, the value of a new biomarker lies in whether it adds to prediction, which is at heart a practical activity, over and above clinical information and glycaemia measures. The issue of confounding is less of a concern, and a biomarker as a predictive tool does not necessarily need to be causally related to the disease outcome. An example from our review is uric acid, which has strong evidence of association and predictive utility [4][40,41]. However, Mendelian randomisation did not provide evidence of a causal link between uric acid and T2D [31].

### Implications for enhancing understanding of the aetiology and pathogenesis of T2D

In our review, many published reports of biomarkers, particularly novel ones, were from single studies or small cohorts with limited statistical power, and thus the observational evidence of association for these biomarkers is weak. One of the key strategic questions in the field is how to enhance aetiological understanding of the growing number of novel biomarkers for which association with diabetes is being reported. Meta-analysis of the emerging published literature could be one approach, but if that literature is based predominantly on small studies, there is a strong likelihood that publication bias could affect the estimate of effect size. The conduct of large prospective cohort studies which have sufficient power by virtue of having a large number of incident cases is a preferable approach, but this is an expensive strategy both in financial terms and in sample use, particularly if a very wide range of possible biomarkers is to be considered. Given the considerable cost of measuring biomarkers in all cohort participants, a nested case-control or case-cohort sampling design would be preferred, providing a cost-effective tool.[43] It would be possible to use Mendelian randomisation approaches to prioritise biomarkers for investigation in cohort studies [14,42].

Currently most Mendelian randomisation studies that we reviewed have first defined a biomarker as definitively associated with incident diabetes and have then investigated the likelihood that the relationship is causal. In our review we have observed that this approach has most frequently been undertaken for biomarkers like C-reactive protein, adiponectin and triglycerides for which the available evidence suggests that the observed associations with incident diabetes are more likely to be a manifestation of confounding rather than an underlying causal relationship [23,24,27,44]. There are a group of biomarkers for which there is strong or medium evidence of observational association which could constitute a prioritized group of biomarkers for evaluation by Mendelian randomisation. The use of Mendelian randomisation approaches for the very large group of biomarkers for which there is weak or no evidence of association would be logical if one could first find strong genetic instruments for these biomarkers, which could be done in a cross-sectional study. We did not find any Mendelian randomisation analyses for urinary biomarkers and incident T2D. One could then examine the association of these genetic instruments with diabetes in accessible databases from large genetic consortia [45], and then determine which biomarkers to prioritise for measurement in prospective cohort studies on the basis of the likelihood of a causal association. It is worth mentioning that Mendelian randomization can be confounded by pleiotropic effects of genetic variants and for the possibility of developmental compensation, called canalization.

### Implications for prediction of T2D

Previously, we and others have systematically identified and validated existing prediction models for the risk of future T2D [4,46,47][48][49][50]. These studies suggest that models which include easily measured factors such as age, sex, BMI and family history of diabetes plus additional information on glucose or HbA1c have slightly larger discriminatory power than basic models without the clinical chemistry measurements [4][46]. The hope of groups investigating other biomarkers is that they will enhance prediction further. Our review suggests that over a time horizon of 5–10 years, additional biomarkers have failed to improve prediction metrics over a model consisting of traditional diabetes predictors, glucose and HbA1c [51][4][40,52] which are, of course, measures used in the diagnosis of diabetes. In this context, the utility of biomarkers (or variables) is clinically-relevant for predicting the future risk of T2D [4,16,48,49], while individuals are not necessarily in prediabetes state. The predictive value of a limited set of urinary biomarkers-among identified those in our review-for T2D has not been evaluated.

One challenge is the discordance between how biomarkers are assessed in observational studies with how they are used in a real-world context to aid prediction. Most research studies assess marginal additions in discriminatory power and other prediction metrics in a situation where all individuals have all measurements [21][12]. However, outside of the research setting it is much more likely that tests are used somewhat differently. In real-world practice, biomarker tests are not offered to all people, but are used to refine the prediction of diabetes risk in a stepwise manner for people deemed to be at intermediate or high risk on the basis of existing reliable and simple models. One conclusion from our review is that future research on the predictive utility of novel biomarkers should ensure that the assessment is undertaken in the context in which they are likely to be used (e.g., clinical or public health practice).

### Strengths and limitations

To the best of our knowledge, this is the first systematic review of observational studies from 69 cohorts worldwide investigating the associations between biomarkers and the risk of developing T2D in the general population. In this review, three researchers were involved in reaching consensus on the search terms, inclusion criteria and data extraction, with a random manner of re-checking of the titles and abstracts. To minimise the impact of potential biases, (e.g., selection bias in cross-sectional studies), we included data from observational cohort, case-cohort or nested case-control studies. We excluded studies that were restricted to individuals affected by other clinical conditions, or those with short-term follow-up. Thus, we consider that our strategy provides evidence that is generalizable to populations of adults at risk of developing diabetes in real-world settings. The risk of T2D is markedly higher in obese people and one key question is how biomarkers can assist in the prediction of incident diabetes in this sub-group or how they can inform aetiological understanding of the metabolic consequences of obesity. However, our review was not confined to studies of obese participants because many studies of the general population include secondary analyses by adiposity strata.

Many of the studies of the prediction of future diabetes will have included at baseline people who had existing, but undiagnosed diabetes [4][46]. However, in the context of prediction this is not a major issue since it reflects the clinical reality, since risk tools applied to populations to predict future risk will also help identify those with prevalent but undiagnosed diabetes. The question of inclusion of people with prevalent but undiagnosed diabetes at baseline is more of an issue for aetiological studies because of the problem of reverse causality [13,42]. Most of the studies we reviewed used more than one information source to ascertain T2D, and a number of studies employed several methods to verify potential incident cases [4,46].

We observed a lack of comprehensive information about assay reliability, particularly for emerging biomarkers, in those studies included in the review. Our ability to synthesise data across studies is limited by the lack of comprehensive data in these studies on how biomarkers vary over time and on the potential instability of biomarkers in blood or urine samples, which have been stored for several years. Our review process may have failed to identify other biomarkers (e.g., testosterone, a sex hormone [53]) or all studies on the same biomarker if the authors did not explicitly mention the term “biomarker” explicitly or a related MeSH term. Since we used biomarker or its related MeSH terms in our search strategy, our literature review did not include all biomarkers and relevant studies for incident T2D [54,55]. Most authors reported one or two biomarkers, and it is likely that small studies which observe statistically significant results are more likely to be submitted and subsequently published. Moreover, the degree of differences in measurement, reporting findings and the variation in design and analytical approach to adjust for confounding across studies did not allow us to formally pool the estimates. Finally, we defined different criteria to evaluate the strength and quality of observational evidence, prediction and Mendelian randomisation studies; however, further work is needed to evaluate and complement our prioritization approach.

### Future directions towards use of biomarkers

In this review, we have observed a rapid increase in the number of studies investigating biomarkers in relation to the prediction of T2D. Unfortunately, many of the reports do not contribute meaningfully to our understanding about how to improve clinical prediction, nor do they provide robust evidence about the roles of biomarkers as measures of causal pathways leading to T2D. In the context of clinical prediction, robust evidence of predictive utility only exists for six biomarkers, five of which are measures of the glycaemic process itself [4][39]. In general, too few biomarkers have been tested formally for predictive utility in the context in which they might be used clinically. Finally, future work is needed to investigate predictive value of biomarkers in high risk individuals by combining clinical information such as age, sex and BMI rather than measuring biomarkers in all people [56,57].

The aetiological evidence base is also relatively weak since many observational studies have been underpowered and cannot definitively conclude that a given biomarker is associated with future risk of T2D, and even when there is evidence of association, the problems of confounding in classical observational studies make it difficult to identify the unconfounded association of a biomarker with diabetes [13,14,42]. Currently fewer than 10% of the 167 biomarkers identified in our review have been assessed using complementary genetic data in Mendelian randomisation studies, largely because of the absence of evidence from genome-wide association studies providing strong genetic instruments for possible biomarkers [45]. However, we would predict that this body of evidence will accumulate rapidly and will quickly transform our understanding about which of the many biomarkers associated with diabetes are likely to reflect causal pathways. Given the limitations of Mendelian randomisation (e.g., the lack of suitable genetic instruments for biomarkers of interest) and that common genetic variants represent only a small effect on biomarker level per se, a large sample size with sufficient statistical power is required to reliably interpret such findings [58,59].

## Conclusions

Although varying biomarkers have been associated with the incidence of T2D after accounting for traditional diabetes risk factors, evidence about the likelihood of causality of these relationships is lacking for most biomarkers. Integration of observational epidemiological and genetic data can be used to examine the likelihood of causality and this may assist in the identification of causal and potentially modifiable pathways to diabetes. Only half of biomarkers have been examined for T2D prediction. Apart from glycaemic biomarkers, the value of most examined biomarkers for risk prediction of T2D in clinical practice is still doubtful. Future research should focus on providing good genetic instruments across consortia for possible biomarkers in Mendelian randomisation, prioritising biomarkers for measurement in large-scale cohort studies and examining predictive utility of biomarkers for a given context.

## Supporting Information

S1 Text PRISMA Checklist. (DOC)

S2 Text Inclusion Criteria. (DOC)

S3 Text Search Terms. (DOC)

S4 Text References. (DOC)

S1 Table General Characteristics of 139 Studies. (DOC)

S2 Table Excluded Studies. (DOC)

S3 Table 167 Biomarkers for Risk of Incident Type 2 Diabetes. (DOC)

S4 Table Associations of Biomarkers with Risk of Incident Type 2 Diabetes by BMI Strata. (DOC)

S5 Table 17 Mendelian Randomisation Studies of the Identified Biomarkers for Risk of Incident Type 2 Diabetes. (DOC)

S6 Table 20 Meta-analysis Studies of the Identified Biomarkers for Risk of Incident Type 2 Diabetes. (DOC)

S7 Table An Overview of Observational, Prediction and Mendelian Randomization Research for Biomarkers and Incident Type 2 Diabetes. (XLS)

S8 Table 51 Prediction Studies of the Identified Biomarkers for Risk of Incident Type 2 Diabetes. (DOC)
