# Supplementary material for: A Systematic Review of Biomarkers and Risk of Incident Type 2 Diabetes: An Overview of Epidemiological, Prediction and Aetiological Research Literature
Source: PLoS One. 2016 Oct 27;11(10):e0163721. doi: 10.1371/journal.pone.0163721 (PMC5082867; doi:10.1371/journal.pone.0163721)
Supplement: S2 Text — (DOC) [file pone.0163721.s002.doc]

**S2 Text. Inclusion Criteria.** Studies were included if they met the following criteria:

1) They formally quantified the association between biomarker(s) and type 2 diabetes (T2D) risk,

2) T2D was considered as the main outcome or one of main outcomes, for example, the authors calculated risk estimates for incident T2D together with other metabolic traits/disorders,

3) The exposure can be classified or defined as biomarkers that have been objectively measured and evaluated as a biochemical clue to the pathobiological processes, or pharmacological responses to a therapeutic intervention in blood (serum or plasma) or urine. In addition to studies explicitly measuring biomarkers, relevant studies investigating dietary variables were also included. We have reviewed the full articles, because the authors of those studies might not have mentioned in the title or abstract whether they actually measured levels of dietary biomarkers corresponding to specific food intake.

4) The design of the observational study was retrospective/prospective cohort, case-cohort and nested case-control. Case-cohort is a longitudinal study within a cohort study, consisting of all cases (the individuals who developed T2D during follow-up time) and a random subset of the total cohort. Nested case-control is a longitudinal study within a cohort study, consisting of all cases and a subset of those who did not develop disease (i.e. remained free of T2D by the end of follow-up).

We also included some other studies without abstract, for example published as a research letter in PubMed, reporting an association between biomarkers and risk of T2D in the titles.
