# Supplementary material for: A Systematic Review of Biomarkers and Risk of Incident Type 2 Diabetes: An Overview of Epidemiological, Prediction and Aetiological Research Literature
Source: PLoS One. 2016 Oct 27;11(10):e0163721. doi: 10.1371/journal.pone.0163721 (PMC5082867; doi:10.1371/journal.pone.0163721)
Supplement: S3 Text — (DOC) [file pone.0163721.s003.doc]

**S3 Text. Search terms.** We applied the terms in PubMed all MEDLINE and other non-MEDLINE titles (26000 records) Embase database to identify biomarkers in relation to risk of developing T2D.

**Database: MEDLINE via PubMed**

|  | Search Terms |
| --- | --- |
| [#4](http://www.ncbi.nlm.nih.gov/pubmed/advanced) | #1 AND #2 AND #3 |
| [#3](http://www.ncbi.nlm.nih.gov/pubmed/advanced) | "Cohort Studies"[Mesh] OR Cohort Study OR Studies, Cohort OR Study, Cohort OR Studies, Historical Cohort OR Cohort Studies, Historical Cohort Study, Historical OR Historical Cohort Study OR Study, Historical OR Analysis, Cohort OR Analyses, Cohort OR Cohort Analyses OR Cohort Analysis OR Incidence Studies OR Incidence Study OR Studies, Incidence OR Study, Incidence OR "Incidence"[Mesh] OR "Cohort Studies"[Mesh] OR Case-cohort study OR Nested case-control study OR Survey |
| [#2](http://www.ncbi.nlm.nih.gov/pubmed/advanced) | "Biological Markers"[Mesh] OR Markers, Biological OR Biomarkers OR Marker, Biological OR Biological Marker OR Biologic Marker OR Marker, Biologic OR Biologic Markers OR Markers, Biologic OR Markers, Clinical OR Clinical Markers OR Marker, Clinical OR Clinical Marker OR Markers, Serum Markers OR Markers, Serum OR Serum Marker OR Marker, Serum OR Biochemical Marker OR Marker, Biochemical OR Markers, Biochemical OR Biochemical Markers OR Markers, Laboratory OR Laboratory Markers OR Marker, Laboratory OR Laboratory Marker OR "Metabolome"[Mesh] OR Metabolomes OR Metabolic Profile OR Metabolic Profiles OR Profile, Metabolic OR Profiles, Metabolic OR "Metabolomics"[Mesh] OR Metabolomic OR Metabonomics OR Metabonomic OR "Proteome"[Mesh] OR Proteomes OR "Proteomics"[Mesh] |
| [#1](http://www.ncbi.nlm.nih.gov/pubmed/advanced) | “Diabetes mellitus, Type 2”[Mesh] OR NIDDM OR Maturity-Onset Diabetes OR Diabetes Mellitus, Noninsulin-Dependent OR Diabetes Mellitus, Adult-Onset OR Adult-Onset Diabetes Mellitus OR Diabetes Mellitus, Adult Onset OR Diabetes Mellitus, Maturity-Onset OR Diabetes Mellitus, Maturity Onset OR Diabetes Mellitus, Non-Insulin Dependent OR Diabetes Mellitus, Non-Insulin-Dependent OR Non-Insulin-Dependent Diabetes Mellitus OR Diabetes Mellitus, Noninsulin Dependent OR Diabetes Mellitus, Slow-Onset OR Diabetes Mellitus, Slow Onset OR Slow-Onset Diabetes Mellitus OR Diabetes Mellitus, Stable OR Stable Diabetes Mellitus OR Diabetes Mellitus, Type II OR Maturity-Onset Diabetes Mellitus OR Maturity Onset Diabetes Mellitus OR MODY OR Type 2 Diabetes Mellitus OR Noninsulin-Dependent Diabetes Mellitus OR “Type 2 Diabetes”[tiab] OR “Diabetes”[ti] |
|  |  |
|  | (#1 AND #2 AND #3) AND 2000/01/01:2013/12/31[DP] |
|  | Search ((#1 AND #2 AND #3) AND 2000/01/01:2013/12/31[DP]) NOT ("Diabetes Mellitus, Type 1"[MeSH] OR "type 1 diabetes"[ti])) |

**Database: EMBASE**

|  | Search Terms |
| --- | --- |
| [#4](http://www.ncbi.nlm.nih.gov/pubmed/advanced) | #1 AND #2 AND #3 |
| [#3](http://www.ncbi.nlm.nih.gov/pubmed/advanced) | 'Cohort Studies' OR 'Cohort Study' OR 'Cohort Analyses' OR 'Cohort Analysis' OR 'Incidence Studies' OR 'Incidence Study' OR 'Case-cohort study' OR 'Nested case-control' OR 'Survey' |
| [#2](http://www.ncbi.nlm.nih.gov/pubmed/advanced) | 'Biological Markers' OR 'Biomarkers' OR 'Biological Marker' OR 'Biologic Marker' OR 'Biologic Markers' OR 'Clinical Markers' OR 'Clinical Marker' OR 'Serum Markers' OR 'Serum Marker' OR 'Biochemical Marker' OR 'Biochemical Markers' OR 'Laboratory Markers' OR 'Laboratory Marker' OR 'Metabolome' OR 'Metabolomes' OR 'Metabolic Profile' OR 'Metabolic Profiles' OR "Metabolomics" OR Metabolomic OR Metabonomics' OR 'Metabonomic' OR 'Proteome' OR 'Proteomes' OR 'Proteomics' |
| [#1](http://www.ncbi.nlm.nih.gov/pubmed/advanced) | 'Type 2 Diabetes mellitus' OR 'NIDDM' OR 'Maturity-Onset Diabetes' OR 'Adult-Onset Diabetes Mellitus' OR 'Diabetes Mellitus, Maturity Onset' OR 'Non-Insulin-Dependent Diabetes Mellitus' OR 'Slow-Onset Diabetes Mellitus' OR 'Stable Diabetes Mellitus' OR 'Type II Diabetes Mellitus' OR 'Maturity-Onset Diabetes Mellitus' OR 'Maturity Onset Diabetes Mellitus' OR 'MODY' OR 'Noninsulin-Dependent Diabetes Mellitus' OR ' Type 2 Diabetes' OR 'Diabetes' |
|  |  |
|  | 3 AND #2 AND #1 AND [humans]/lim AND [1-1-2000]/sd NOT [12-31-2015]/sd |
