# Supplementary material for: A Systematic Review of Biomarkers and Risk of Incident Type 2 Diabetes: An Overview of Epidemiological, Prediction and Aetiological Research Literature
Source: PLoS One. 2016 Oct 27;11(10):e0163721. doi: 10.1371/journal.pone.0163721 (PMC5082867; doi:10.1371/journal.pone.0163721)
Supplement: S4 Text — (DOC) [file pone.0163721.s004.doc]

**S4 Text. References**.

1. Abbasi A, Bakker SJ, Corpeleijn E, et al. Liver function tests and risk prediction of incident type 2 diabetes: evaluation in two independent cohorts. PloS one. 2012;7(12):e51496.

2. Abbasi A, Corpeleijn E, Meijer E, et al. Sex differences in the association between plasma copeptin and incident type 2 diabetes: the Prevention of Renal and Vascular Endstage Disease (PREVEND) study. Diabetologia. 2012;55(7):1963-1970.

3. Abbasi A, Corpeleijn E, Postmus D, et al. Plasma procalcitonin and risk of type 2 diabetes in the general population. Diabetologia. 2011;54(9):2463-2465.

4. Abbasi A, Deetman PE, Corpeleijn E, et al. Bilirubin as a potential causal factor in type 2 diabetes risk: a Mendelian randomization study. Diabetes. 2015;64(4):1459-1469.

5. Abbasi A, Peelen LM, Corpeleijn E, et al. Prediction models for risk of developing type 2 diabetes: systematic literature search and independent external validation study. BMJ (Clinical research ed.). 2012;345:e5900.

6. Afzal S, Bojesen SE, Nordestgaard BG. Low 25-hydroxyvitamin D and risk of type 2 diabetes: a prospective cohort study and metaanalysis. Clinical chemistry. 2013;59(2):381-391.

7. Alessi MC, Nicaud V, Scroyen I, et al. Association of vitronectin and plasminogen activator inhibitor-1 levels with the risk of metabolic syndrome and type 2 diabetes mellitus. Results from the D.E.S.I.R. prospective cohort. Thrombosis and haemostasis. 2011;106(3):416-422.

8. Alyass A, Almgren P, Akerlund M, et al. Modelling of OGTT curve identifies 1 h plasma glucose level as a strong predictor of incident type 2 diabetes: results from two prospective cohorts. Diabetologia. 2015;58(1):87-97.

9. Andre P, Balkau B, Born C, et al. Hepatic markers and development of type 2 diabetes in middle aged men and women: a three-year follow-up study. The D.E.S.I.R. Study (Data from an Epidemiological Study on the Insulin Resistance syndrome). Diabetes & metabolism. 2005;31(6):542-550.

10. Balkau B, Lange C, Fezeu L, et al. Predicting diabetes: clinical, biological, and genetic approaches: data from the Epidemiological Study on the Insulin Resistance Syndrome (DESIR). Diabetes care. 2008;31(10):2056-2061.

11. Becerra-Tomas N, Estruch R, Bullo M, et al. Increased serum calcium levels and risk of type 2 diabetes in individuals at high cardiovascular risk. Diabetes care. 2014;37(11):3084-3091.

12. Bobbert T, Schwarz F, Fischer-Rosinsky A, et al. Fibroblast growth factor 21 predicts the metabolic syndrome and type 2 diabetes in Caucasians. Diabetes care. 2013;36(1):145-149.

13. Brantsma AH, Bakker SJ, Hillege HL, et al. Urinary albumin excretion and its relation with C-reactive protein and the metabolic syndrome in the prediction of type 2 diabetes. Diabetes care. 2005;28(10):2525-2530.

14. Brunner EJ, Kivimaki M, Witte DR, et al. Inflammation, insulin resistance, and diabetes--Mendelian randomization using CRP haplotypes points upstream. PLoS medicine. 2008;5(8):e155.

15. Buijsse B, Boeing H, Hirche F, et al. Plasma 25-hydroxyvitamin D and its genetic determinants in relation to incident type 2 diabetes: a prospective case-cohort study. European journal of epidemiology. 2013;28(9):743-752.

16. Burgess S, Timpson NJ, Ebrahim S, Davey Smith G. Mendelian randomization: where are we now and where are we going? International journal of epidemiology. 2015;44(2):379-388.

17. Cameron AJ, Zimmet PZ, Soderberg S, et al. The metabolic syndrome as a predictor of incident diabetes mellitus in Mauritius. Diabetic medicine : a journal of the British Diabetic Association. 2007;24(12):1460-1469.

18. Carnethon MR, Palaniappan LP, Burchfiel CM, Brancati FL, Fortmann SP. Serum insulin, obesity, and the incidence of type 2 diabetes in black and white adults: the atherosclerosis risk in communities study: 1987-1998. Diabetes care. 2002;25(8):1358-1364.

19. Chao C, Song Y, Cook N, et al. The lack of utility of circulating biomarkers of inflammation and endothelial dysfunction for type 2 diabetes risk prediction among postmenopausal women: the Women's Health Initiative Observational Study. Archives of internal medicine. 2010;170(17):1557-1565.

20. Chien KL, Chen MF, Hsu HC, et al. Plasma uric acid and the risk of type 2 diabetes in a Chinese community. Clinical chemistry. 2008;54(2):310-316.

21. Choi JH, Rhee EJ, Bae JC, et al. Increased risk of type 2 diabetes in subjects with both elevated liver enzymes and ultrasonographically diagnosed nonalcoholic fatty liver disease: a 4-year longitudinal study. Archives of medical research. 2013;44(2):115-120.

22. Choi KM, Lee J, Lee KW, et al. Serum adiponectin concentrations predict the developments of type 2 diabetes and the metabolic syndrome in elderly Koreans. Clinical endocrinology. 2004;61(1):75-80.

23. Chung CM, Lin TH, Chen JW, et al. Common quantitative trait locus downstream of RETN gene identified by genome-wide association study is associated with risk of type 2 diabetes mellitus in Han Chinese: a Mendelian randomization effect. Diabetes/metabolism research and reviews. 2014;30(3):232-240.

24. Daimon M, Oizumi T, Saitoh T, et al. Decreased serum levels of adiponectin are a risk factor for the progression to type 2 diabetes in the Japanese Population: the Funagata study. Diabetes care. 2003;26(7):2015-2020.

25. Dallmeier D, Larson MG, Wang N, Fontes JD, Benjamin EJ, Fox CS. Addition of inflammatory biomarkers did not improve diabetes prediction in the community: the framingham heart study. Journal of the American Heart Association. 2012;1(4):e000869.

26. Dankner R, Abdul-Ghani MA, Gerber Y, Chetrit A, Wainstein J, Raz I. Predicting the 20-year diabetes incidence rate. Diabetes/metabolism research and reviews. 2007;23(7):551-558.

27. Dastani Z, Hivert MF, Timpson N, et al. Novel loci for adiponectin levels and their influence on type 2 diabetes and metabolic traits: a multi-ethnic meta-analysis of 45,891 individuals. PLoS genetics. 2012;8(3):e1002607.

28. De Silva NM, Freathy RM, Palmer TM, et al. Mendelian randomization studies do not support a role for raised circulating triglyceride levels influencing type 2 diabetes, glucose levels, or insulin resistance. Diabetes. 2011;60(3):1008-1018.

29. Djousse L, Biggs ML, Lemaitre RN, et al. Plasma omega-3 fatty acids and incident diabetes in older adults. The American journal of clinical nutrition. 2011;94(2):527-533.

30. Doi Y, Kiyohara Y, Kubo M, et al. Elevated C-reactive protein is a predictor of the development of diabetes in a general Japanese population: the Hisayama Study. Diabetes care. 2005;28(10):2497-2500.

31. Doi Y, Kubo M, Yonemoto K, et al. Liver enzymes as a predictor for incident diabetes in a Japanese population: the Hisayama study. Obesity (Silver Spring, Md.). 2007;15(7):1841-1850.

32. Doi Y, Ninomiya T, Hata J, et al. Two risk score models for predicting incident Type 2 diabetes in Japan. Diabetic medicine : a journal of the British Diabetic Association. 2012;29(1):107-114.

33. Droumaguet C, Balkau B, Simon D, et al. Use of HbA1c in predicting progression to diabetes in French men and women: data from an Epidemiological Study on the Insulin Resistance Syndrome (DESIR). Diabetes care. 2006;29(7):1619-1625.

34. Engstrom G, Smith JG, Persson M, Nilsson PM, Melander O, Hedblad B. Red cell distribution width, haemoglobin A1c and incidence of diabetes mellitus. Journal of internal medicine. 2014;276(2):174-183.

35. Enhorning S, Bankir L, Bouby N, et al. Copeptin, a marker of vasopressin, in abdominal obesity, diabetes and microalbuminuria: the prospective Malmo Diet and Cancer Study cardiovascular cohort. International journal of obesity (2005). 2013;37(4):598-603.

36. Enhorning S, Wang TJ, Nilsson PM, et al. Plasma copeptin and the risk of diabetes mellitus. Circulation. 2010;121(19):2102-2108.

37. Eskesen K, Jensen MT, Galatius S, et al. Glycated haemoglobin and the risk of cardiovascular disease, diabetes and all-cause mortality in the Copenhagen City Heart Study. Journal of internal medicine. 2013;273(1):94-101.

38. Eugen-Olsen J, Andersen O, Linneberg A, et al. Circulating soluble urokinase plasminogen activator receptor predicts cancer, cardiovascular disease, diabetes and mortality in the general population. Journal of internal medicine. 2010;268(3):296-308.

39. Fagerberg B, Kellis D, Bergstrom G, Behre CJ. Adiponectin in relation to insulin sensitivity and insulin secretion in the development of type 2 diabetes: a prospective study in 64-year-old women. Journal of internal medicine. 2011;269(6):636-643.

40. Ferrannini E, Natali A, Camastra S, et al. Early metabolic markers of the development of dysglycemia and type 2 diabetes and their physiological significance. Diabetes. 2013;62(5):1730-1737.

41. Festa A, D'Agostino R, Jr., Tracy RP, Haffner SM, Insulin Resistance Atherosclerosis S. Elevated levels of acute-phase proteins and plasminogen activator inhibitor-1 predict the development of type 2 diabetes: the insulin resistance atherosclerosis study. Diabetes. 2002;51(4):1131-1137.

42. Floegel A, Stefan N, Yu Z, et al. Identification of serum metabolites associated with risk of type 2 diabetes using a targeted metabolomic approach. Diabetes. 2013;62(2):639-648.

43. Ford ES, Schulze MB, Bergmann MM, Thamer C, Joost HG, Boeing H. Liver enzymes and incident diabetes: findings from the European Prospective Investigation into Cancer and Nutrition (EPIC)-Potsdam Study. Diabetes care. 2008;31(6):1138-1143.

44. Forouhi NG, Ye Z, Rickard AP, et al. Circulating 25-hydroxyvitamin D concentration and the risk of type 2 diabetes: results from the European Prospective Investigation into Cancer (EPIC)-Norfolk cohort and updated meta-analysis of prospective studies. Diabetologia. 2012;55(8):2173-2182.

45. Fraser A, Harris R, Sattar N, Ebrahim S, Davey Smith G, Lawlor DA. Alanine aminotransferase, gamma-glutamyltransferase, and incident diabetes: the British Women's Heart and Health Study and meta-analysis. Diabetes care. 2009;32(4):741-750.

46. Gan W, Guan Y, Wu Q, et al. Association of TMPRSS6 polymorphisms with ferritin, hemoglobin, and type 2 diabetes risk in a Chinese Han population. The American journal of clinical nutrition. 2012;95(3):626-632.

47. Gao WG, Qiao Q, Pitkaniemi J, et al. Risk prediction models for the development of diabetes in Mauritian Indians. Diabetic medicine : a journal of the British Diabetic Association. 2009;26(10):996-1002.

48. Gast GC, Spijkerman AM, Van der AD, Jacobs-van der Bruggen MA, Verschuren WM. Five-year changes in biologic risk factors and risk of type 2 diabetes: are attained but not initial risk factor levels of importance? American journal of epidemiology. 2012;176(8):720-725.

49. Gautier A, Balkau B, Lange C, Tichet J, Bonnet F, Group DS. Risk factors for incident type 2 diabetes in individuals with a BMI of <27 kg/m2: the role of gamma-glutamyltransferase. Data from an Epidemiological Study on the Insulin Resistance Syndrome (DESIR). Diabetologia. 2010;53(2):247-253.

50. Goessling W, Massaro JM, Vasan RS, D'Agostino RB, Sr., Ellison RC, Fox CS. Aminotransferase levels and 20-year risk of metabolic syndrome, diabetes, and cardiovascular disease. Gastroenterology. 2008;135(6):1935-1944, 1944 e1931.

51. Grimnes G, Emaus N, Joakimsen RM, et al. Baseline serum 25-hydroxyvitamin D concentrations in the Tromso Study 1994-95 and risk of developing type 2 diabetes mellitus during 11 years of follow-up. Diabetic medicine : a journal of the British Diabetic Association. 2010;27(10):1107-1115.

52. Halimi JM, Bonnet F, Lange C, et al. Urinary albumin excretion is a risk factor for diabetes mellitus in men, independently of initial metabolic profile and development of insulin resistance. The DESIR Study. Journal of hypertension. 2008;26(11):2198-2206.

53. Handberg A, Norberg M, Stenlund H, Hallmans G, Attermann J, Eriksson JW. Soluble CD36 (sCD36) clusters with markers of insulin resistance, and high sCD36 is associated with increased type 2 diabetes risk. The Journal of clinical endocrinology and metabolism. 2010;95(4):1939-1946.

54. Harati H, Hadaegh F, Tohidi M, Azizi F. Impaired fasting glucose cutoff value of 5.6 mmol/l combined with other cardiovascular risk markers is a better predictor for incident Type 2 diabetes than the 6.1 mmol/l value: Tehran lipid and glucose study. Diabetes research and clinical practice. 2009;85(1):90-95.

55. Harita N, Hayashi T, Sato KK, et al. Lower serum creatinine is a new risk factor of type 2 diabetes: the Kansai healthcare study. Diabetes care. 2009;32(3):424-426.

56. Haugaard SB, Andersen O, Hansen TW, et al. The immune marker soluble urokinase plasminogen activator receptor is associated with new-onset diabetes in non-smoking women and men. Diabetic medicine : a journal of the British Diabetic Association. 2012;29(4):479-487.

57. Heianza Y, Arase Y, Fujihara K, et al. Screening for pre-diabetes to predict future diabetes using various cut-off points for HbA(1c) and impaired fasting glucose: the Toranomon Hospital Health Management Center Study 4 (TOPICS 4). Diabetic medicine : a journal of the British Diabetic Association. 2012;29(9):e279-285.

58. Heidemann C, Sun Q, van Dam RM, et al. Total and high-molecular-weight adiponectin and resistin in relation to the risk for type 2 diabetes in women. Annals of internal medicine. 2008;149(5):307-316.

59. Herder C, Baumert J, Zierer A, et al. Immunological and cardiometabolic risk factors in the prediction of type 2 diabetes and coronary events: MONICA/KORA Augsburg case-cohort study. PloS one. 2011;6(6):e19852.

60. Herder C, Karakas M, Koenig W. Biomarkers for the prediction of type 2 diabetes and cardiovascular disease. Clinical pharmacology and therapeutics. 2011;90(1):52-66.

61. Hernestal-Boman J, Norberg M, Jansson JH, et al. Signs of dysregulated fibrinolysis precede the development of type 2 diabetes mellitus in a population-based study. Cardiovascular diabetology. 2012;11:152.

62. Hjellvik V, Sakshaug S, Strom H. Body mass index, triglycerides, glucose, and blood pressure as predictors of type 2 diabetes in a middle-aged Norwegian cohort of men and women. Clinical epidemiology. 2012;4:213-224.

63. Hoogeveen RC, Ballantyne CM, Bang H, et al. Circulating oxidised low-density lipoprotein and intercellular adhesion molecule-1 and risk of type 2 diabetes mellitus: the Atherosclerosis Risk in Communities Study. Diabetologia. 2007;50(1):36-42.

64. Hu FB, Meigs JB, Li TY, Rifai N, Manson JE. Inflammatory markers and risk of developing type 2 diabetes in women. Diabetes. 2004;53(3):693-700.

65. Huang T, Ren J, Huang J, Li D. Association of homocysteine with type 2 diabetes: a meta-analysis implementing Mendelian randomization approach. BMC genomics. 2013;14:867.

66. Husemoen LL, Thuesen BH, Fenger M, et al. Serum 25(OH)D and type 2 diabetes association in a general population: a prospective study. Diabetes care. 2012;35(8):1695-1700.

67. Il'yasova D, Spasojevic I, Base K, et al. Urinary F2-isoprostanes as a biomarker of reduced risk of type 2 diabetes. Diabetes care. 2012;35(1):173-174.

68. Interleukin 1 Genetics C. Cardiometabolic effects of genetic upregulation of the interleukin 1 receptor antagonist: a Mendelian randomisation analysis. The lancet. Diabetes & endocrinology. 2015;3(4):243-253.

69. Ito C, Maeda R, Nakamura K, Sasaki H. Prediction of diabetes mellitus (NIDDM). Diabetes research and clinical practice. 1996;34 Suppl:S7-11.

70. Ix JH, Wassel CL, Kanaya AM, et al. Fetuin-A and incident diabetes mellitus in older persons. Jama. 2008;300(2):182-188.

71. Jensen MK, Bartz TM, Djousse L, et al. Genetically elevated fetuin-A levels, fasting glucose levels, and risk of type 2 diabetes: the cardiovascular health study. Diabetes care. 2013;36(10):3121-3127.

72. Jia Z, Zhang X, Kang S, Wu Y. Serum uric acid levels and incidence of impaired fasting glucose and type 2 diabetes mellitus: a meta-analysis of cohort studies. Diabetes research and clinical practice. 2013;101(1):88-96.

73. Jobs E, Riserus U, Ingelsson E, et al. Serum cathepsin S is associated with decreased insulin sensitivity and the development of type 2 diabetes in a community-based cohort of elderly men. Diabetes care. 2013;36(1):163-165.

74. Julia C, Czernichow S, Charnaux N, et al. Relationships between adipokines, biomarkers of endothelial function and inflammation and risk of type 2 diabetes. Diabetes research and clinical practice. 2014;105(2):231-238.

75. Juraschek SP, Shantha GP, Chu AY, et al. Lactate and risk of incident diabetes in a case-cohort of the atherosclerosis risk in communities (ARIC) study. PloS one. 2013;8(1):e55113.

76. Kanaya AM, Harris T, Goodpaster BH, et al. Adipocytokines attenuate the association between visceral adiposity and diabetes in older adults. Diabetes care. 2004;27(6):1375-1380.

77. Kashima S, Inoue K, Matsumoto M, Akimoto K. Do non-glycaemic markers add value to plasma glucose and hemoglobin a1c in predicting diabetes? Yuport health checkup center study. PloS one. 2013;8(6):e66899.

78. Kato M, Noda M, Suga H, et al. Haemoglobin A1c cut-off point to identify a high risk group of future diabetes: results from the Omiya MA Cohort Study. Diabetic medicine : a journal of the British Diabetic Association. 2012;29(7):905-910.

79. Khan H, Kunutsor S, Franco OH, Chowdhury R. Vitamin D, type 2 diabetes and other metabolic outcomes: a systematic review and meta-analysis of prospective studies. The Proceedings of the Nutrition Society. 2013;72(1):89-97.

80. Kim CH, Park JY, Lee KU, Kim JH, Kim HK. Association of serum gamma-glutamyltransferase and alanine aminotransferase activities with risk of type 2 diabetes mellitus independent of fatty liver. Diabetes/metabolism research and reviews. 2009;25(1):64-69.

81. Kim DJ, Cho NH, Noh JH, et al. Fasting plasma glucose cutoff value for the prediction of future diabetes development: a study of middle-aged Koreans in a health promotion center. Journal of Korean medical science. 2005;20(4):562-565.

82. Ko KP, Kim CS, Ahn Y, et al. Plasma isoflavone concentration is associated with decreased risk of type 2 diabetes in Korean women but not men: results from the Korean Genome and Epidemiology Study. Diabetologia. 2015;58(4):726-735.

83. Kodama S, Horikawa C, Fujihara K, et al. Use of high-normal levels of haemoglobin A(1C) and fasting plasma glucose for diabetes screening and for prediction: a meta-analysis. Diabetes/metabolism research and reviews. 2013;29(8):680-692.

84. Kodama S, Saito K, Yachi Y, et al. Association between serum uric acid and development of type 2 diabetes. Diabetes care. 2009;32(9):1737-1742.

85. Krakoff J, Funahashi T, Stehouwer CD, et al. Inflammatory markers, adiponectin, and risk of type 2 diabetes in the Pima Indian. Diabetes care. 2003;26(6):1745-1751.

86. Krishnan E, Pandya BJ, Chung L, Hariri A, Dabbous O. Hyperuricemia in young adults and risk of insulin resistance, prediabetes, and diabetes: a 15-year follow-up study. American journal of epidemiology. 2012;176(2):108-116.

87. Kunutsor SK, Abbasi A, Adler AI. Gamma-glutamyl transferase and risk of type II diabetes: an updated systematic review and dose-response meta-analysis. Annals of epidemiology. 2014;24(11):809-816.

88. Kunutsor SK, Abbasi A, Apekey TA. Aspartate aminotransferase - risk marker for type-2 diabetes mellitus or red herring? Frontiers in endocrinology. 2014;5:189.

89. Kunutsor SK, Apekey TA, Walley J. Liver aminotransferases and risk of incident type 2 diabetes: a systematic review and meta-analysis. American journal of epidemiology. 2013;178(2):159-171.

90. Kunutsor SK, Apekey TA, Walley J, Kain K. Ferritin levels and risk of type 2 diabetes mellitus: an updated systematic review and meta-analysis of prospective evidence. Diabetes/metabolism research and reviews. 2013;29(4):308-318.

91. Laaksonen DE, Niskanen L, Nyyssonen K, et al. C-reactive protein and the development of the metabolic syndrome and diabetes in middle-aged men. Diabetologia. 2004;47(8):1403-1410.

92. Lee CC, Adler AI, Sandhu MS, et al. Association of C-reactive protein with type 2 diabetes: prospective analysis and meta-analysis. Diabetologia. 2009;52(6):1040-1047.

93. Lee DH, Ha MH, Kim JH, et al. Gamma-glutamyltransferase and diabetes--a 4 year follow-up study. Diabetologia. 2003;46(3):359-364.

94. Lee DH, Jacobs DR, Jr., Gross M, et al. Gamma-glutamyltransferase is a predictor of incident diabetes and hypertension: the Coronary Artery Risk Development in Young Adults (CARDIA) Study. Clinical chemistry. 2003;49(8):1358-1366.

95. Lee SH, Kwon HS, Park YM, et al. Predicting the development of diabetes using the product of triglycerides and glucose: the Chungju Metabolic Disease Cohort (CMC) study. PloS one. 2014;9(2):e90430.

96. Ley SH, Harris SB, Connelly PW, et al. Adipokines and incident type 2 diabetes in an Aboriginal Canadian [corrected] population: the Sandy Lake Health and Diabetes Project. Diabetes care. 2008;31(7):1410-1415.

97. Ley SH, Harris SB, Connelly PW, et al. Association of apolipoprotein B with incident type 2 diabetes in an aboriginal Canadian population. Clinical chemistry. 2010;56(4):666-670.

98. Li S, Shin HJ, Ding EL, van Dam RM. Adiponectin levels and risk of type 2 diabetes: a systematic review and meta-analysis. Jama. 2009;302(2):179-188.

99. Lim NK, Park SH, Choi SJ, Lee KS, Park HY. A risk score for predicting the incidence of type 2 diabetes in a middle-aged Korean cohort: the Korean genome and epidemiology study. Circulation journal : official journal of the Japanese Circulation Society. 2012;76(8):1904-1910.

100. Lindsay RS, Krakoff J, Hanson RL, Bennett PH, Knowler WC. Gamma globulin levels predict type 2 diabetes in the Pima Indian population. Diabetes. 2001;50(7):1598-1603.

101. Liu S, Tinker L, Song Y, et al. A prospective study of inflammatory cytokines and diabetes mellitus in a multiethnic cohort of postmenopausal women. Archives of internal medicine. 2007;167(15):1676-1685.

102. Lorenzo C, Okoloise M, Williams K, Stern MP, Haffner SM, San Antonio Heart S. The metabolic syndrome as predictor of type 2 diabetes: the San Antonio heart study. Diabetes care. 2003;26(11):3153-3159.

103. Luft VC, Schmidt MI, Pankow JS, et al. Dipeptidyl peptidase IV and incident diabetes: the Atherosclerosis Risk in Communities (ARIC) study. Diabetes care. 2010;33(5):1109-1111.

104. Lv Q, Meng XF, He FF, et al. High serum uric acid and increased risk of type 2 diabetes: a systemic review and meta-analysis of prospective cohort studies. PloS one. 2013;8(2):e56864.

105. Lyssenko V, Jorgensen T, Gerwien RW, et al. Validation of a multi-marker model for the prediction of incident type 2 diabetes mellitus: combined results of the Inter99 and Botnia studies. Diabetes & vascular disease research. 2012;9(1):59-67.

106. Ma W, Wu JH, Wang Q, et al. Prospective association of fatty acids in the de novo lipogenesis pathway with risk of type 2 diabetes: the Cardiovascular Health Study. The American journal of clinical nutrition. 2015;101(1):153-163.

107. Mahendran Y, Agren J, Uusitupa M, et al. Association of erythrocyte membrane fatty acids with changes in glycemia and risk of type 2 diabetes. The American journal of clinical nutrition. 2014;99(1):79-85.

108. Mainous AG, 3rd, King DE, Pearson WS, Garr DR. Is an elevated serum transferrin saturation associated with the development of diabetes? The Journal of family practice. 2002;51(11):933-936.

109. Mandel EI, Curhan GC, Hu FB, Taylor EN. Plasma bicarbonate and risk of type 2 diabetes mellitus. CMAJ : Canadian Medical Association journal = journal de l'Association medicale canadienne. 2012;184(13):E719-725.

110. Marques-Vidal P, Schmid R, Bochud M, et al. Adipocytokines, hepatic and inflammatory biomarkers and incidence of type 2 diabetes. the CoLaus study. PloS one. 2012;7(12):e51768.

111. Mattila C, Knekt P, Mannisto S, et al. Serum 25-hydroxyvitamin D concentration and subsequent risk of type 2 diabetes. Diabetes care. 2007;30(10):2569-2570.

112. McMullan CJ, Schernhammer ES, Rimm EB, Hu FB, Forman JP. Melatonin secretion and the incidence of type 2 diabetes. Jama. 2013;309(13):1388-1396.

113. Meigs JB, Hu FB, Rifai N, Manson JE. Biomarkers of endothelial dysfunction and risk of type 2 diabetes mellitus. Jama. 2004;291(16):1978-1986.

114. Moller HJ, Frikke-Schmidt R, Moestrup SK, Nordestgaard BG, Tybjaerg-Hansen A. Serum soluble CD163 predicts risk of type 2 diabetes in the general population. Clinical chemistry. 2011;57(2):291-297.

115. Montonen J, Boeing H, Steffen A, et al. Body iron stores and risk of type 2 diabetes: results from the European Prospective Investigation into Cancer and Nutrition (EPIC)-Potsdam study. Diabetologia. 2012;55(10):2613-2621.

116. Montonen J, Drogan D, Joost HG, et al. Estimation of the contribution of biomarkers of different metabolic pathways to risk of type 2 diabetes. European journal of epidemiology. 2011;26(1):29-38.

117. Mozaffarian D, Cao H, King IB, et al. Trans-palmitoleic acid, metabolic risk factors, and new-onset diabetes in U.S. adults: a cohort study. Annals of internal medicine. 2010;153(12):790-799.

118. Mozaffarian D, Cao H, King IB, et al. Circulating palmitoleic acid and risk of metabolic abnormalities and new-onset diabetes. The American journal of clinical nutrition. 2010;92(6):1350-1358.

119. Mozaffarian D, de Oliveira Otto MC, Lemaitre RN, et al. trans-Palmitoleic acid, other dairy fat biomarkers, and incident diabetes: the Multi-Ethnic Study of Atherosclerosis (MESA). The American journal of clinical nutrition. 2013;97(4):854-861.

120. Mukai N, Doi Y, Ninomiya T, et al. Cut-off values of fasting and post-load plasma glucose and HbA1c for predicting Type 2 diabetes in community-dwelling Japanese subjects: the Hisayama Study. Diabetic medicine : a journal of the British Diabetic Association. 2012;29(1):99-106.

121. Nan H, Qiao Q, Soderberg S, et al. Serum uric acid and incident diabetes in Mauritian Indian and Creole populations. Diabetes research and clinical practice. 2008;80(2):321-327.

122. Neeland IJ, Turer AT, Ayers CR, et al. Dysfunctional adiposity and the risk of prediabetes and type 2 diabetes in obese adults. Jama. 2012;308(11):1150-1159.

123. Ngarmukos C, Chailurkit LO, Chanprasertyothin S, Hengprasith B, Sritara P, Ongphiphadhanakul B. A reduced serum level of total osteocalcin in men predicts the development of diabetes in a long-term follow-up cohort. Clinical endocrinology. 2012;77(1):42-46.

124. Nguyen QM, Srinivasan SR, Xu JH, Chen W, Berenson GS. Fasting plasma glucose levels within the normoglycemic range in childhood as a predictor of prediabetes and type 2 diabetes in adulthood: the Bogalusa Heart Study. Archives of pediatrics & adolescent medicine. 2010;164(2):124-128.

125. Nguyen QM, Srinivasan SR, Xu JH, et al. Elevated liver function enzymes are related to the development of prediabetes and type 2 diabetes in younger adults: the Bogalusa Heart Study. Diabetes care. 2011;34(12):2603-2607.

126. Nguyen QM, Srinivasan SR, Xu JH, Chen W, Kieltyka L, Berenson GS. Utility of childhood glucose homeostasis variables in predicting adult diabetes and related cardiometabolic risk factors: the Bogalusa Heart Study. Diabetes care. 2010;33(3):670-675.

127. Nilsson SE, Fransson E, Brismar K. Relationship between serum progesterone concentrations and cardiovascular disease, diabetes, and mortality in elderly Swedish men and women: An 8-year prospective study. Gender medicine. 2009;6(3):433-443.

128. Noda M, Kato M, Takahashi Y, et al. Fasting plasma glucose and 5-year incidence of diabetes in the JPHC diabetes study - suggestion for the threshold for impaired fasting glucose among Japanese. Endocrine journal. 2010;57(7):629-637.

129. Nomura K, Inoue K, Akimoto K. A two-step screening, measurement of HbA1c in association with FPG, may be useful in predicting diabetes. PloS one. 2012;7(4):e36309.

130. Norberg M, Eriksson JW, Lindahl B, et al. A combination of HbA1c, fasting glucose and BMI is effective in screening for individuals at risk of future type 2 diabetes: OGTT is not needed. Journal of internal medicine. 2006;260(3):263-271.

131. Norberg M, Stenlund H, Lindahl B, et al. Components of metabolic syndrome predicting diabetes: no role of inflammation or dyslipidemia. Obesity (Silver Spring, Md.). 2007;15(7):1875-1885.

132. Oda E, Aizawa Y. Metabolic syndrome is a poor predictor of diabetes in a Japanese health screening population. Internal medicine (Tokyo, Japan). 2013;52(7):721-725.

133. Onat A, Can G, Hergenc G, Yazici M, Karabulut A, Albayrak S. Serum apolipoprotein B predicts dyslipidemia, metabolic syndrome and, in women, hypertension and diabetes, independent of markers of central obesity and inflammation. International journal of obesity (2005). 2007;31(7):1119-1125.

134. Onat A, Can G, Yuksel H, Ayhan E, Dogan Y, Hergenc G. An algorithm to predict risk of type 2 diabetes in Turkish adults: contribution of C-reactive protein. Journal of endocrinological investigation. 2011;34(8):580-586.

135. Onat A, Hergenc G, Ayhan E, Ugur M, Can G. Impaired anti-inflammatory function of apolipoprotein A-II concentrations predicts metabolic syndrome and diabetes at 4 years follow-up in elderly Turks. Clinical chemistry and laboratory medicine : CCLM / FESCC. 2009;47(11):1389-1394.

136. Orban E, Schwab S, Thorand B, Huth C. Association of iron indices and type 2 diabetes: a meta-analysis of observational studies. Diabetes/metabolism research and reviews. 2014;30(5):372-394.

137. Parker J, Hashmi O, Dutton D, et al. Levels of vitamin D and cardiometabolic disorders: systematic review and meta-analysis. Maturitas. 2010;65(3):225-236.

138. Patel PS, Cooper AJ, O'Connell TC, et al. Serum carbon and nitrogen stable isotopes as potential biomarkers of dietary intake and their relation with incident type 2 diabetes: the EPIC-Norfolk study. The American journal of clinical nutrition. 2014;100(2):708-718.

139. Pfister R, Barnes D, Luben R, et al. No evidence for a causal link between uric acid and type 2 diabetes: a Mendelian randomisation approach. Diabetologia. 2011;54(10):2561-2569.

140. Pfister R, Sharp S, Luben R, et al. Mendelian randomization study of B-type natriuretic peptide and type 2 diabetes: evidence of causal association from population studies. PLoS medicine. 2011;8(10):e1001112.

141. Pradhan AD, Manson JE, Meigs JB, et al. Insulin, proinsulin, proinsulin:insulin ratio, and the risk of developing type 2 diabetes mellitus in women. The American journal of medicine. 2003;114(6):438-444.

142. Pradhan AD, Manson JE, Rifai N, Buring JE, Ridker PM. C-reactive protein, interleukin 6, and risk of developing type 2 diabetes mellitus. Jama. 2001;286(3):327-334.

143. Pradhan AD, Rifai N, Buring JE, Ridker PM. Hemoglobin A1c predicts diabetes but not cardiovascular disease in nondiabetic women. The American journal of medicine. 2007;120(8):720-727.

144. Ramachandran A, Snehalatha C, Samith Shetty A, Nanditha A. Predictive value of HbA1c for incident diabetes among subjects with impaired glucose tolerance--analysis of the Indian Diabetes Prevention Programmes. Diabetic medicine : a journal of the British Diabetic Association. 2012;29(1):94-98.

145. Rathmann W, Kowall B, Heier M, et al. Prediction models for incident type 2 diabetes mellitusin the older population: KORA S4/F4 cohort study. Diabetic medicine : a journal of the British Diabetic Association. 2010;27(10):1116-1123.

146. Raynor LA, Pankow JS, Duncan BB, et al. Novel risk factors and the prediction of type 2 diabetes in the Atherosclerosis Risk in Communities (ARIC) study. Diabetes care. 2013;36(1):70-76.

147. Rhee EJ, Seo MH, Jeon WS, et al. The association of baseline adipocytokine levels with glycemic progression in nondiabetic Korean adults in 4 years of follow-up. Diabetes research and clinical practice. 2012;98(3):501-507.

148. Rhee EP, Cheng S, Larson MG, et al. Lipid profiling identifies a triacylglycerol signature of insulin resistance and improves diabetes prediction in humans. The Journal of clinical investigation. 2011;121(4):1402-1411.

149. Rolandsson O, Hagg E, Nilsson M, Hallmans G, Mincheva-Nilsson L, Lernmark A. Prediction of diabetes with body mass index, oral glucose tolerance test and islet cell autoantibodies in a regional population. Journal of internal medicine. 2001;249(4):279-288.

150. Sahakyan K, Lee KE, Shankar A, Klein R. Serum cystatin C and the incidence of type 2 diabetes mellitus. Diabetologia. 2011;54(6):1335-1340.

151. Salomaa V, Havulinna A, Saarela O, et al. Thirty-one novel biomarkers as predictors for clinically incident diabetes. PloS one. 2010;5(4):e10100.

152. Santaren ID, Watkins SM, Liese AD, et al. Serum pentadecanoic acid (15:0), a short-term marker of dairy food intake, is inversely associated with incident type 2 diabetes and its underlying disorders. The American journal of clinical nutrition. 2014;100(6):1532-1540.

153. Sattar N, Murray HM, Welsh P, et al. Are elevated circulating intercellular adhesion molecule 1 levels more strongly predictive of diabetes than vascular risk? Outcome of a prospective study in the elderly. Diabetologia. 2009;52(2):235-239.

154. Schafer AL, Napoli N, Lui L, Schwartz AV, Black DM, Study of Osteoporotic F. Serum 25-hydroxyvitamin D concentration does not independently predict incident diabetes in older women. Diabetic medicine : a journal of the British Diabetic Association. 2014;31(5):564-569.

155. Schmidt MI, Duncan BB, Bang H, et al. Identifying individuals at high risk for diabetes: The Atherosclerosis Risk in Communities study. Diabetes care. 2005;28(8):2013-2018.

156. Schottker B, Herder C, Rothenbacher D, Perna L, Muller H, Brenner H. Serum 25-hydroxyvitamin D levels and incident diabetes mellitus type 2: a competing risk analysis in a large population-based cohort of older adults. European journal of epidemiology. 2013;28(3):267-275.

157. Schottker B, Raum E, Rothenbacher D, Muller H, Brenner H. Prognostic value of haemoglobin A1c and fasting plasma glucose for incident diabetes and implications for screening. European journal of epidemiology. 2011;26(10):779-787.

158. Schulze MB, Solomon CG, Rifai N, et al. Hyperproinsulinaemia and risk of Type 2 diabetes mellitus in women. Diabetic medicine : a journal of the British Diabetic Association. 2005;22(9):1178-1184.

159. Schulze MB, Weikert C, Pischon T, et al. Use of multiple metabolic and genetic markers to improve the prediction of type 2 diabetes: the EPIC-Potsdam Study. Diabetes care. 2009;32(11):2116-2119.

160. Selvin E, Rawlings AM, Grams M, et al. Fructosamine and glycated albumin for risk stratification and prediction of incident diabetes and microvascular complications: a prospective cohort analysis of the Atherosclerosis Risk in Communities (ARIC) study. The lancet. Diabetes & endocrinology. 2014;2(4):279-288.

161. Shlomai A, Kariv R, Leshno M, Beth-or A, Sheinberg B, Halpern Z. Large-scale population analysis reveals an extremely low threshold for "non-healthy" alanine aminotransferase that predicts diabetes mellitus. Journal of gastroenterology and hepatology. 2010;25(10):1687-1691.

162. Sluijs I, Beulens JW, van der AD, Spijkerman AM, Schulze MB, van der Schouw YT. Plasma uric acid is associated with increased risk of type 2 diabetes independent of diet and metabolic risk factors. The Journal of nutrition. 2013;143(1):80-85.

163. Song Y, Manson JE, Tinker L, et al. Insulin sensitivity and insulin secretion determined by homeostasis model assessment and risk of diabetes in a multiethnic cohort of women: the Women's Health Initiative Observational Study. Diabetes care. 2007;30(7):1747-1752.

164. Song Y, Manson JE, Tinker L, et al. Circulating levels of endothelial adhesion molecules and risk of diabetes in an ethnically diverse cohort of women. Diabetes. 2007;56(7):1898-1904.

165. Song Y, Wang L, Pittas AG, et al. Blood 25-hydroxy vitamin D levels and incident type 2 diabetes: a meta-analysis of prospective studies. Diabetes care. 2013;36(5):1422-1428.

166. Song Y, Yeung E, Liu A, et al. Pancreatic beta-cell function and type 2 diabetes risk: quantify the causal effect using a Mendelian randomization approach based on meta-analyses. Human molecular genetics. 2012;21(22):5010-5018.

167. Soulimane S, Simon D, Shaw J, et al. HbA1c, fasting plasma glucose and the prediction of diabetes: Inter99, AusDiab and D.E.S.I.R. Diabetes research and clinical practice. 2012;96(3):392-399.

168. Stefan N, Fritsche A, Weikert C, et al. Plasma fetuin-A levels and the risk of type 2 diabetes. Diabetes. 2008;57(10):2762-2767.

169. Stefan N, Sun Q, Fritsche A, et al. Impact of the adipokine adiponectin and the hepatokine fetuin-A on the development of type 2 diabetes: prospective cohort- and cross-sectional phenotyping studies. PloS one. 2014;9(3):e92238.

170. Steffen BT, Steffen LM, Zhou X, Ouyang P, Weir NL, Tsai MY. n-3 Fatty acids attenuate the risk of diabetes associated with elevated serum nonesterified fatty acids: the multi-ethnic study of atherosclerosis. Diabetes care. 2015;38(4):575-580.

171. Stranges S, Rafalson LB, Dmochowski J, et al. Additional contribution of emerging risk factors to the prediction of the risk of type 2 diabetes: evidence from the Western New York Study. Obesity (Silver Spring, Md.). 2008;16(6):1370-1376.

172. Sun L, Zong G, Pan A, et al. Elevated plasma ferritin is associated with increased incidence of type 2 diabetes in middle-aged and elderly Chinese adults. The Journal of nutrition. 2013;143(9):1459-1465.

173. Sun Q, van Dam RM, Meigs JB, Franco OH, Mantzoros CS, Hu FB. Leptin and soluble leptin receptor levels in plasma and risk of type 2 diabetes in U.S. women: a prospective study. Diabetes. 2010;59(3):611-618.

174. Tabak AG, Jokela M, Akbaraly TN, Brunner EJ, Kivimaki M, Witte DR. Trajectories of glycaemia, insulin sensitivity, and insulin secretion before diagnosis of type 2 diabetes: an analysis from the Whitehall II study. Lancet (London, England). 2009;373(9682):2215-2221.

175. Thorand B, Baumert J, Chambless L, et al. Elevated markers of endothelial dysfunction predict type 2 diabetes mellitus in middle-aged men and women from the general population. Arteriosclerosis, thrombosis, and vascular biology. 2006;26(2):398-405.

176. Thorand B, Baumert J, Herder C, Meisinger C, Koenig W. Soluble thrombomodulin as a predictor of type 2 diabetes: results from the MONICA/KORA Augsburg case-cohort study, 1984-1998. Diabetologia. 2007;50(3):545-548.

177. Thorand B, Baumert J, Kolb H, et al. Sex differences in the prediction of type 2 diabetes by inflammatory markers: results from the MONICA/KORA Augsburg case-cohort study, 1984-2002. Diabetes care. 2007;30(4):854-860.

178. Thorand B, Kolb H, Baumert J, et al. Elevated levels of interleukin-18 predict the development of type 2 diabetes: results from the MONICA/KORA Augsburg Study, 1984-2002. Diabetes. 2005;54(10):2932-2938.

179. Thorand B, Lowel H, Schneider A, et al. C-reactive protein as a predictor for incident diabetes mellitus among middle-aged men: results from the MONICA Augsburg cohort study, 1984-1998. Archives of internal medicine. 2003;163(1):93-99.

180. Thorand B, Zierer A, Baumert J, Meisinger C, Herder C, Koenig W. Associations between leptin and the leptin / adiponectin ratio and incident Type 2 diabetes in middle-aged men and women: results from the MONICA / KORA Augsburg study 1984-2002. Diabetic medicine : a journal of the British Diabetic Association. 2010;27(9):1004-1011.

181. Trombetta M, Bonetti S, Boselli ML, et al. PPARG2 Pro12Ala and ADAMTS9 rs4607103 as "insulin resistance loci" and "insulin secretion loci" in Italian individuals. The GENFIEV study and the Verona Newly Diagnosed Type 2 Diabetes Study (VNDS) 4. Acta diabetologica. 2013;50(3):401-408.

182. Urdea M, Kolberg J, Wilber J, et al. Validation of a multimarker model for assessing risk of type 2 diabetes from a five-year prospective study of 6784 Danish people (Inter99). Journal of diabetes science and technology. 2009;3(4):748-755.

183. Vaccaro O, Cuomo V, Trevisan M, et al. Enhanced Na-Li countertransport: a marker of inherited susceptibility to type 2 diabetes. International journal of epidemiology. 2005;34(5):1123-1128.

184. Valdes S, Botas P, Delgado E, Alvarez F, Cadorniga FD. Does the new American Diabetes Association definition for impaired fasting glucose improve its ability to predict type 2 diabetes mellitus in Spanish persons? The Asturias Study. Metabolism: clinical and experimental. 2008;57(3):399-403.

185. Vigo A, Duncan BB, Schmidt MI, et al. Glutamic acid decarboxylase antibodies are indicators of the course, but not of the onset, of diabetes in middle-aged adults: the Atherosclerosis Risk in Communities Study. Brazilian journal of medical and biological research = Revista brasileira de pesquisas medicas e biologicas / Sociedade Brasileira de Biofisica ... [et al.]. 2007;40(7):933-941.

186. Vozarova B, Stefan N, Lindsay RS, et al. High alanine aminotransferase is associated with decreased hepatic insulin sensitivity and predicts the development of type 2 diabetes. Diabetes. 2002;51(6):1889-1895.

187. Vozarova B, Weyer C, Lindsay RS, Pratley RE, Bogardus C, Tataranni PA. High white blood cell count is associated with a worsening of insulin sensitivity and predicts the development of type 2 diabetes. Diabetes. 2002;51(2):455-461.

188. Wahid ST, Sultan J, Handley G, Saeed BO, Weaver JU, Robinson AC. Serum fructosamine as a marker of 5-year risk of developing diabetes mellitus in patients exhibiting stress hyperglycaemia. Diabetic medicine : a journal of the British Diabetic Association. 2002;19(7):543-548.

189. Wang T, Bi Y, Xu M, et al. Serum uric acid associates with the incidence of type 2 diabetes in a prospective cohort of middle-aged and elderly Chinese. Endocrine. 2011;40(1):109-116.

190. Wang TJ, Larson MG, Vasan RS, et al. Metabolite profiles and the risk of developing diabetes. Nature medicine. 2011;17(4):448-453.

191. Wang TJ, Ngo D, Psychogios N, et al. 2-Aminoadipic acid is a biomarker for diabetes risk. The Journal of clinical investigation. 2013;123(10):4309-4317.

192. Wang X, Bao W, Liu J, et al. Inflammatory markers and risk of type 2 diabetes: a systematic review and meta-analysis. Diabetes care. 2013;36(1):166-175.

193. Wang Z, Hoy WE. Albuminuria as a marker of the risk of developing type 2 diabetes in non-diabetic Aboriginal Australians. International journal of epidemiology. 2006;35(5):1331-1335.

194. Wang-Sattler R, Yu Z, Herder C, et al. Novel biomarkers for pre-diabetes identified by metabolomics. Molecular systems biology. 2012;8:615.

195. Wannamethee SG, Papacosta O, Whincup PH, et al. The potential for a two-stage diabetes risk algorithm combining non-laboratory-based scores with subsequent routine non-fasting blood tests: results from prospective studies in older men and women. Diabetic medicine : a journal of the British Diabetic Association. 2011;28(1):23-30.

196. Wannamethee SG, Sattar N, Rumley A, Whincup PH, Lennon L, Lowe GD. Tissue plasminogen activator, von Willebrand factor, and risk of type 2 diabetes in older men. Diabetes care. 2008;31(5):995-1000.

197. Warren LL, Li L, Nelson MR, et al. Deep resequencing unveils genetic architecture of ADIPOQ and identifies a novel low-frequency variant strongly associated with adiponectin variation. Diabetes. 2012;61(5):1297-1301.

198. Welsh P, Murray HM, Buckley BM, et al. Leptin predicts diabetes but not cardiovascular disease: results from a large prospective study in an elderly population. Diabetes care. 2009;32(2):308-310.

199. Woo YC, Tso AW, Xu A, et al. Combined use of serum adiponectin and tumor necrosis factor-alpha receptor 2 levels was comparable to 2-hour post-load glucose in diabetes prediction. PloS one. 2012;7(5):e36868.

200. Wu JH, Micha R, Imamura F, et al. Omega-3 fatty acids and incident type 2 diabetes: a systematic review and meta-analysis. The British journal of nutrition. 2012;107 Suppl 2:S214-227.

201. Xu Y, Xu M, Huang Y, et al. Elevated serum gamma-glutamyltransferase predicts the development of impaired glucose metabolism in middle-aged and elderly Chinese. Endocrine. 2011;40(2):265-272.

202. Yaghootkar H, Lamina C, Scott RA, et al. Mendelian randomization studies do not support a causal role for reduced circulating adiponectin levels in insulin resistance and type 2 diabetes. Diabetes. 2013;62(10):3589-3598.

203. Ye Z, Sharp SJ, Burgess S, et al. Association between circulating 25-hydroxyvitamin D and incident type 2 diabetes: a mendelian randomisation study. The lancet. Diabetes & endocrinology. 2015;3(1):35-42.

204. Zhao Z, Li S, Liu G, et al. Body iron stores and heme-iron intake in relation to risk of type 2 diabetes: a systematic review and meta-analysis. PloS one. 2012;7(7):e41641.

205. Zheng T, Gao Y, Baskota A, Chen T, Ran X, Tian H. Increased plasma DPP4 activity is predictive of prediabetes and type 2 diabetes onset in Chinese over a four-year period: result from the China National Diabetes and Metabolic Disorders Study. The Journal of clinical endocrinology and metabolism. 2014;99(11):E2330-2334.
