# Supplementary material for: A Systematic Review of Biomarkers and Risk of Incident Type 2 Diabetes: An Overview of Epidemiological, Prediction and Aetiological Research Literature
Source: PLoS One. 2016 Oct 27;11(10):e0163721. doi: 10.1371/journal.pone.0163721 (PMC5082867; doi:10.1371/journal.pone.0163721)
Supplement: S1 Table — (DOC) [file pone.0163721.s005.doc]

**S1 Table. General Characteristics of 139 Studies**

| **Study** | | **year** | | **Country** | | **Design** | | **Cases/total** | | **Diabetes definition*** | | **Follow-up years (median or mean)** | | **Age years (mean or range)** | | **Male (%)** | | **Family history of DM(%)** | | **BMI (mean, median or range)** | | **Smoking (%)** | | **Glucose levels (mean or median)** | | | **HbA1c (mean±sd)** | |
| --- | --- | --- | --- | --- | --- | --- | --- | --- | --- | --- | --- | --- | --- | --- | --- | --- | --- | --- | --- | --- | --- | --- | --- | --- | --- | --- | --- | --- |
| Abbasi et al, EPIC-NL | 2012 | | Netherlands | | Cohort/  case-cohort | | 924/38,379 (2506 subcohort) | | 1, 3, 4, 5 | | 10.2 | | 20-70 | | 25.7 | | 19.2 | | 25.6±4.0 | | 30.6 | | 4.9±1.2 | | | 5.39±0.58 | |  |
| Abbasi,et al PREVEND | 2012 | | Netherlands | | Cohort | | F:208, M:288/F:4063, M:3909 | | 1, 2, 3 | | 7.7 | | 28-74 | | 49.3 | | F:20.4/M:19.3 | | F: 25.8±4.6/M: 26.2±3.6 | | F: 33.8/M: 35.0 | | F:4.62±0.64/M: 4.86±0.66 | | | NR | |  |
| Abbasi et al, EPIC-NL, PREVEND | 2012 | | Netherlands | | Case-cohort/  cohort | | 924/38,379 (2506 subcohort); 503/7,952 | | 1,2, 3, 4, 5 | | 10.2; 7.5 | | 20-70; 28-74 | | 25.7; 49.3 | | 19.2; 15.7 | | 25.6±4.0; 26.4±4.2 | | 30.6; 34.4 | | 4.9±1.2; 4.7±0.6 | | | 5.39±0.58; NR | |  |
| Abbasi,et al PREVEND | 2011 | | Netherlands | | Cohort | | 385/6,618 | | 1, 2, 3 | | 7.6 | | 28-74 | | 47.8 | | NR | | 25.9±4.1 | | 33.9 | | 4.7±0.6 | | | NR | |  |
| Alessi, et al DESIR | 2011 | | France | | nested case-control | | 182/363 | | 2,5 | | 9 | | 30-65 | | NR | | NR | | 28.4±44 (T2D) vs 24.8±3.1 (non-case) | | NR | | 6.1±0.6 (T2D) vs 5.3±0.5 (non-case) | | | NR | |  |
| André, et al DESIR | 2005 | | France | | Cohort | | 89/4,201 | | 2,5 | | 3 | | 30-65 | | 50.7 | | NR | | F: 23.9±3.9/M:25.3±3.1 | | NR | | F:5.05±0.5/M: 5.44±0.55 | | | NR | |  |
| Becerra-Tomàs et al, PREIMED | 2014 | | Spain | | Cohort | | 77/650 | | 2,3 | | 4.8 | | 67 | | 40 | | NR | | 29.5 | | 15 | | 95.8 mg/dl | | | NR | |  |
| Bobbert T, et al MeSyBePo | 2013 | | Germany | | Cohort | | 3/374 | | 2 | | 5.3 | | 19-75 | | 34.4 | | NR | | 26.7 (T2D) vs 31.6 (non-case) | | NR | | 89.8 (61-125) (T2D) vs 100.5 (72-124 (non-case) | | | 5.4 (3.9-11.3) (T2D) vs 5.6 (5.0-9.8) (non-case) | |  |
| Brantsma AH, et al PREVEND | 2005 | | Netherlands | | Cohort | | 185/5,654 | | 1, 2, 3 | | 4.2 | | 28-74 | | 54.1 | | 31.4 | | 29.6±4.7 (T2D) vs 25.8±4 (non-case) | | NR | | 5.7±0.7 (T2D) vs 4.7±0.6 (non-case) | | | NR | |  |
| Brunner EJ, et al Whitehall II | 2008 | | UK | | Cohort | | 354/5,274 | | 1, 2, 3 | | 12 | | 50-74 | | 73 | | NR | | 26.7±4.3 | | 8.1 | | NR | | | 5.3±0.6 | |  |
| Carnethon MR, et al ARIC | 2002 | | US | | Cohort | | H48F:750, M:729/F:7325, M:5962 | | 1,2 | | 8.7 | | 45-64 | | 44.9 | | NR | | F-B:30.2±6.5/F-W:26.2±5.2 M-B:27.2±4.7 M-W:27.2±3.9 | | F:25/ M: 27.2 | | F-B:98.2±10.1/F-W:96.7±8.7 M-B:99.4±10.1 M-W:100.8±8.9 mg/l | | | NR | |  |
| Chao C, et al WHIOS | 2010 | | US | | nested case-control | | 1,584/2,198 (80,509 cohort) | | 1,5 | | 5.9 | | 50-79 | | 0 | | 29.3 | | 26.9±5.6 | | 6 | | 122 (106-147) (T2D) vs 92 (87-98) (non-case) | | | NR | |  |
| Chien KL, et al CSCCHS | 2008 | | China | | Cohort | | 548/2690 | | 2,5 | | 9 | | 35-97 | | 47.3 | | 8.6 | | 23.29 | | 31 | | NR | | | NR | |  |
| Choi JH, et al | 2013 | | Korea | | Cohort | | 435/7,849 | | 2 | | 4 | | 44.8±5.8 | | 68.9 | | NR | | 23.7±2.8 | | 22 | | 93.7±8 (control) to 98.2±8.9 (subjects with↑ALT and steatosis) | | | 5.35±0.31 (control) to 5.47±0.35 (subjects with↑ALT and steatosis) | |  |
| Choi KM, et al SWS | 2004 | | Korea | | Cohort | | 78/372 | | 2 | | 3 | | 69.9±4.9 | | NR | | NR | | 24.8±3.1 | | NR | | 5.56±1.23 | | | NR | |  |
| Daimon M, et al Funagata | 2003 | | Japan | | Cohort | | 18/837 (normal glucose torelance) | | 3 | | 5 | | 57.4±12.4 | | 74.2 | | NR | | 23.5±3.2 | | NR | | 92±9.2 | | | 5.33±3.6 | |  |
| Dallmeier D, et al FHS | 2012 | | US | | Cohort | | 162/2,638 | | 1,2,3 | | 6.6 | | 59±9 | | 44 | | NR | | 27.7±5.1 | | 12 | | 97±10 | | | NR | |  |
| Djousse´ L, et al CHS | 2011 | | US | | Cohort | | 204/3,088 | | 2,5 | | 10.6 | | 65-98 | | 38.9 | | NR | | 26±4 | | 9.3 | | 97±10 | | | NR | |  |
| Doi Y, et al Hisayama | 2005 | | Japan | | Cohort | | 131/1,759 | | 2,5 | | 9 | | 40-79 | | 39.4 | | F:7.3/M:9.3 | | F:23.0±3.1/M: 22.9±2.9 | | F:5.4/M:47.6 | | F:5.5±0.5/ M:5.6±0.5 | | | F:5.4±0.5/ M:5.5±0.5 | |  |
| Doi Y, et al Hisayama | 2007 | | Japan | | Cohort | | 135/1,804 | | 2,5 | | 9 | | 40-79 | | 39.4 | | F:7.3/M:9.3 | | F:23.0±3.1/M: 22.9±2.9 | | F:5.4/M:47.6 | | F:5.5±0.5/ M:5.6±0.5 | | | F:5.4±0.5/ M:5.5±0.5 | |  |
| [Enhörning S, et al MDC](http://www.ncbi.nlm.nih.gov/pubmed?term=Enhörning S%5BAuthor%5D&cauthor=true&cauthor_uid=22614056) | 2013 | | Sweden | | Cohort | | 533/2,064 | | 1,2,3 | | 15.8 | | 57±5.7 | | 40.1 | | NR | | 25.6±3.7 | | NR | | 4.9 (4.6-5.2) | | | NR | |  |
| Engström G et al. Malmo | 2014 | | Sweden | | Cohort | | 2944/26709 | | 3,4,5 | | 14 | | 45-73 | | 38.9 | | NR | | 25.6 | | 28.4 | | 4.9±0.45 mmol/l | | | 4.8±0.4 % | |  |
| Eugen-Olsen, et al MONICA10 | 2010 | | Denmark | | Cohort | | 59/2,2360 | | 3,4 | | 10 | | 41-71 | | 50.3 | | NR | | 25.3 (20-34) | | 42 | | 4.7 (4-5.6) | | | NR | |  |
| Fagerberg, et al | 2011 | | Sweden | | Cohort | | 69/341 | | 2 | | 5.5 | | 64 | | 0 | | 42 (T2D) vs 24 (non-case) | | 29.0±5.3 (T2D) vs 26.5±3.4 (non-case) | | 17.3 | | 5.26±0.53 (T2D) vs 4.82±0.49 (non-case) | | | 4.8±0.3 (T2D) vs 4.5±0.3 (non-case) | |  |
| Ferrannini E, et al Botnia | 2013 | | Finland | | Cohort | | 151/2,580 | | 2,5 | | 9.5 | | 45 | | 48.9 | | 83 | | 25.8 | | NR | | 5.54 | | | NR | |  |
| Festa A, et al IRAS | 2002 | | US | | Cohort | | 144/1,047 | | 2 | | 5 | | 40-69 | | 67.7 | | NR | | 31.1±6.3 (T2D) vs 28.8±5.5 (non-case) | | NR | | 106.6±12 (T2D) vs 97.5±10.8 (non-case) | | | NR | |  |
| Floegel A, et al EPIC-Potsdam | 2013 | | Germany | | Case-cohort/ cohort | | 800/27,548 (2,282 subcohort) | | 1,3 | | 7 | | 35-65 | | 38 | | NR | | 26.1 | | 20.1 | | 88.1 (subcohort) vs 107 (T2D) | | | 5.42 (subcohort) vs 6.3 (T2D) | |  |
| Ford ES, et al EPIC-Potsdam | 2008 | | Germany | | Case-cohort/ cohort | | 787/27,548 (2,224 subcohort) | | 1,3 | | 7 | | 35-65 | | 38 | | NR | | 26±4.2 | | 20.1 | | 88±20.4 | | | NR | |  |
| Gast GS, et al Dutch Doetinchem | 2012 | | Netherlands | | Cohort | | 130/4,204 | | 1 | | 5 | | 20-59 | | 48.1 | | 18.6 | | 24.4±3.3 | | 31.8 | | 5.2±0.9 | | | NR | |  |
| Gautier A, et al DESIR | 2010 | | France | | Cohort | | 203/3,826 | | 2,5 | | 9 | | 30-64 | | 48.7 | | NR | | 77% BMI < 27, 23% BMI≥27 | | 19 | | 5.19 (4.87-5.54) (BMI<27) vs 5.49 (5.11-5,86) (BMI≥27) | | | NR | |  |
| Goessling W, et al FHS | 2008 | | US | | Cohort | | 208/2,812 | | 2,5 | | 20 | | 44 | | 44 | | NR | | 25.6±4.4 | | 32 | | NR | | | NR | |  |
| Grimnes G, et al Tromsø | 2010 | | Norway | | Cohort | | 247/6119 | | 1,2,4 | | 11 | | 55-74 | | 38.6 | | NR | | non-smoker: 26.3±3.8/ smoker: 24.7±3.7 | | 32 | | NR | | | NR | |  |
| Halimi JM, et al DESIR | 2008 | | France | | Cohort | | 171/3,851 | | 2,5 | | 9 | | 30-64 | | 53.4 | | F:19.4/ M:18.1 | | F:24.0±4.0/ M: 25.3±3.2 | | F: 13.3/ M:26.3 | | F: 5.14±0.5/ M: 5.43±0.52 | | | NR | |  |
| Handberg A, et al VIP | 2010 | | Sweden | | nested case-referent | | 173/305 | | 2 | | 10 | | 40-60 | | 54.5 | | NR | | F: 29.8±5.1 (T2D) vs 25.3±4.2 (referent)/ M: 29.2±3.2 (T2D) vs 25.3±3 (referent) | | NR | | F: 5.9±0.7 (T2D) vs 5.2±0.8 (referent)/ M: 5.9±0.8 (T2D) vs 5.3±0.7 (referent) | | | NR | |  |
| Harita N, et al Kansai Healthcare | 2009 | | Japan | | Cohort | | 877/8,570 | | 2,5 | | 4 | | 40-55 | | 100 | | 12.9 | | 23.4±2.9 | | 56.1 | | 97.6±9.5 | | | NR | |  |
| Haugaard SB, et al Danish MONICA 10 | 2012 | | Denmark | | Cohort | | 182/2,353 | | 2,3,4 | | 13.8 | | 56.4±10 (T2D) vs 53.6±10.6 (non-case) | | 48.8 | | NR | | 28.8 (23-37.5) (T2D) vs 25.1 (20.1-32.7) (non-case) | | 46.4 | | 5.2 (4.4-6.7) (T2D) vs 4.7 (4.1-5.5) (non-case) | | | NR | |  |
| Heidemann C, et al Nurses’ Health Study | 2008 | | US | | nested case-control | | 1,038/1136 | | 1,2,5 | | 12 | | 30-55 | | 0 | | 45.2 (T2D) vs 23 (non-case) | | 30.1±5.4 (T2D) vs 26.4±6.1 (control) | | 13.4 (T2D) vs 12.2 (control) | | NR | | | NR | |  |
| Herder C, et al MONICA/KORA | 2011 | | Germany | | Case-cohort | | 436/1,410 | | 1,3,4 | | 11 | | 25-64 | | 47.8 | | 22.1 | | 30.31 (T2D) vs 26.74 (non-case) | | 23.9 (T2D) vs 23.3 (non-case0 | | NR | | | NR | |  |
| Hernestål-Boman J, et al Västerbotten Intervention Programme | 2012 | | Sweden | | nested case-referent | | 157/277 | | 2 | | 5.5 | | 30-60 | | 56.7 | | 31.4 (T2D) vs 16.4(non-case) | | 29.5±4.2 (T2D) vs 25.3±3.6 (non-case) | | 28.2 (T2D) | | 5.9±0.8 (T2D) vs 5.2±0.7 (non-case) | | | NR | |  |
| Hjellvik V, et al Norwegian health Study | 2012 | | Norway | | Cohort | | 2,523/109,796 | | 3 | | 5 | | 40-45 | | 46.2 | | NR | | F:30.8(T2D) vs 24.7 (non-case) /M: 30.1 (T2D) vs 26.1 (non-case) | | NR | | F: 6.0 (T2D) vs 5.1 (non-case)/ M: 6.5 (T2D) vs 5.2 (non-case) | | | NR | |  |
| Hoogeveen RC, et al ARIC | 2007 | | US | | Case-cohort | | 581/572(10,275 cohort) | | 2,5 | | 9 | | 45-64 | | 42.6 (T2D) 36.4 (non-case) | | 35.1 (T2D) 20.5 (non-case) | | 30.5 (T2D) vs 26.8 (non-case) | | 19.8 (T2D) vs 21.9 (non-case) | | 6 (T2D) vs 5.4 (non-case) | | | NR | |  |
| Hu FB, et al Nurses’ Health Study | 2004 | | US | | nested case-control | | 737/785 | | 1,2,5 | | 10 | | 30-55 | | 0 | | 46.8 (T2D) vs 21.0 (non-case) | | 30.1±5.6 (T2D) vs 26.2±6.1 (control) | | 14.1 (T2D) vs 13.3 (non-case) | | NR | | | NR | |  |
| Husemoen LL, et al Inter99 | 2012 | | Denmark | | Cohort | | 141/3917 | | 2 | | 5 | | 30-65 | | 48.4 | | 17.9 | | 26.3±4.65 | | 35.7 | | NR | | | NR | |  |
| IL’Yasova D, et al IRAS | 2012 | | US | | Cohort | | 138/852 | | 2 | | NR | | 40-69 | | NR | | NR | | non-smoker: 26.3±3.8/ smoker: 24.7±3.7 | | NR | | NR | | | NR | |  |
| Ix et al. Health ABC | 2008 | | USA | | Case-cohort | | 112/519 | | 1, 2, 5 | | 6 years | | mean: 73; range: 70-79 | | 50 | | Nr | | Mean: 27; SD: 5 | | Nr | | 92 (median of 2/3 groups divided in tertiles of Fetuin A) | | |  | |  |
| Jobs et al.ULSAM | 2013 | | Sweden | | Cohort | | 30/597 | | 2, 5 | | 6.5 years | | Mean (SD) 71 (0.6) | | 100 | | Nr | | n=100/905 obese | | Nr | | 5.4 (0.6) | | | Nr | |  |
| Juraschek et al. ARIC | 2013 | | USA | | Case-cohort | | 544/533 | | 1, 2, 5 | | 7.9 years | | 52.8 | | 36 | | 24.4 | | Mean: 27.2 | | 20.4% (current) | | mean: 98.2 | | |  | |  |
| Kanaya AM et al. ABC | 2004 | | USA | | Possibly cross-sectional | | 519/519 | | 2, 5 | | Na | | mean (SD) 73.7 (2.9) | | 57.4 | | Nr | | Mean (controls): 27 | | 13.9% (Current in controls) | | 5 | | |  | |  |
| Kim et al. | 2009 | | South Korea | | Cohort | | 3556 | | 2, 5 | | 5 years | | measn: 45.7; range 20-79 | | 62.3 | | Nr | | mean: 23.3 | | Nr | | Nr | | | Nr | |  |
| Krakoff J et al. | 2003 | | USA | | Nested case-control | | 79/79 | | 2 | | 5.7 years | | mean: 32.3 | | 33.8 | | Nr | | Mean: 36.3 | | Nr | | 5.3 | | | 5.4 | |  |
| Krishnan E et al. CARDIA | 2012 | | USA | | Cohort | | 5012 | | 2, 5 | | 15 years | | Mean: 24.8 | | 45.6 | | Nr | | Mean: 24.4 | | 43.1 | | 8.3 | | |  | |  |
| Ko et al, KoGES | 2015 | | Korea | | nested case-control | | 693/698 | | 1,2 | | 6 | | M:51.3, F:53.7 | | 54.4 (T2D) vs 54.6 (non-cases) | | NR | | 25 | | M:39, F:2.5 | | NR | | | NR | |  |
| Laaksonen et al. KIHD | 2004 | | Finland | | Cohort | | 78/762 | | 2, 5 | | 11 years | | Mean: 51.5 | | 100 | | 27 | | Mean: 26.5 | | 26.8 | | 4.5 | | |  | |  |
| Lee et al. EPIC-Norfolk | 2009 | | UK | | nested case-control | | 293/708 | | 1, 2, 5 | | 3.7 years | | Mean (controls): 61.9 | | 57.8 | | 10 | | Mean: 27.6 | | 8.6 (current; controls) | | Nr | | | 5.1 | |  |
| Lee et al. | 2003 | | South Korea | | Cohort | | 83/4088 | | 2 | | 4 years | | Range 25-55 | | 100 | | 10.7 | | Overweight (>=25): 19.9 | | 70.60% | | Nr | | | Nr | |  |
| Lee et al. CARDIA | 2003 | | USA | | Cohort | | 157/4844 | | 2, 5 | | 15 years (up to) | | Range: 18-30 | | 45.4 | | Nr | | 24.47787 | | 29.88875 | | 825.1238646 | | | Nr | |  |
| Lee et al. | 2014 | | China | | Cohort | | 420/5354 | | 2 | | 4.6 | | 61.6±9.3 | | 37.7 | | 8.9 | | 24.2±3.2 | | 16.8 | | 4.99±0.47 mmol/l | | | NR | |  |
| Ley et al. SLHDP | 2010 | | Canada | | Cohort | | 86/492 | | 1, 2, 5 | | 10 years | | Mean (noT2D at follow-up): 25.4 | | 42.6 | | NR | | 25.4 | | NR | | 5.3 | | | NR | |  |
| Lindsay et al. | 2001 | | USA | | Cohort | | 568/2088 | | 2, 3 | | 15.5-16.8 years (NGT baseline) | | 20.85773 | | 46.78548 | | NR | | 23.92409 | | NR | | 20.85773318 | | | NR | |  |
| Liu et al. WHIOS | 2007 | | USA | | nested case-control | | 1584/2198 | | 1, 5 | | 5.9 | | 50-79 | | 0 | | 31-71% (means of different groups) | | 24-34 | | 3.1-11 | | 91-127 | | | NR | |  |
| Luft et al. ARIC | 2010 | | USA | | Case-cohort | | 546/538 | | 1, 2, 5 | | 9 | | 45-64 | | 36.5 | | Nr | | Obese=22.3% | | NR | | NR | | | NR | |  |
| Lyssenko et al. Inter99 and Botnia | 2012 | | Denmark | | nested case-control/ Cohort | | 202/597; 136/2350 | | 2; 2 | | 5; 7 | | 30-60; 45 (non-converters) | | 49; 46 | | 16; 85 | | 25.3; 25.4 | | NR | | 5.4; 5.5 | | | 5.8; 5.4 | |  |
| Ma et al. CHS | 2015 | | USA | | Cohort | | 297/3004 | | 1,5 | | 10 | | 74±5 | | 40 | | NR | | 26±5 | | 9 | | NR | | | NR | |  |
| Mainous et al. NHANES-I | 2002 | | USA | | Cohort | | 9274 | | 1, 6 | | 20 years (up to) | | 25-74 | | 47.7 | | NR | | NR | | NR | | NR | | | NR | |  |
| Mandel et al. NHS | 2012 | | USA | | nested case-control | | 630/730 | | 1, 2, 5 | | 10 years (up to) | | 30-55 | | 0 | | 23 (controls) | | 25.6 | | 10 | | Nr | | | 5.6 | |  |
| Mahendran et al. METSIM | 2014 | | Finland | | Cohort | | 30/735 | | 2 | | 5 | | 54.9±5.7 | | 100 | | NR | | 26.5±3.5 | | NR | | 5.8±0.6 | | | NR | |  |
| Marques-Vidal et al. CoLaus | 2012 | | Switzerland | | Cohort | | 208/3842 | | 1, 2, 5 | | 5.5 | | 35-75 | | 43.3 | | NR | | 25.2 | | NR | | NR | | | NR | |  |
| Mattila et al. MFHS | 2007 | | Finland | | Cohort | | 187/4097 | | 5 | | 17 | | 40-69 | | 47 | | NR | | NR | | NR | | NR | | | NR | |  |
| McMullan et al. NHS | 2013 | | USA | | nested case-control | | 370/370 | | 1, 2, 5 | | 12 | | 64.4 (mean controls) | | 0 | | 18.6 | | 25.3 | | 5.4 | | NR | | | NR | |  |
| Meigs et al. NHS | 2004 | | USA | | nested case-control | | 737/785 | | 1, 2, 5 | | 10 years | | 56.2 (mean controls) | | 0 | | 21 | | 26.2 | | 13.4 | | Nr | | | 5.6 | |  |
| Montonen J, et al EPIC-Potsdam | 2012 | | Germany | | Case-cohort | | 607/1,969 | | 1,3 | | 7 | | 35-65 | | 39.6 | | NR | | 30.4±4.49 (T2D) vs 25.8±4.16 (non-case) | | 64.9 (T2D) vs 51.7 (non-case) | | NR | | | 7.2±.94 (T2D) vs 6.43±.55 (non-case) | |  |
| Montonen J, et al EPIC-Potsdam | 2011 | | Germany | | Case-cohort | | 613/1,965 | | 1,3 | | 7 | | 35-65 | | 39.6 | | NR | | 30.4±4.49 (T2D) vs 25.8±4.16 (non-case) | | 64.9 (T2D) vs 51.7 (non-case) | | NR | | | 7.2±.94 (T2D) vs 6.43±.55 (non-case) | |  |
| Mozaffarian D, et al MESA | 2013 | | US | | Cohort | | 205/2,281 | | 2,5 | | 5 | | 45-84 | | 46.7 | | NR | | 27.7±5.4 | | 12.9 | | 97.6 | | | NR | |  |
| Mozaffarian D, et al CHS | 2010 | | US | | Cohort | | 304/3,736 | | 2,5 | | 10 | | ≥65 | | 42 | | NR | | 26.7 | | 8 | | 5.7 | | | NR | |  |
| Mozaffarian D, et al CHS | 2010 | | US | | Cohort | | 296/3,630 | | 2,5 | | 10 | | ≥65 | | 41.6 | | NR | | 26.72 | | 8 | | 103.2 | | | NR | |  |
| Møller H, et al CCHS | 2011 | | Denmark | | Cohort | | 568/8849 | | 3 | | 18 | | 61 (48-71) | | 44 | | NR | | 25 | | 73.5 | | 5.45 | | | NR | |  |
| Nan et al. | 2008 | | Mauritius | | Cohort | | 716/4259 | | 1,2,5 | | 11 | | M: 44, F: 46 | | 45.6 | | M: 22 F: 29.8 | | M: 24.8, F: 26.5 | | M: 56.1, F; 4.7 | | M: 5.6, F: 5.5 mmol/l | | | NR | |  |
| Neeland et al. DHS | 2012 | | US | | Cohort | | 84/732 | | 2,5 | | 7 | | 45 | | 45.2 | | 63.3 | | 35.4 | | 28.6 | | 133 mg/dl | | | 6.60% | |  |
| Ngarmukos et al. EGAT | 2012 | | Thailand | | Nested c-c | | 63/126 | | 2,3,5 | | 10 | | 47.8 | | 100 | | NR | | 25.9 | | NR | | NR | | | NR | |  |
| Nguyen et al. BHS | 2011 | | US | | Cross-sectional | | 80/1055 | | 2 | | 16 | | 25.6 | | 40 | | NR | | 30.6 | | 39.4 | | 84.5 mg/dl | | | NR | |  |
| Nguyen et al. BHS | 2010 | | US | | Retrospective cohort (children) | | 25/1120 | | 2 | | 17 | | 4yrs-18yrs | | 40 | | NR | | NR (children) | | NR | | NR | | | NR | |  |
| Nilsson et al. GENDER | 2009 | | Sweden | | Longitudinal twin study | | 30/240 | | 1,2,4,5 | | 8 | | 71-80 | | 53.3 | | NR | | NR | | NR | | NR | | | NR | |  |
| Norberg et al. VIP | 2007 | | Sweden | | Nested c-c | | 177/513 | | 3,4 | | 12 | | M: 50.5, F: 52.2 | | 56.7 | | NR | | M: 29.2, F: 29.8 | | NR | | 5.9 mM | | | NR | |  |
| Norberg et al. VIP | 2006 | | Sweden | | nested case-control | | 164/468 | | 3,4 | | 12 | | M: 51.0, F: 53.1 | | 59.6 | | M: 25.0, F; 41.8 | | M: 29.3, F: 30.0 | | M: 29, F; 23 | | M: 6.0, F: 5.8 mmol/l | | | M: 4.7, F: 4.7% | |  |
| Onat et al. TARFS | 2011 | | Turkey | | cohort | | 212/2261 | | 2,5 | | 7.6 | | 47.2±11.4 | | 48.3 | | 22 | | M:26.3/F:28.6 | | 37 | | M:5.31/F:5.39 | | | NR | |  |
| Onat et al. TARFS | 2009 | | Turkey | | Cross-sectional | | 36/193 | | 2,5 | | 4 | | M: 63.4, F: 62.8 | | 52.8 | | NR | | M: 28.4, F: 31.9 | | M: 35, F: 11 | | M: 5.8, F: 5.9 mmol/l | | | NR | |  |
| Onat et al. TARFS | 2007 | | Turkey | | Cohort | | 168/2,219 | | 2,5 | | 6 | | 28-74 | | 48 | | NR | | NR | | NR | | NR | | | NR | |  |
| Patel et al, EPIC-Norfolk | 2014 | | UK | | Case-cohort | | 476/718 | | 1, 2, 5 | | 11 | | 57.7±9.3 | | 41 | | 11 | | 26 | | 13.2 | | NR | | | NR | |  |
| Pradhan AD, et al WHS | 2007 | | US | | Cohort | | 1238/26,563 | | 1 | | 10.1 | | ≥45 | | 0 | | 24.8 | | 25.8 | | 11.6 | | NR | | | 5.03±0.37 | |  |
| Pradhan AD, et al WHS | 2003 | | US | | nested case-control | | 126/225 (27,628 cohort) | | 1 | | 4 | | ≥45 | | 0 | | 45 (T2D) vs 25 (non-case) | | 31.8±6.5 (T2D) vs 25.6±5 (non-case) | | 14.2 | | NR | | | 5.96±0.35 (T2D) vs 5.48±0.28 (non-case) % | |  |
| Pradhan AD, et al WHS | 2001 | | US | | nested case-control | | 188/362 (27,628 cohort) | | 2,5 | | 4 | | ≥45 | | 0 | | 44.2 (T2D) vs 23.8 (no-case) | | 31.8±6.5 (T2D) vs 25.6±5 (non-case) | | 12 | | NR | | | 6 (5.7-6.3) (T2D) vs 5.5 (5.3-5.7) (non-case) % | |  |
| Rathmann W, et al KORA | 2010 | | Germany | | Cohort | | 93/887 | | 2,3 | | 7.5 | | 55-74 | | 49.7 | | 35.2 (T2D) vs 21.2 (non-case) | | 30.2±3.6 (T2D) vs 27.9±4 (non-case) | | 11.9 | | 5.9±0.56 (T2D) vs 5.43±0.49 (non-case) | | | 5.8±0.4 (T2D) vs 5.6±0.3 (non-case) | |  |
| Raynor et al. ARIC | 2013 | | US | | Cohort/case-control | | 10,275/15,792 (c-c 529 cases, 543 controls) | | 1, 2, 3, 5 | | 7.6 | | 54.1/53.9 | | NR | | 33.9/21.3 | | NR | | NR | | 108.1/97.3 mg/dL | | | NR | |  |
| Rhee et al. KBSMC | 2012 | | Korea | | Cohort | | 479/1635 | | 1, 2 | | 4 | | 40.6±6.2 | | 64.9 | | NR | | 23.6±2.9 | | NR | | 96.2±15.2 mg/dl | | | NR | |  |
| Rhee et al. FHS | 2011 | | US | | Cohort | | 2422/5124 | | 2, 5 | | 12 | | 56±9 | | 58 | | 28 | | 30.5±5.0 | | NR | | 105.9±9 | | | NR | |  |
| Rolandsson et al. | 2001 | | Sweden | | Cohort | | 41/2314 | | 2 | | 8 | | 30-60 | | 55 | | NR | | ≤23, 24-25, 26-27, >27 | | NR | | NR | | | NR | |  |
| Sahakyan et al. BDES | 2011 | | US | | Cohort | | 3472/5924 | | 2, 3, 5 | | 15 | | 58.6±10 | | 41.3 | | NR | | 25-29, ≥30 | | 19.2 | | NR | | | 5.6±0.6 | |  |
| Salomaa et al. FINRISK97/Health 2000 | 2010 | | Finland | | Cohort | | 417/7827 | | 3, 4, 5 | | 10.8 | | M: 47.3, F: 44.7 | | 50.1 | | NR | | M: 26.6, F: 25.8 | | M: 26.4, F: 17.4 | | M: 5.1, F: 4.9 | | | NR | |  |
| Santaren et al, IRAS | 2014 | | US | | Cohort | | 103/659 | | 2,5 | | 5 | | 54.7±8.6 | | 45.07 | | NR | | 27.2 (24.8-27.2) | | 14.3 | | NR | | | NR | |  |
| Sattar et al. PROSPER | 2009 | | UK | | Cohort | | 292/5804 | | 1, 2 | | 3.2 | | 75.4±3.3 | | 49.7 | | NR | | 28.8±4.5 | | 22.3 | | 5.87±0.76 mmol/l | | | NR | |  |
| Schulze et al. NHS | 2005 | | US | | Cohort/ nested case-control | | 219/640 | | 1 | | NR | | 56.9 | | 0 | | 42.5 | | 30.2 | | 8.1 | | NR | | | 6.5 ± 1.4 % | |  |
| Schafer et al. SOF | 2014 | | US | | Cohort | | 320/5463 | | 1 | | 8.6 | | ≥65 | | 0 | | NR | | 26.3 | | 5.3 | | NR | | | NR | |  |
| Selvin et al, ARIC | 2014 | | US | | Cohort | | 958/11348 | | 1, 2, 5 | | 20 | | 56.9 | | 53.3 | | 24.2 | | 27.9 | | 21.7 | | 6.2±2.1 mmol/l | | | 5.7±1.1 % | |  |
| Schöttker et al. ESTHER | 2013 | | Germany | | Cohort | | 7791/9949 | | 1, 2, 3, 5 | | 7.9 | | 62 | | 42.4 | | 35.2 | | 26.8 | | 17 | | NR | | | 5.5 | |  |
| Schöttker et al. ESTHER | 2011 | | Germany | | Cohort | | 6803/9953 | | 1, 2, 3, 5 | | 5.02 | | 62 | | 43.2 | | NR | | NR | | NR | | 100-125 mg/dl | | | 5.7-6.4% | |  |
| Shlomai et al. MHS | 2010 | | Israel | | Cohort | | 3352/155904 | | 2,5 | | 6 | | 49 | | 37 | | NR | | NR | | NR | | NR | | | NR | |  |
| Sluijs et al. EPIC-NL | 2013 | | The Netherlands | | nested case-control | | 921/3163 | | 1,4 | | 10 | | 21-70 | | 25 | | 18.6 | | 24.2-27.5 | | 30.5 | | NR | | | NR | |  |
| Song et al. WHIOS | 2007 | | US | | Case- cohort | | 1584/3782 | | 5 | | 5.9 | | 62.7 | | 0 | | 57.5 | | 32.3 | | 7.74 | | 6.78 mmol/ml | | | NR | |  |
| Song et al. WHIOS | 2007 | | US | | nested case-control | | 1584/3782 | | 1,3 | | 5.9 | | 62.3 | | 0 | | 63 | | 31.3 | | 7.3 | | NR | | | NR | |  |
| Soulimane et al. Inter99, AusDiab, DESIR | 2011 | | Denmark, Australia, France | | Cohort | | Inter99: 132/4703, AusDiab: 186/6025, DESIR: 92/3784 | | 2, 5 | | Inter99: 5 yrs, AusDiab: 5yrs, DESIR: 6 yrs | | Inter99: 50±6, AusDiab: 56±12, DESIR: 52±9 | | Inter99: 67.5, AusDiab: 51.6, DESIR: 68.5 | | NR | | Inter99: 30.1±4.8, AusDiab: 30.6±5.7, DESIR: 28.7±4.8 | | NR | | Inter99: 8.1±2.3, AusDiab: 8.6±2.3 mmol/l, DESIR: NR | | | Inter99: 5.4±0.4, AusDiab: 5.5±0.3, DESIR: 5.4±0.4 | |  |
| Stefan et al. EPIC- Potsdam | 2008 | | Germany | | Case- cohort | | 703/2164 | | 1, 5 | | 7.1 | | Q1: 51.0, Q2: 50.4, Q3: 49.9, Q4: 48.8, Q5: 47.6 | | 37.8 | | NR | | Q1: 25.6, Q2: 26.1, Q3: 26.1, Q4: 25.8, Q5: 26.2 | | NR | | Q1: 85.8, Q2: 87.6, Q3: 87.3, Q4: 87.2, Q5: 86.9 mg/dl | | | NR | |  |
| Stefan et al. NHS | 2014 | | US | | nested case-control | | 470/470 | | 1,2 | | 6 | | 53-79 | | NR | | NR | | NR | | NR | | NR | | | NR | |  |
| Steffen et al. MESA | 2015 | | US | | Cohort | | 657/5697 | | 1,2,5 | | 11.4 | | 61.7 | | 46.3 | | NR | | 28 | | 13.1 | | 89.6 mg/dl | | | NR | |  |
| Stranges et al. WNYS | 2008 | | US | | Epi case-control | | 61/219 | | 2,5 | | 5.9 | | 58.1 | | 57.4 | | 44.1 | | 32.2 | | 19.7 | | 112.9 mg/dl | | | NR | |  |
| Sun et al. NHAPC | 2013 | | China | | Prospective cohort | | 538/2198 | | 1,2 | | 6 | | 50-70 | | Q1: 42.8, Q2: 43.1, Q3: 42.9, Q4: 43.1, Q5: 43.0 | | Q1: 9.6, Q2: 8.4, Q3: 10.2, Q4: 10.5, Q5: 11.4 | | Q1: 23.4±.3.3, Q2: 24.2±3.6, Q3: 24.1±3.5, Q4: 24.7±3.6, Q5: 24.9±3.6 | | Q1: 26.2, Q2: 28.3, Q3: 29.0, Q4: 29.2, Q5: 30.0 | | Q1: 5.17±0.55, Q2: 5.33±0.53, Q3: 5.36±0.53, Q4: 5.42±0.55, Q5: 5.52±0.60 mmol/l | | | Q1: 5.67±0.44, Q2:5.68±0.38,Q3:5.68±0.4, Q4:5.74±0.45,Q5:5.75±0.45 | |  |
| Sun et al. NHS | 2010 | | US | | Cohort | | 1054/32826 | | 1,2,5 | | 15 | | 56 | | 0 | | 44.7 | | 30.3±5.5 | | 13.2 | | NR | | | NR | |  |
| Tabak et al. Whitehall II | 2009 | | UK | | Prospective cohort | | 505/6538 | | 1,2 | | 9.7 | | 53.1±6.6 | | 66.3 | | NR | | 28.18±4.99 | | NR | | 7.06±2.48 mmol/l | | | NR | |  |
| Thorand et al. MONICA/KORA Augsburg | 2010 | | Germany | | Prospective case-cohort | | 460/1934 | | 1,3,5 | | 10.9 | | M: 56.10, F: 56.03 | | 50.6 | | M: 24.1, F: 33.3 | | M: 29.75, F: 30.94 | | M: 77, F: 32.3 | | NR | | | NR | |  |
| Thorand et al. MONICA/KORA Augsburg | 2007 | | Germany | | Prospective case-cohort | | 224/1204 | | 1,3,4 | | 8 | | NR | | 55.8 | | 27.7 | | 30.2 | | 27.2 | | NR | | | NR | |  |
| Thorand et al. MONICA/KORA Augsburg | 2007 | | Germany | | Prospective case-cohort | | 527/2225 | | 1,3 | | 10.8 | | M: 56.1, F: 56.2 | | 53.7 | | M: 24.6, F: 33.3 | | M: 29.6, F: 30.9 | | M: 35.4, F; 15.3 | | NR | | | NR | |  |
| Thorand et al. MONICA/KORA Augsburg | 2006 | | Germany | | Prospective case-cohort | | 532/2244 | | 1,3,4 | | 18? | | M: 56.1, F: 56.2 | | 54 | | M: 24.5, F: 33.3 | | M: 29.7, F: 30.9 | | M: 35.1, F: 15.3 | | NR | | | NR | |  |
| Thorand et al. MONICA/KORA Augsburg | 2005 | | Germany | | Prospective case-cohort | | 527/2225 | | 1,3,4 | | 10.8 | | 56.1 | | 53.7 | | 16.9 | | 30.2±0.2 | | 20.6 | | NR | | | NR | |  |
| Thorand et al. MONICA | 2003 | | Germany | | Prospective case-cohort | | 101/2052 | | 1,3 | | 7.2 | | 57.2 | | 100 | | 30.7 | | 29.8 | | 32.7 | | NR | | | NR | |  |
| Urdea et al. Inter99 | 2009 | | Denmark | | Cohort/ nested case-control | | 202/799 | | 2 | | 5 | | 50 | | 65.7 | | 33.3 | | 28.6 | | NR | | 6.1 mmol/l | | | NR | |  |
| Vaccaro et al. GPS | 2005 | | Italy | | Prospective cohort | | 101/2103 | | 2,5 | | 6 | | 55.7 | | 45 | | NR | | 30.1 | | NR | | 5.6 mmol/l | | | NR | |  |
| Vigo et al. ARIC | 2007 | | US | | Case-cohort | | 1124/10275 | | 2,3,5 | | ~9 | | 51.6 | | 38.6 | | 32.3 | | 27.2 | | NR | | NR | | | NR | |  |
| Vozarove et al. GRIC | 2002 | | US | | Longitudinal cohort/cross-sectional | | 63/451 | | 2 | | 6.9 | | 30 | | NR | | NR | | NR | | NR | | 4.9 mmol/l | | | NR | |  |
| Vozarova et al. GRIC | 2002 | | US | | Longitudinal cohort/cross-sectional | | 54/352 | | 2 | | 5.5 | | 27 | | 61.1 | | NR | | NR | | NR | | 5.2 mmol/l | | | NR | |  |
| Wang et al. Baoshan District, Shanghai | 2011 | | China | | Cohort | | 924/1284 | | 1, 2 | | 3.5 | | 61.6 | | 35.9 | | 15.3 | | 26 | | 13.5 | | 6.1±0.6 mmol/l | | | NR | |  |
| Wang et al. FOS/MDC | 2013 | | US | | Longitudinal cohort/#case-cohort | | 376/1937 | | 2,5 | | 12 | | 56 ± 9 | | 57 | | 32 | | 30.5 | | NR | | 105 ± 9 mg/dl | | | 5.50% | |  |
| Wang et al. FOS | 2011 | | US | | Longitudinal cohort/  case-cohort | | 201/2422 | | 2,5 | | 12 | | 56.9 | | 58 | | 31 | | 30.5 | | NR | | 105 ± 9 mg/dl | | | NR | |  |
| Wang et al. NTAC | 2006 | | Australia | | nested case-control | | 117/882 | | 2 | | 11 | | 36.4 | | 37.6 | | NR | | 26.7 | | 72.6 | | 5.7 mmol/l | | | NR | |  |
| Wang-Sattler et al. KORA | 2012 | | Germany | | Prospective case-cohort | | 134/1010 | | 1 | | NR | | 65.5 | | 65.9 | | NR | | 34.1 | | 14.3 | | 106.1±10.1 mg/dl | | | 5.8±0.4 % | |  |
| Wannamethee et al. BRHS/BWHHS | 2011 | | UK | | Prospective cohort | | 297/6927 | | 2,3 | | 7 | | 68.4 | | 57 | | 22.5 | | 30.4 | | 10.1 | | 6.00 mmol/l | | | 4.84±0.62% (T2D) vs 5.23±0.64 (non-cases) | |  |
| Wannamethee et al. BRHS | 2008 | | UK | | Prospective cohort | | 162/3562 | | 1 | | 7 | | 68 | | 100 | | 9.9 | | 29.7 | | 9.3 | | 5.99 mmol/l | | | NR | |  |
| Welsh et al. PROSPER | 2009 | | Scotland/Ireland/Netherlands | | Cohort | | 289/4934 | | 1,2,5 | | 3.2 | | 75.3 | | 50 | | NR | | 28.7 | | 22 | | 5.81 mmol/l | | | NR | |  |
| Wu et al. MCCS, VIP, EPIC-Norfolk, CHS, EPIC-Potsdam | 2007-2011 | | Australia, Sweden, UK,US,Germany | | Cohort/  case-cohort | | 346,159,199,204,673/3737,450,383,3088,2724 | | 1,2,3,4,5 | | 41,8.8,10.3,9.6,6.3 | | 55,52,64,75,51 | | 44.1,58.4,53.3,38.9,43.5 | | NR | | 27,27,28.1,26.4,26.9 | | NR | | NR | | | NR | |  |
| Xu et al. Shanghai | 2011 | | China | | Cohort | | 19/526 | | 2 | | 3.5 | | 60.6 | | 36.8 | | 10.5 | | 26.2 | | 15.8 | | 5.46 ± 0.26 mmol/l | | | NR | |  |
| Zheng et al. Sichuan | 2014 | | China | | Cohort | | 41/474 | | NR | | 4 | | 18-70 | | 53.7 (T2D) vs 35 (non-cases) | | 19.5 (T2D) vs 15 (non-cases) | | 23.8 (T2D) vs 23.2(non-cases) | | 19.5 (T2D) vs 17.5 (non-cases) | | 4.65 (T2D) vs 4.6 (non-cases) | | | NR | |  |
|  |  | |  | |  | |  | |  | |  | |  | |  | |  | |  | |  | |  | | |  | |  |
| *Diabetes definition criteria: | | | | | | | | | | | | | | | | | | | | | | | | |  | | | |
| 1- self report or questionnaire, telephone interview | | | | | | | | | | | | | | | | | | | | | | | | |  | | | |
| 2- fasting glucose, non-fasting or 2 h plasma glucose 3- HbA1c, | | | | | | | | | | | | | | | | | | | | | | | | |  | | | |
| 3- physician diagnosed in survey or primary-care data | | | | | | | | | | | | | | | | | | | | | | | | |  | | | |
| 4- hospital records or review of patient’s notes | | | | | | | | | | | | | | | | | | | | | | | | |  | | | |
| 5- drug use or pharmacy registry data | | | | | | | | | | | | | | | | | | | | | | | | |  | | | |

| Abbreviations:  Not reported | NR |
| --- | --- |
| Not applicable | NA |
| Not significant | NS |
| Female | F |
| Male | M |
| Black | B |
| White | W |
| European Prospective Investigation into Cancer and Nutrition - the Netherlands | EPIC-NL |
| Prevention of Renal and Vascular End-stage Disease | PREVEND |
| Data from the Epidemiological Study on the Insulin Resistance | DESIR |
| Metabolic Syndrome Berlin Potsdam | MeSyBePo |
| Atherosclerosis Risk in Communities Study | ARIC |
| Women’s Health Initiative Observational Study | WHIOS |
| Chin-Shan Community Cardiovascular Cohort Study | CSCCHS |
| Copenhagen City Heart Study | CCHS |
| South-West Seoul | SWS |
| Framingham heart study | FHS |
| Cardiovascular Health Study | CHS |
| Malmö Diet and Cancer Study | MDC |
| MONItoring trends and determinants of CArdiovascular disease | MONICA |
| Insulin Resistance Atherosclerosis Study | IRAS |
| Västerbotten Intervention Study | VIP |
| MONItoring of trends and determinants in CArdiovascular disease/Cooperative Health Research in the Region of Augsburg | MONICA/KORA |
| Multi-Ethnic Study of Atherosclerosis | MESA |
| Data from the Epidemiological Study on Insulin Resistance | DESIR |
| Atherosclerosis Risk in Communities | ARIC |
| Kangbuk Samsung Medical Center - adipokine study | KBSMC |
| Framingham Heart Study | FHS |
| Beaver Dam Eye Study | BDES |
| Prospective Study of Pravastatin in the Elderly at Risk Trial Study Group | PROSPER |
| Epidemiologische Studie zu Chancen der Verhütung, Früherkennung und optimierten Therapie chronischer Erkrankungen in der älteren Bevölkerung | ESTHER |
| Västerbotten Intervention Program | VIP |
| Finland Cardiovascular Risk Study (survey year 1997) | FINRISK97 |
| Maccabi Healthcare Services | MHS |
| Australian Diabetes, Obsesity and Lifestyle Study | AusDiab |
| Western New York Study | WNYS |
| The Nutrition and Health of Aging Population in China | NHAPC |
| Nurses' Health Study | NHS |
| Monitoring of trends and determinants in cardiovascular disease | MONICA |
| Cooperative Health Research in the Region of Augsburg | KORA |
| Gubbio Population Study | GPS |
| British Regional Heart Study | BRHS |
| British Women's Heart and Health Study | BWHHS |
| Northern Territory Aboriginal Community | NTAC |
| Farmingham Offspring Study | FOS |
| Malmo Diet and Cancer Study | MDC |
| Gila River Indian Community | GRIC |
| Dallas Heart Study | DHS |
| Electricity Generating Authority of Thailand | EGAT |
| Bogalusa Heart Study | BHS |
| Swedish Aging in Women and Men: A Longitudinal Study of Gender Differences in Health Behaviour and Health Among Elderly | GENDER |
| Turkish Adult Risk Factor Study | TARFS |
| Women's Health Initiative Observational Study | WHIOS |
| Korean Genome & Epidemiology Study | KoGES |
| Prevencion con Dieta Mediterranea | KoGES |
| Study of Osteoporotic Fractures | SOF |

**References:**

1. Abbasi A, Peelen LM, Corpeleijn E, van der Schouw YT, Stolk RP, Spijkerman AM, et al. Prediction models for risk of developing type 2 diabetes: systematic literature search and independent external validation study. BMJ (Clinical research ed). 2012;345:e5900. Epub 2012/09/20. doi: 10.1136/bmj.e5900. PubMed PMID: 22990994; PubMed Central PMCID: PMC3445426.

2. Abbasi A, Corpeleijn E, Meijer E, Postmus D, Gansevoort RT, Gans RO, et al. Sex differences in the association between plasma copeptin and incident type 2 diabetes: the Prevention of Renal and Vascular Endstage Disease (PREVEND) study. Diabetologia. 2012;55(7):1963-70. Epub 2012/04/25. doi: 10.1007/s00125-012-2545-x. PubMed PMID: 22526609.

3. Abbasi A, Bakker SJ, Corpeleijn E, van der AD, Gansevoort RT, Gans RO, et al. Liver function tests and risk prediction of incident type 2 diabetes: evaluation in two independent cohorts. PloS one. 2012;7(12):e51496. Epub 2013/01/04. doi: 10.1371/journal.pone.0051496. PubMed PMID: 23284703; PubMed Central PMCID: PMC3524238.

4. Abbasi A, Corpeleijn E, Postmus D, Gansevoort RT, de Jong PE, Gans RO, et al. Plasma procalcitonin and risk of type 2 diabetes in the general population. Diabetologia. 2011;54(9):2463-5. Epub 2011/06/16. doi: 10.1007/s00125-011-2216-3. PubMed PMID: 21674177.

5. Alessi MC, Nicaud V, Scroyen I, Lange C, Saut N, Fumeron F, et al. Association of vitronectin and plasminogen activator inhibitor-1 levels with the risk of metabolic syndrome and type 2 diabetes mellitus. Results from the D.E.S.I.R. prospective cohort. Thrombosis and haemostasis. 2011;106(3):416-22. Epub 2011/07/30. doi: 10.1160/th11-03-0179. PubMed PMID: 21800006.

6. Andre P, Balkau B, Born C, Royer B, Wilpart E, Charles MA, et al. Hepatic markers and development of type 2 diabetes in middle aged men and women: a three-year follow-up study. The D.E.S.I.R. Study (Data from an Epidemiological Study on the Insulin Resistance syndrome). Diabetes & metabolism. 2005;31(6):542-50. Epub 2005/12/17. PubMed PMID: 16357802.

7. Becerra-Tomas N, Estruch R, Bullo M, Casas R, Diaz-Lopez A, Basora J, et al. Increased serum calcium levels and risk of type 2 diabetes in individuals at high cardiovascular risk. Diabetes care. 2014;37(11):3084-91. Epub 2014/08/21. doi: 10.2337/dc14-0898. PubMed PMID: 25139884.

8. Bobbert T, Schwarz F, Fischer-Rosinsky A, Pfeiffer AF, Mohlig M, Mai K, et al. Fibroblast growth factor 21 predicts the metabolic syndrome and type 2 diabetes in Caucasians. Diabetes care. 2013;36(1):145-9. Epub 2012/08/31. doi: 10.2337/dc12-0703. PubMed PMID: 22933429; PubMed Central PMCID: PMC3526237.

9. Brantsma AH, Bakker SJ, Hillege HL, de Zeeuw D, de Jong PE, Gansevoort RT, et al. Urinary albumin excretion and its relation with C-reactive protein and the metabolic syndrome in the prediction of type 2 diabetes. Diabetes care. 2005;28(10):2525-30. Epub 2005/09/28. PubMed PMID: 16186291.

10. Brunner EJ, Kivimaki M, Witte DR, Lawlor DA, Davey Smith G, Cooper JA, et al. Inflammation, insulin resistance, and diabetes--Mendelian randomization using CRP haplotypes points upstream. PLoS medicine. 2008;5(8):e155. Epub 2008/08/15. doi: 10.1371/journal.pmed.0050155. PubMed PMID: 18700811; PubMed Central PMCID: PMC2504484.

11. Carnethon MR, Palaniappan LP, Burchfiel CM, Brancati FL, Fortmann SP. Serum insulin, obesity, and the incidence of type 2 diabetes in black and white adults: the atherosclerosis risk in communities study: 1987-1998. Diabetes care. 2002;25(8):1358-64. Epub 2002/07/30. PubMed PMID: 12145235; PubMed Central PMCID: PMC3132185.

12. Chao C, Song Y, Cook N, Tseng CH, Manson JE, Eaton C, et al. The lack of utility of circulating biomarkers of inflammation and endothelial dysfunction for type 2 diabetes risk prediction among postmenopausal women: the Women's Health Initiative Observational Study. Archives of internal medicine. 2010;170(17):1557-65. Epub 2010/09/30. doi: 10.1001/archinternmed.2010.312. PubMed PMID: 20876407; PubMed Central PMCID: PMC3127580.

13. Chien KL, Chen MF, Hsu HC, Chang WT, Su TC, Lee YT, et al. Plasma uric acid and the risk of type 2 diabetes in a Chinese community. Clinical chemistry. 2008;54(2):310-6. Epub 2007/12/20. doi: 10.1373/clinchem.2007.095190. PubMed PMID: 18089655.

14. Choi JH, Rhee EJ, Bae JC, Park SE, Park CY, Cho YK, et al. Increased risk of type 2 diabetes in subjects with both elevated liver enzymes and ultrasonographically diagnosed nonalcoholic fatty liver disease: a 4-year longitudinal study. Archives of medical research. 2013;44(2):115-20. Epub 2013/02/13. doi: 10.1016/j.arcmed.2013.01.007. PubMed PMID: 23398788.

15. Choi KM, Lee J, Lee KW, Seo JA, Oh JH, Kim SG, et al. Serum adiponectin concentrations predict the developments of type 2 diabetes and the metabolic syndrome in elderly Koreans. Clinical endocrinology. 2004;61(1):75-80. Epub 2004/06/24. doi: 10.1111/j.1365-2265.2004.02063.x. PubMed PMID: 15212647.

16. Daimon M, Oizumi T, Saitoh T, Kameda W, Hirata A, Yamaguchi H, et al. Decreased serum levels of adiponectin are a risk factor for the progression to type 2 diabetes in the Japanese Population: the Funagata study. Diabetes care. 2003;26(7):2015-20. Epub 2003/07/02. PubMed PMID: 12832305.

17. Dallmeier D, Larson MG, Wang N, Fontes JD, Benjamin EJ, Fox CS. Addition of inflammatory biomarkers did not improve diabetes prediction in the community: the framingham heart study. Journal of the American Heart Association. 2012;1(4):e000869. Epub 2012/11/07. doi: 10.1161/jaha.112.000869. PubMed PMID: 23130155; PubMed Central PMCID: PMC3487343.

18. Djousse L, Biggs ML, Lemaitre RN, King IB, Song X, Ix JH, et al. Plasma omega-3 fatty acids and incident diabetes in older adults. The American journal of clinical nutrition. 2011;94(2):527-33. Epub 2011/05/20. doi: 10.3945/ajcn.111.013334. PubMed PMID: 21593500; PubMed Central PMCID: PMC3142727.

19. Doi Y, Kiyohara Y, Kubo M, Ninomiya T, Wakugawa Y, Yonemoto K, et al. Elevated C-reactive protein is a predictor of the development of diabetes in a general Japanese population: the Hisayama Study. Diabetes care. 2005;28(10):2497-500. Epub 2005/09/28. PubMed PMID: 16186286.

20. Doi Y, Kubo M, Yonemoto K, Ninomiya T, Iwase M, Tanizaki Y, et al. Liver enzymes as a predictor for incident diabetes in a Japanese population: the Hisayama study. Obesity (Silver Spring, Md). 2007;15(7):1841-50. Epub 2007/07/20. doi: 10.1038/oby.2007.218. PubMed PMID: 17636103.

21. Enhorning S, Bankir L, Bouby N, Struck J, Hedblad B, Persson M, et al. Copeptin, a marker of vasopressin, in abdominal obesity, diabetes and microalbuminuria: the prospective Malmo Diet and Cancer Study cardiovascular cohort. International journal of obesity (2005). 2013;37(4):598-603. Epub 2012/05/23. doi: 10.1038/ijo.2012.88. PubMed PMID: 22614056.

22. Engstrom G, Smith JG, Persson M, Nilsson PM, Melander O, Hedblad B. Red cell distribution width, haemoglobin A1c and incidence of diabetes mellitus. Journal of internal medicine. 2014;276(2):174-83. Epub 2014/01/30. doi: 10.1111/joim.12188. PubMed PMID: 24471821.

23. Eugen-Olsen J, Andersen O, Linneberg A, Ladelund S, Hansen TW, Langkilde A, et al. Circulating soluble urokinase plasminogen activator receptor predicts cancer, cardiovascular disease, diabetes and mortality in the general population. Journal of internal medicine. 2010;268(3):296-308. Epub 2010/06/22. doi: 10.1111/j.1365-2796.2010.02252.x. PubMed PMID: 20561148.

24. Fagerberg B, Kellis D, Bergstrom G, Behre CJ. Adiponectin in relation to insulin sensitivity and insulin secretion in the development of type 2 diabetes: a prospective study in 64-year-old women. Journal of internal medicine. 2011;269(6):636-43. Epub 2011/01/05. doi: 10.1111/j.1365-2796.2010.02336.x. PubMed PMID: 21198995.

25. Ferrannini E, Natali A, Camastra S, Nannipieri M, Mari A, Adam KP, et al. Early metabolic markers of the development of dysglycemia and type 2 diabetes and their physiological significance. Diabetes. 2013;62(5):1730-7. Epub 2012/11/20. doi: 10.2337/db12-0707. PubMed PMID: 23160532; PubMed Central PMCID: PMC3636608.

26. Festa A, D'Agostino R, Jr., Tracy RP, Haffner SM, Insulin Resistance Atherosclerosis S. Elevated levels of acute-phase proteins and plasminogen activator inhibitor-1 predict the development of type 2 diabetes: the insulin resistance atherosclerosis study. Diabetes. 2002;51(4):1131-7. Epub 2002/03/28. PubMed PMID: 11916936.

27. Floegel A, Stefan N, Yu Z, Muhlenbruch K, Drogan D, Joost HG, et al. Identification of serum metabolites associated with risk of type 2 diabetes using a targeted metabolomic approach. Diabetes. 2013;62(2):639-48. Epub 2012/10/09. doi: 10.2337/db12-0495. PubMed PMID: 23043162; PubMed Central PMCID: PMC3554384.

28. Ford ES, Schulze MB, Bergmann MM, Thamer C, Joost HG, Boeing H. Liver enzymes and incident diabetes: findings from the European Prospective Investigation into Cancer and Nutrition (EPIC)-Potsdam Study. Diabetes care. 2008;31(6):1138-43. Epub 2008/03/19. doi: 10.2337/dc07-2159. PubMed PMID: 18346992.

29. Gast GC, Spijkerman AM, Van der AD, Jacobs-van der Bruggen MA, Verschuren WM. Five-year changes in biologic risk factors and risk of type 2 diabetes: are attained but not initial risk factor levels of importance? American journal of epidemiology. 2012;176(8):720-5. Epub 2012/09/28. doi: 10.1093/aje/kws189. PubMed PMID: 23013621.

30. Gautier A, Balkau B, Lange C, Tichet J, Bonnet F, Group DS. Risk factors for incident type 2 diabetes in individuals with a BMI of <27 kg/m2: the role of gamma-glutamyltransferase. Data from an Epidemiological Study on the Insulin Resistance Syndrome (DESIR). Diabetologia. 2010;53(2):247-53. Epub 2009/11/26. doi: 10.1007/s00125-009-1602-6. PubMed PMID: 19936701.

31. Goessling W, Massaro JM, Vasan RS, D'Agostino RB, Sr., Ellison RC, Fox CS. Aminotransferase levels and 20-year risk of metabolic syndrome, diabetes, and cardiovascular disease. Gastroenterology. 2008;135(6):1935-44, 44 e1. Epub 2008/11/18. doi: 10.1053/j.gastro.2008.09.018. PubMed PMID: 19010326; PubMed Central PMCID: PMC3039001.

32. Grimnes G, Emaus N, Joakimsen RM, Figenschau Y, Jenssen T, Njolstad I, et al. Baseline serum 25-hydroxyvitamin D concentrations in the Tromso Study 1994-95 and risk of developing type 2 diabetes mellitus during 11 years of follow-up. Diabetic medicine : a journal of the British Diabetic Association. 2010;27(10):1107-15. Epub 2010/09/22. doi: 10.1111/j.1464-5491.2010.03092.x. PubMed PMID: 20854377.

33. Halimi JM, Bonnet F, Lange C, Balkau B, Tichet J, Marre M, et al. Urinary albumin excretion is a risk factor for diabetes mellitus in men, independently of initial metabolic profile and development of insulin resistance. The DESIR Study. Journal of hypertension. 2008;26(11):2198-206. Epub 2008/10/16. doi: 10.1097/HJH.0b013e328310ddff. PubMed PMID: 18854761.

34. Handberg A, Norberg M, Stenlund H, Hallmans G, Attermann J, Eriksson JW. Soluble CD36 (sCD36) clusters with markers of insulin resistance, and high sCD36 is associated with increased type 2 diabetes risk. The Journal of clinical endocrinology and metabolism. 2010;95(4):1939-46. Epub 2010/02/09. doi: 10.1210/jc.2009-2002. PubMed PMID: 20139232.

35. Harita N, Hayashi T, Sato KK, Nakamura Y, Yoneda T, Endo G, et al. Lower serum creatinine is a new risk factor of type 2 diabetes: the Kansai healthcare study. Diabetes care. 2009;32(3):424-6. Epub 2008/12/17. doi: 10.2337/dc08-1265. PubMed PMID: 19074997; PubMed Central PMCID: PMC2646021.

36. Haugaard SB, Andersen O, Hansen TW, Eugen-Olsen J, Linneberg A, Madsbad S, et al. The immune marker soluble urokinase plasminogen activator receptor is associated with new-onset diabetes in non-smoking women and men. Diabetic medicine : a journal of the British Diabetic Association. 2012;29(4):479-87. Epub 2011/11/05. doi: 10.1111/j.1464-5491.2011.03513.x. PubMed PMID: 22050462.

37. Heidemann C, Sun Q, van Dam RM, Meigs JB, Zhang C, Tworoger SS, et al. Total and high-molecular-weight adiponectin and resistin in relation to the risk for type 2 diabetes in women. Annals of internal medicine. 2008;149(5):307-16. Epub 2008/09/04. PubMed PMID: 18765700; PubMed Central PMCID: PMC3874083.

38. Herder C, Baumert J, Zierer A, Roden M, Meisinger C, Karakas M, et al. Immunological and cardiometabolic risk factors in the prediction of type 2 diabetes and coronary events: MONICA/KORA Augsburg case-cohort study. PloS one. 2011;6(6):e19852. Epub 2011/06/16. doi: 10.1371/journal.pone.0019852. PubMed PMID: 21674000; PubMed Central PMCID: PMC3108947.

39. Hernestal-Boman J, Norberg M, Jansson JH, Eliasson M, Eriksson JW, Lindahl B, et al. Signs of dysregulated fibrinolysis precede the development of type 2 diabetes mellitus in a population-based study. Cardiovascular diabetology. 2012;11:152. Epub 2012/12/20. doi: 10.1186/1475-2840-11-152. PubMed PMID: 23249721; PubMed Central PMCID: PMC3538597.

40. Hjellvik V, Sakshaug S, Strom H. Body mass index, triglycerides, glucose, and blood pressure as predictors of type 2 diabetes in a middle-aged Norwegian cohort of men and women. Clinical epidemiology. 2012;4:213-24. Epub 2012/09/01. doi: 10.2147/clep.s31830. PubMed PMID: 22936857; PubMed Central PMCID: PMC3429151.

41. Hoogeveen RC, Ballantyne CM, Bang H, Heiss G, Duncan BB, Folsom AR, et al. Circulating oxidised low-density lipoprotein and intercellular adhesion molecule-1 and risk of type 2 diabetes mellitus: the Atherosclerosis Risk in Communities Study. Diabetologia. 2007;50(1):36-42. Epub 2006/12/01. doi: 10.1007/s00125-006-0533-8. PubMed PMID: 17136392.

42. Hu FB, Meigs JB, Li TY, Rifai N, Manson JE. Inflammatory markers and risk of developing type 2 diabetes in women. Diabetes. 2004;53(3):693-700. Epub 2004/02/28. PubMed PMID: 14988254.

43. Husemoen LL, Thuesen BH, Fenger M, Jorgensen T, Glumer C, Svensson J, et al. Serum 25(OH)D and type 2 diabetes association in a general population: a prospective study. Diabetes care. 2012;35(8):1695-700. Epub 2012/06/13. doi: 10.2337/dc11-1309. PubMed PMID: 22688545; PubMed Central PMCID: PMC3402265.

44. Il'yasova D, Spasojevic I, Base K, Zhang H, Wang F, Young SP, et al. Urinary F2-isoprostanes as a biomarker of reduced risk of type 2 diabetes. Diabetes care. 2012;35(1):173-4. Epub 2011/11/22. doi: 10.2337/dc11-1502. PubMed PMID: 22100959; PubMed Central PMCID: PMC3241306.

45. Ix JH, Wassel CL, Kanaya AM, Vittinghoff E, Johnson KC, Koster A, et al. Fetuin-A and incident diabetes mellitus in older persons. Jama. 2008;300(2):182-8. Epub 2008/07/10. doi: 10.1001/jama.300.2.182. PubMed PMID: 18612115; PubMed Central PMCID: PMC2779582.

46. Jobs E, Riserus U, Ingelsson E, Sundstrom J, Jobs M, Nerpin E, et al. Serum cathepsin S is associated with decreased insulin sensitivity and the development of type 2 diabetes in a community-based cohort of elderly men. Diabetes care. 2013;36(1):163-5. Epub 2012/08/28. doi: 10.2337/dc12-0494. PubMed PMID: 22923671; PubMed Central PMCID: PMC3526243.

47. Juraschek SP, Shantha GP, Chu AY, Miller ER, 3rd, Guallar E, Hoogeveen RC, et al. Lactate and risk of incident diabetes in a case-cohort of the atherosclerosis risk in communities (ARIC) study. PloS one. 2013;8(1):e55113. Epub 2013/02/06. doi: 10.1371/journal.pone.0055113. PubMed PMID: 23383072; PubMed Central PMCID: PMC3559502.

48. Kanaya AM, Harris T, Goodpaster BH, Tylavsky F, Cummings SR, Health A, et al. Adipocytokines attenuate the association between visceral adiposity and diabetes in older adults. Diabetes care. 2004;27(6):1375-80. Epub 2004/05/27. PubMed PMID: 15161791.

49. Kim CH, Park JY, Lee KU, Kim JH, Kim HK. Association of serum gamma-glutamyltransferase and alanine aminotransferase activities with risk of type 2 diabetes mellitus independent of fatty liver. Diabetes/metabolism research and reviews. 2009;25(1):64-9. Epub 2008/12/10. doi: 10.1002/dmrr.890. PubMed PMID: 19065605.

50. Krakoff J, Funahashi T, Stehouwer CD, Schalkwijk CG, Tanaka S, Matsuzawa Y, et al. Inflammatory markers, adiponectin, and risk of type 2 diabetes in the Pima Indian. Diabetes care. 2003;26(6):1745-51. Epub 2003/05/27. PubMed PMID: 12766104.

51. Krishnan E, Pandya BJ, Chung L, Hariri A, Dabbous O. Hyperuricemia in young adults and risk of insulin resistance, prediabetes, and diabetes: a 15-year follow-up study. American journal of epidemiology. 2012;176(2):108-16. Epub 2012/07/04. doi: 10.1093/aje/kws002. PubMed PMID: 22753829.

52. Ko KP, Kim CS, Ahn Y, Park SJ, Kim YJ, Park JK, et al. Plasma isoflavone concentration is associated with decreased risk of type 2 diabetes in Korean women but not men: results from the Korean Genome and Epidemiology Study. Diabetologia. 2015;58(4):726-35. Epub 2014/12/24. doi: 10.1007/s00125-014-3463-x. PubMed PMID: 25533387.

53. Laaksonen DE, Niskanen L, Nyyssonen K, Punnonen K, Tuomainen TP, Valkonen VP, et al. C-reactive protein and the development of the metabolic syndrome and diabetes in middle-aged men. Diabetologia. 2004;47(8):1403-10. Epub 2004/08/17. doi: 10.1007/s00125-004-1472-x. PubMed PMID: 15309290.

54. Lee CC, Adler AI, Sandhu MS, Sharp SJ, Forouhi NG, Erqou S, et al. Association of C-reactive protein with type 2 diabetes: prospective analysis and meta-analysis. Diabetologia. 2009;52(6):1040-7. Epub 2009/03/28. doi: 10.1007/s00125-009-1338-3. PubMed PMID: 19326095.

55. Lee DH, Ha MH, Kim JH, Christiani DC, Gross MD, Steffes M, et al. Gamma-glutamyltransferase and diabetes--a 4 year follow-up study. Diabetologia. 2003;46(3):359-64. Epub 2003/04/11. doi: 10.1007/s00125-003-1036-5. PubMed PMID: 12687334.

56. Lee DH, Jacobs DR, Jr., Gross M, Kiefe CI, Roseman J, Lewis CE, et al. Gamma-glutamyltransferase is a predictor of incident diabetes and hypertension: the Coronary Artery Risk Development in Young Adults (CARDIA) Study. Clinical chemistry. 2003;49(8):1358-66. Epub 2003/07/26. PubMed PMID: 12881453.

57. Lee SH, Kwon HS, Park YM, Ha HS, Jeong SH, Yang HK, et al. Predicting the development of diabetes using the product of triglycerides and glucose: the Chungju Metabolic Disease Cohort (CMC) study. PloS one. 2014;9(2):e90430. Epub 2014/03/04. doi: 10.1371/journal.pone.0090430. PubMed PMID: 24587359; PubMed Central PMCID: PMC3938726.

58. Ley SH, Harris SB, Connelly PW, Mamakeesick M, Gittelsohn J, Wolever TM, et al. Association of apolipoprotein B with incident type 2 diabetes in an aboriginal Canadian population. Clinical chemistry. 2010;56(4):666-70. Epub 2010/01/30. doi: 10.1373/clinchem.2009.136994. PubMed PMID: 20110448.

59. Lindsay RS, Krakoff J, Hanson RL, Bennett PH, Knowler WC. Gamma globulin levels predict type 2 diabetes in the Pima Indian population. Diabetes. 2001;50(7):1598-603. Epub 2001/06/26. PubMed PMID: 11423481.

60. Liu S, Tinker L, Song Y, Rifai N, Bonds DE, Cook NR, et al. A prospective study of inflammatory cytokines and diabetes mellitus in a multiethnic cohort of postmenopausal women. Archives of internal medicine. 2007;167(15):1676-85. Epub 2007/08/19. doi: 10.1001/archinte.167.15.1676. PubMed PMID: 17698692.

61. Luft VC, Schmidt MI, Pankow JS, Hoogeveen RC, Couper D, Heiss G, et al. Dipeptidyl peptidase IV and incident diabetes: the Atherosclerosis Risk in Communities (ARIC) study. Diabetes care. 2010;33(5):1109-11. Epub 2010/02/27. doi: 10.2337/dc09-1996. PubMed PMID: 20185737; PubMed Central PMCID: PMC2858185.

62. Lyssenko V, Jorgensen T, Gerwien RW, Hansen T, Rowe MW, McKenna MP, et al. Validation of a multi-marker model for the prediction of incident type 2 diabetes mellitus: combined results of the Inter99 and Botnia studies. Diabetes & vascular disease research. 2012;9(1):59-67. Epub 2011/11/08. doi: 10.1177/1479164111424762. PubMed PMID: 22058089.

63. Ma W, Wu JH, Wang Q, Lemaitre RN, Mukamal KJ, Djousse L, et al. Prospective association of fatty acids in the de novo lipogenesis pathway with risk of type 2 diabetes: the Cardiovascular Health Study. The American journal of clinical nutrition. 2015;101(1):153-63. Epub 2014/12/21. doi: 10.3945/ajcn.114.092601. PubMed PMID: 25527759; PubMed Central PMCID: PMC4266885.

64. Mainous AG, 3rd, King DE, Pearson WS, Garr DR. Is an elevated serum transferrin saturation associated with the development of diabetes? The Journal of family practice. 2002;51(11):933-6. Epub 2002/12/18. PubMed PMID: 12485546.

65. Mandel EI, Curhan GC, Hu FB, Taylor EN. Plasma bicarbonate and risk of type 2 diabetes mellitus. CMAJ : Canadian Medical Association journal = journal de l'Association medicale canadienne. 2012;184(13):E719-25. Epub 2012/07/25. doi: 10.1503/cmaj.120438. PubMed PMID: 22825995; PubMed Central PMCID: PMC3447038.

66. Mahendran Y, Agren J, Uusitupa M, Cederberg H, Vangipurapu J, Stancakova A, et al. Association of erythrocyte membrane fatty acids with changes in glycemia and risk of type 2 diabetes. The American journal of clinical nutrition. 2014;99(1):79-85. Epub 2013/10/25. doi: 10.3945/ajcn.113.069740. PubMed PMID: 24153340.

67. Marques-Vidal P, Schmid R, Bochud M, Bastardot F, von Kanel R, Paccaud F, et al. Adipocytokines, hepatic and inflammatory biomarkers and incidence of type 2 diabetes. the CoLaus study. PloS one. 2012;7(12):e51768. Epub 2012/12/20. doi: 10.1371/journal.pone.0051768. PubMed PMID: 23251619; PubMed Central PMCID: PMC3520903.

68. Mattila C, Knekt P, Mannisto S, Rissanen H, Laaksonen MA, Montonen J, et al. Serum 25-hydroxyvitamin D concentration and subsequent risk of type 2 diabetes. Diabetes care. 2007;30(10):2569-70. Epub 2007/07/14. doi: 10.2337/dc07-0292. PubMed PMID: 17626891.

69. McMullan CJ, Schernhammer ES, Rimm EB, Hu FB, Forman JP. Melatonin secretion and the incidence of type 2 diabetes. Jama. 2013;309(13):1388-96. Epub 2013/04/04. doi: 10.1001/jama.2013.2710. PubMed PMID: 23549584; PubMed Central PMCID: PMC3804914.

70. Meigs JB, Hu FB, Rifai N, Manson JE. Biomarkers of endothelial dysfunction and risk of type 2 diabetes mellitus. Jama. 2004;291(16):1978-86. Epub 2004/04/29. doi: 10.1001/jama.291.16.1978. PubMed PMID: 15113816.

71. Montonen J, Boeing H, Steffen A, Lehmann R, Fritsche A, Joost HG, et al. Body iron stores and risk of type 2 diabetes: results from the European Prospective Investigation into Cancer and Nutrition (EPIC)-Potsdam study. Diabetologia. 2012;55(10):2613-21. Epub 2012/07/04. doi: 10.1007/s00125-012-2633-y. PubMed PMID: 22752055; PubMed Central PMCID: PMC3433660.

72. Montonen J, Drogan D, Joost HG, Boeing H, Fritsche A, Schleicher E, et al. Estimation of the contribution of biomarkers of different metabolic pathways to risk of type 2 diabetes. European journal of epidemiology. 2011;26(1):29-38. Epub 2010/12/29. doi: 10.1007/s10654-010-9539-0. PubMed PMID: 21188480.

73. Mozaffarian D, de Oliveira Otto MC, Lemaitre RN, Fretts AM, Hotamisligil G, Tsai MY, et al. trans-Palmitoleic acid, other dairy fat biomarkers, and incident diabetes: the Multi-Ethnic Study of Atherosclerosis (MESA). The American journal of clinical nutrition. 2013;97(4):854-61. Epub 2013/02/15. doi: 10.3945/ajcn.112.045468. PubMed PMID: 23407305; PubMed Central PMCID: PMC3607658.

74. Mozaffarian D, Cao H, King IB, Lemaitre RN, Song X, Siscovick DS, et al. Trans-palmitoleic acid, metabolic risk factors, and new-onset diabetes in U.S. adults: a cohort study. Annals of internal medicine. 2010;153(12):790-9. Epub 2010/12/22. doi: 10.7326/0003-4819-153-12-201012210-00005. PubMed PMID: 21173413; PubMed Central PMCID: PMC3056495.

75. Mozaffarian D, Cao H, King IB, Lemaitre RN, Song X, Siscovick DS, et al. Circulating palmitoleic acid and risk of metabolic abnormalities and new-onset diabetes. The American journal of clinical nutrition. 2010;92(6):1350-8. Epub 2010/10/15. doi: 10.3945/ajcn.110.003970. PubMed PMID: 20943795; PubMed Central PMCID: PMC2980960.

76. Moller HJ, Frikke-Schmidt R, Moestrup SK, Nordestgaard BG, Tybjaerg-Hansen A. Serum soluble CD163 predicts risk of type 2 diabetes in the general population. Clinical chemistry. 2011;57(2):291-7. Epub 2010/11/26. doi: 10.1373/clinchem.2010.154724. PubMed PMID: 21106861.

77. Nan H, Qiao Q, Soderberg S, Pitkaniemi J, Zimmet P, Shaw J, et al. Serum uric acid and incident diabetes in Mauritian Indian and Creole populations. Diabetes research and clinical practice. 2008;80(2):321-7. Epub 2008/02/22. doi: 10.1016/j.diabres.2008.01.002. PubMed PMID: 18289714.

78. Neeland IJ, Turer AT, Ayers CR, Powell-Wiley TM, Vega GL, Farzaneh-Far R, et al. Dysfunctional adiposity and the risk of prediabetes and type 2 diabetes in obese adults. Jama. 2012;308(11):1150-9. Epub 2012/09/20. doi: 10.1001/2012.jama.11132. PubMed PMID: 22990274; PubMed Central PMCID: PMC3556508.

79. Ngarmukos C, Chailurkit LO, Chanprasertyothin S, Hengprasith B, Sritara P, Ongphiphadhanakul B. A reduced serum level of total osteocalcin in men predicts the development of diabetes in a long-term follow-up cohort. Clinical endocrinology. 2012;77(1):42-6. Epub 2011/09/16. doi: 10.1111/j.1365-2265.2011.04215.x. PubMed PMID: 21916911.

80. Nguyen QM, Srinivasan SR, Xu JH, Chen W, Hassig S, Rice J, et al. Elevated liver function enzymes are related to the development of prediabetes and type 2 diabetes in younger adults: the Bogalusa Heart Study. Diabetes care. 2011;34(12):2603-7. Epub 2011/09/29. doi: 10.2337/dc11-0919. PubMed PMID: 21953798; PubMed Central PMCID: PMC3220830.

81. Nguyen QM, Srinivasan SR, Xu JH, Chen W, Kieltyka L, Berenson GS. Utility of childhood glucose homeostasis variables in predicting adult diabetes and related cardiometabolic risk factors: the Bogalusa Heart Study. Diabetes care. 2010;33(3):670-5. Epub 2009/12/17. doi: 10.2337/dc09-1635. PubMed PMID: 20009096; PubMed Central PMCID: PMC2827529.

82. Nilsson SE, Fransson E, Brismar K. Relationship between serum progesterone concentrations and cardiovascular disease, diabetes, and mortality in elderly Swedish men and women: An 8-year prospective study. Gender medicine. 2009;6(3):433-43. Epub 2009/10/24. doi: 10.1016/j.genm.2009.09.011. PubMed PMID: 19850239.

83. Norberg M, Stenlund H, Lindahl B, Andersson C, Weinehall L, Hallmans G, et al. Components of metabolic syndrome predicting diabetes: no role of inflammation or dyslipidemia. Obesity (Silver Spring, Md). 2007;15(7):1875-85. Epub 2007/07/20. doi: 10.1038/oby.2007.222. PubMed PMID: 17636107.

84. Norberg M, Eriksson JW, Lindahl B, Andersson C, Rolandsson O, Stenlund H, et al. A combination of HbA1c, fasting glucose and BMI is effective in screening for individuals at risk of future type 2 diabetes: OGTT is not needed. Journal of internal medicine. 2006;260(3):263-71. Epub 2006/08/22. doi: 10.1111/j.1365-2796.2006.01689.x. PubMed PMID: 16918824.

85. Onat A, Can G, Yuksel H, Ayhan E, Dogan Y, Hergenc G. An algorithm to predict risk of type 2 diabetes in Turkish adults: contribution of C-reactive protein. Journal of endocrinological investigation. 2011;34(8):580-6. Epub 2010/11/03. doi: 10.3275/7323. PubMed PMID: 21042044.

86. Onat A, Hergenc G, Ayhan E, Ugur M, Can G. Impaired anti-inflammatory function of apolipoprotein A-II concentrations predicts metabolic syndrome and diabetes at 4 years follow-up in elderly Turks. Clinical chemistry and laboratory medicine : CCLM / FESCC. 2009;47(11):1389-94. Epub 2009/10/13. doi: 10.1515/cclm.2009.310. PubMed PMID: 19817643.

87. Onat A, Can G, Hergenc G, Yazici M, Karabulut A, Albayrak S. Serum apolipoprotein B predicts dyslipidemia, metabolic syndrome and, in women, hypertension and diabetes, independent of markers of central obesity and inflammation. International journal of obesity (2005). 2007;31(7):1119-25. Epub 2007/02/15. doi: 10.1038/sj.ijo.0803552. PubMed PMID: 17299378.

88. Patel PS, Cooper AJ, O'Connell TC, Kuhnle GG, Kneale CK, Mulligan AM, et al. Serum carbon and nitrogen stable isotopes as potential biomarkers of dietary intake and their relation with incident type 2 diabetes: the EPIC-Norfolk study. The American journal of clinical nutrition. 2014;100(2):708-18. Epub 2014/07/06. doi: 10.3945/ajcn.113.068577. PubMed PMID: 24990425; PubMed Central PMCID: PMC4095667.

89. Pradhan AD, Rifai N, Buring JE, Ridker PM. Hemoglobin A1c predicts diabetes but not cardiovascular disease in nondiabetic women. The American journal of medicine. 2007;120(8):720-7. Epub 2007/08/07. doi: 10.1016/j.amjmed.2007.03.022. PubMed PMID: 17679132; PubMed Central PMCID: PMC2585540.

90. Pradhan AD, Manson JE, Meigs JB, Rifai N, Buring JE, Liu S, et al. Insulin, proinsulin, proinsulin:insulin ratio, and the risk of developing type 2 diabetes mellitus in women. The American journal of medicine. 2003;114(6):438-44. Epub 2003/05/03. PubMed PMID: 12727576.

91. Pradhan AD, Manson JE, Rifai N, Buring JE, Ridker PM. C-reactive protein, interleukin 6, and risk of developing type 2 diabetes mellitus. Jama. 2001;286(3):327-34. Epub 2001/07/24. PubMed PMID: 11466099.

92. Rathmann W, Kowall B, Heier M, Herder C, Holle R, Thorand B, et al. Prediction models for incident type 2 diabetes mellitusin the older population: KORA S4/F4 cohort study. Diabetic medicine : a journal of the British Diabetic Association. 2010;27(10):1116-23. Epub 2010/09/22. doi: 10.1111/j.1464-5491.2010.03065.x. PubMed PMID: 20854378.

93. Raynor LA, Pankow JS, Duncan BB, Schmidt MI, Hoogeveen RC, Pereira MA, et al. Novel risk factors and the prediction of type 2 diabetes in the Atherosclerosis Risk in Communities (ARIC) study. Diabetes care. 2013;36(1):70-6. Epub 2012/08/31. doi: 10.2337/dc12-0609. PubMed PMID: 22933437; PubMed Central PMCID: PMC3526210.

94. Rhee EJ, Seo MH, Jeon WS, Won HK, Choi JH, Park SE, et al. The association of baseline adipocytokine levels with glycemic progression in nondiabetic Korean adults in 4 years of follow-up. Diabetes research and clinical practice. 2012;98(3):501-7. Epub 2012/10/17. doi: 10.1016/j.diabres.2012.09.022. PubMed PMID: 23068962.

95. Rhee EP, Cheng S, Larson MG, Walford GA, Lewis GD, McCabe E, et al. Lipid profiling identifies a triacylglycerol signature of insulin resistance and improves diabetes prediction in humans. The Journal of clinical investigation. 2011;121(4):1402-11. Epub 2011/03/16. doi: 10.1172/jci44442. PubMed PMID: 21403394; PubMed Central PMCID: PMC3069773.

96. Rolandsson O, Hagg E, Nilsson M, Hallmans G, Mincheva-Nilsson L, Lernmark A. Prediction of diabetes with body mass index, oral glucose tolerance test and islet cell autoantibodies in a regional population. Journal of internal medicine. 2001;249(4):279-88. Epub 2001/04/12. PubMed PMID: 11298847.

97. Sahakyan K, Lee KE, Shankar A, Klein R. Serum cystatin C and the incidence of type 2 diabetes mellitus. Diabetologia. 2011;54(6):1335-40. Epub 2011/03/08. doi: 10.1007/s00125-011-2096-6. PubMed PMID: 21380596; PubMed Central PMCID: PMC3290654.

98. Salomaa V, Havulinna A, Saarela O, Zeller T, Jousilahti P, Jula A, et al. Thirty-one novel biomarkers as predictors for clinically incident diabetes. PloS one. 2010;5(4):e10100. Epub 2010/04/17. doi: 10.1371/journal.pone.0010100. PubMed PMID: 20396381; PubMed Central PMCID: PMC2852424.

99. Santaren ID, Watkins SM, Liese AD, Wagenknecht LE, Rewers MJ, Haffner SM, et al. Serum pentadecanoic acid (15:0), a short-term marker of dairy food intake, is inversely associated with incident type 2 diabetes and its underlying disorders. The American journal of clinical nutrition. 2014;100(6):1532-40. Epub 2014/11/21. doi: 10.3945/ajcn.114.092544. PubMed PMID: 25411288; PubMed Central PMCID: PMC4232018.

100. Sattar N, Murray HM, Welsh P, Blauw GJ, Buckley BM, de Craen AJ, et al. Are elevated circulating intercellular adhesion molecule 1 levels more strongly predictive of diabetes than vascular risk? Outcome of a prospective study in the elderly. Diabetologia. 2009;52(2):235-9. Epub 2008/11/26. doi: 10.1007/s00125-008-1217-3. PubMed PMID: 19030842.

101. Schulze MB, Solomon CG, Rifai N, Cohen RM, Sparrow J, Hu FB, et al. Hyperproinsulinaemia and risk of Type 2 diabetes mellitus in women. Diabetic medicine : a journal of the British Diabetic Association. 2005;22(9):1178-84. Epub 2005/08/20. doi: 10.1111/j.1464-5491.2005.01585.x. PubMed PMID: 16108846.

102. Schafer AL, Napoli N, Lui L, Schwartz AV, Black DM, Study of Osteoporotic F. Serum 25-hydroxyvitamin D concentration does not independently predict incident diabetes in older women. Diabetic medicine : a journal of the British Diabetic Association. 2014;31(5):564-9. Epub 2013/12/05. doi: 10.1111/dme.12368. PubMed PMID: 24299116; PubMed Central PMCID: PMC3988213.

103. Selvin E, Rawlings AM, Grams M, Klein R, Sharrett AR, Steffes M, et al. Fructosamine and glycated albumin for risk stratification and prediction of incident diabetes and microvascular complications: a prospective cohort analysis of the Atherosclerosis Risk in Communities (ARIC) study. The lancet Diabetes & endocrinology. 2014;2(4):279-88. Epub 2014/04/08. doi: 10.1016/s2213-8587(13)70199-2. PubMed PMID: 24703046; PubMed Central PMCID: PMC4212648.

104. Schottker B, Herder C, Rothenbacher D, Perna L, Muller H, Brenner H. Serum 25-hydroxyvitamin D levels and incident diabetes mellitus type 2: a competing risk analysis in a large population-based cohort of older adults. European journal of epidemiology. 2013;28(3):267-75. Epub 2013/01/29. doi: 10.1007/s10654-013-9769-z. PubMed PMID: 23354985.

105. Schottker B, Raum E, Rothenbacher D, Muller H, Brenner H. Prognostic value of haemoglobin A1c and fasting plasma glucose for incident diabetes and implications for screening. European journal of epidemiology. 2011;26(10):779-87. Epub 2011/09/29. doi: 10.1007/s10654-011-9619-9. PubMed PMID: 21947790.

106. Shlomai A, Kariv R, Leshno M, Beth-or A, Sheinberg B, Halpern Z. Large-scale population analysis reveals an extremely low threshold for "non-healthy" alanine aminotransferase that predicts diabetes mellitus. Journal of gastroenterology and hepatology. 2010;25(10):1687-91. Epub 2010/10/01. doi: 10.1111/j.1440-1746.2010.06369.x. PubMed PMID: 20880180.

107. Sluijs I, Beulens JW, van der AD, Spijkerman AM, Schulze MB, van der Schouw YT. Plasma uric acid is associated with increased risk of type 2 diabetes independent of diet and metabolic risk factors. The Journal of nutrition. 2013;143(1):80-5. Epub 2012/11/23. doi: 10.3945/jn.112.167221. PubMed PMID: 23173177.

108. Song Y, Manson JE, Tinker L, Rifai N, Cook NR, Hu FB, et al. Circulating levels of endothelial adhesion molecules and risk of diabetes in an ethnically diverse cohort of women. Diabetes. 2007;56(7):1898-904. Epub 2007/03/29. doi: 10.2337/db07-0250. PubMed PMID: 17389327; PubMed Central PMCID: PMC1952236.

109. Song Y, Manson JE, Tinker L, Howard BV, Kuller LH, Nathan L, et al. Insulin sensitivity and insulin secretion determined by homeostasis model assessment and risk of diabetes in a multiethnic cohort of women: the Women's Health Initiative Observational Study. Diabetes care. 2007;30(7):1747-52. Epub 2007/05/01. doi: 10.2337/dc07-0358. PubMed PMID: 17468352; PubMed Central PMCID: PMC1952235.

110. Soulimane S, Simon D, Shaw J, Witte D, Zimmet P, Vol S, et al. HbA1c, fasting plasma glucose and the prediction of diabetes: Inter99, AusDiab and D.E.S.I.R. Diabetes research and clinical practice. 2012;96(3):392-9. Epub 2011/07/12. doi: 10.1016/j.diabres.2011.06.003. PubMed PMID: 21741107.

111. Stefan N, Fritsche A, Weikert C, Boeing H, Joost HG, Haring HU, et al. Plasma fetuin-A levels and the risk of type 2 diabetes. Diabetes. 2008;57(10):2762-7. Epub 2008/07/18. doi: 10.2337/db08-0538. PubMed PMID: 18633113; PubMed Central PMCID: PMC2551687.

112. Stefan N, Sun Q, Fritsche A, Machann J, Schick F, Gerst F, et al. Impact of the adipokine adiponectin and the hepatokine fetuin-A on the development of type 2 diabetes: prospective cohort- and cross-sectional phenotyping studies. PloS one. 2014;9(3):e92238. Epub 2014/03/20. doi: 10.1371/journal.pone.0092238. PubMed PMID: 24643166; PubMed Central PMCID: PMC3958485.

113. Steffen BT, Steffen LM, Zhou X, Ouyang P, Weir NL, Tsai MY. n-3 Fatty acids attenuate the risk of diabetes associated with elevated serum nonesterified fatty acids: the multi-ethnic study of atherosclerosis. Diabetes care. 2015;38(4):575-80. Epub 2015/01/13. doi: 10.2337/dc14-1919. PubMed PMID: 25573885; PubMed Central PMCID: PMC4370329.

114. Stranges S, Rafalson LB, Dmochowski J, Rejman K, Tracy RP, Trevisan M, et al. Additional contribution of emerging risk factors to the prediction of the risk of type 2 diabetes: evidence from the Western New York Study. Obesity (Silver Spring, Md). 2008;16(6):1370-6. Epub 2008/03/22. doi: 10.1038/oby.2008.59. PubMed PMID: 18356828.

115. Sun L, Zong G, Pan A, Ye X, Li H, Yu Z, et al. Elevated plasma ferritin is associated with increased incidence of type 2 diabetes in middle-aged and elderly Chinese adults. The Journal of nutrition. 2013;143(9):1459-65. Epub 2013/08/02. doi: 10.3945/jn.113.177808. PubMed PMID: 23902953.

116. Sun Q, van Dam RM, Meigs JB, Franco OH, Mantzoros CS, Hu FB. Leptin and soluble leptin receptor levels in plasma and risk of type 2 diabetes in U.S. women: a prospective study. Diabetes. 2010;59(3):611-8. Epub 2009/12/05. doi: 10.2337/db09-1343. PubMed PMID: 19959759; PubMed Central PMCID: PMC2828671.

117. Tabak AG, Jokela M, Akbaraly TN, Brunner EJ, Kivimaki M, Witte DR. Trajectories of glycaemia, insulin sensitivity, and insulin secretion before diagnosis of type 2 diabetes: an analysis from the Whitehall II study. Lancet (London, England). 2009;373(9682):2215-21. Epub 2009/06/12. doi: 10.1016/s0140-6736(09)60619-x. PubMed PMID: 19515410; PubMed Central PMCID: PMC2726723.

118. Thorand B, Zierer A, Baumert J, Meisinger C, Herder C, Koenig W. Associations between leptin and the leptin / adiponectin ratio and incident Type 2 diabetes in middle-aged men and women: results from the MONICA / KORA Augsburg study 1984-2002. Diabetic medicine : a journal of the British Diabetic Association. 2010;27(9):1004-11. Epub 2010/08/21. doi: 10.1111/j.1464-5491.2010.03043.x. PubMed PMID: 20722673.

119. Thorand B, Baumert J, Herder C, Meisinger C, Koenig W. Soluble thrombomodulin as a predictor of type 2 diabetes: results from the MONICA/KORA Augsburg case-cohort study, 1984-1998. Diabetologia. 2007;50(3):545-8. Epub 2006/12/30. doi: 10.1007/s00125-006-0568-x. PubMed PMID: 17195062.

120. Thorand B, Baumert J, Kolb H, Meisinger C, Chambless L, Koenig W, et al. Sex differences in the prediction of type 2 diabetes by inflammatory markers: results from the MONICA/KORA Augsburg case-cohort study, 1984-2002. Diabetes care. 2007;30(4):854-60. Epub 2007/03/30. doi: 10.2337/dc06-1693. PubMed PMID: 17392546.

121. Thorand B, Baumert J, Chambless L, Meisinger C, Kolb H, Doring A, et al. Elevated markers of endothelial dysfunction predict type 2 diabetes mellitus in middle-aged men and women from the general population. Arteriosclerosis, thrombosis, and vascular biology. 2006;26(2):398-405. Epub 2005/12/03. doi: 10.1161/01.ATV.0000198392.05307.aa. PubMed PMID: 16322530.

122. Thorand B, Kolb H, Baumert J, Koenig W, Chambless L, Meisinger C, et al. Elevated levels of interleukin-18 predict the development of type 2 diabetes: results from the MONICA/KORA Augsburg Study, 1984-2002. Diabetes. 2005;54(10):2932-8. Epub 2005/09/28. PubMed PMID: 16186395.

123. Thorand B, Lowel H, Schneider A, Kolb H, Meisinger C, Frohlich M, et al. C-reactive protein as a predictor for incident diabetes mellitus among middle-aged men: results from the MONICA Augsburg cohort study, 1984-1998. Archives of internal medicine. 2003;163(1):93-9. Epub 2003/01/14. PubMed PMID: 12523922.

124. Urdea M, Kolberg J, Wilber J, Gerwien R, Moler E, Rowe M, et al. Validation of a multimarker model for assessing risk of type 2 diabetes from a five-year prospective study of 6784 Danish people (Inter99). Journal of diabetes science and technology. 2009;3(4):748-55. Epub 2010/02/11. PubMed PMID: 20144324; PubMed Central PMCID: PMC2769937.

125. Vaccaro O, Cuomo V, Trevisan M, Cirillo M, Panarelli W, Laurenzi M, et al. Enhanced Na-Li countertransport: a marker of inherited susceptibility to type 2 diabetes. International journal of epidemiology. 2005;34(5):1123-8. Epub 2005/08/10. doi: 10.1093/ije/dyi160. PubMed PMID: 16087689.

126. Vigo A, Duncan BB, Schmidt MI, Couper D, Heiss G, Pankow JS, et al. Glutamic acid decarboxylase antibodies are indicators of the course, but not of the onset, of diabetes in middle-aged adults: the Atherosclerosis Risk in Communities Study. Brazilian journal of medical and biological research = Revista brasileira de pesquisas medicas e biologicas / Sociedade Brasileira de Biofisica [et al]. 2007;40(7):933-41. Epub 2007/07/27. PubMed PMID: 17653446; PubMed Central PMCID: PMC2423490.

127. Vozarova B, Stefan N, Lindsay RS, Saremi A, Pratley RE, Bogardus C, et al. High alanine aminotransferase is associated with decreased hepatic insulin sensitivity and predicts the development of type 2 diabetes. Diabetes. 2002;51(6):1889-95. Epub 2002/05/29. PubMed PMID: 12031978.

128. Vozarova B, Weyer C, Lindsay RS, Pratley RE, Bogardus C, Tataranni PA. High white blood cell count is associated with a worsening of insulin sensitivity and predicts the development of type 2 diabetes. Diabetes. 2002;51(2):455-61. Epub 2002/01/29. PubMed PMID: 11812755.

129. Wang T, Bi Y, Xu M, Huang Y, Xu Y, Li X, et al. Serum uric acid associates with the incidence of type 2 diabetes in a prospective cohort of middle-aged and elderly Chinese. Endocrine. 2011;40(1):109-16. Epub 2011/03/25. doi: 10.1007/s12020-011-9449-2. PubMed PMID: 21431449.

130. Wang TJ, Ngo D, Psychogios N, Dejam A, Larson MG, Vasan RS, et al. 2-Aminoadipic acid is a biomarker for diabetes risk. The Journal of clinical investigation. 2013;123(10):4309-17. Epub 2013/10/05. doi: 10.1172/jci64801. PubMed PMID: 24091325; PubMed Central PMCID: PMC3784523.

131. Wang TJ, Larson MG, Vasan RS, Cheng S, Rhee EP, McCabe E, et al. Metabolite profiles and the risk of developing diabetes. Nature medicine. 2011;17(4):448-53. Epub 2011/03/23. doi: 10.1038/nm.2307. PubMed PMID: 21423183; PubMed Central PMCID: PMC3126616.

132. Wang Z, Hoy WE. Albuminuria as a marker of the risk of developing type 2 diabetes in non-diabetic Aboriginal Australians. International journal of epidemiology. 2006;35(5):1331-5. Epub 2006/06/20. doi: 10.1093/ije/dyl115. PubMed PMID: 16782970.

133. Wang-Sattler R, Yu Z, Herder C, Messias AC, Floegel A, He Y, et al. Novel biomarkers for pre-diabetes identified by metabolomics. Molecular systems biology. 2012;8:615. Epub 2012/09/27. doi: 10.1038/msb.2012.43. PubMed PMID: 23010998; PubMed Central PMCID: PMC3472689.

134. Wannamethee SG, Papacosta O, Whincup PH, Thomas MC, Carson C, Lawlor DA, et al. The potential for a two-stage diabetes risk algorithm combining non-laboratory-based scores with subsequent routine non-fasting blood tests: results from prospective studies in older men and women. Diabetic medicine : a journal of the British Diabetic Association. 2011;28(1):23-30. Epub 2010/12/21. doi: 10.1111/j.1464-5491.2010.03171.x. PubMed PMID: 21166842.

135. Wannamethee SG, Sattar N, Rumley A, Whincup PH, Lennon L, Lowe GD. Tissue plasminogen activator, von Willebrand factor, and risk of type 2 diabetes in older men. Diabetes care. 2008;31(5):995-1000. Epub 2008/02/01. doi: 10.2337/dc07-1569. PubMed PMID: 18235054.

136. Welsh P, Murray HM, Buckley BM, de Craen AJ, Ford I, Jukema JW, et al. Leptin predicts diabetes but not cardiovascular disease: results from a large prospective study in an elderly population. Diabetes care. 2009;32(2):308-10. Epub 2008/11/13. doi: 10.2337/dc08-1458. PubMed PMID: 19001191; PubMed Central PMCID: PMC2628699.

137. Wu JH, Micha R, Imamura F, Pan A, Biggs ML, Ajaz O, et al. Omega-3 fatty acids and incident type 2 diabetes: a systematic review and meta-analysis. The British journal of nutrition. 2012;107 Suppl 2:S214-27. Epub 2012/05/25. doi: 10.1017/s0007114512001602. PubMed PMID: 22591895; PubMed Central PMCID: PMC3744862.

138. Xu Y, Xu M, Huang Y, Wang T, Li M, Wu Y, et al. Elevated serum gamma-glutamyltransferase predicts the development of impaired glucose metabolism in middle-aged and elderly Chinese. Endocrine. 2011;40(2):265-72. Epub 2011/04/28. doi: 10.1007/s12020-011-9468-z. PubMed PMID: 21523521.

139. Zheng T, Gao Y, Baskota A, Chen T, Ran X, Tian H. Increased plasma DPP4 activity is predictive of prediabetes and type 2 diabetes onset in Chinese over a four-year period: result from the China National Diabetes and Metabolic Disorders Study. The Journal of clinical endocrinology and metabolism. 2014;99(11):E2330-4. Epub 2014/07/17. doi: 10.1210/jc.2014-1480. PubMed PMID: 25029421.
