# Supplementary material for: A Systematic Review of Biomarkers and Risk of Incident Type 2 Diabetes: An Overview of Epidemiological, Prediction and Aetiological Research Literature
Source: PLoS One. 2016 Oct 27;11(10):e0163721. doi: 10.1371/journal.pone.0163721 (PMC5082867; doi:10.1371/journal.pone.0163721)
Supplement: S2 Table — (DOC) [file pone.0163721.s006.doc]

**S2 Table. Excluded Studies.**

| **Title** | **Description** | **Details** | **Short details** | **PMID** | **Exclusion criteria** |
| --- | --- | --- | --- | --- | --- |
| Asymmetric dimethylarginine, cortisol/cortisone ratio, and C-peptide: markers for diabetes and cardiovascular risk? | Anderson JL, et al | Am Heart J. 2007 Jan;153(1):67-73. | Am Heart J. 2007 | 17174641 | cross-sectional for T2D |
| Estimating the mediating effect of different biomarkers on the relation of alcohol consumption with the risk of type 2 diabetes. | Beulens JW,et al | Ann Epidemiol. 2013 Apr;23(4):193-7. doi: 10.1016/j.annepidem.2012.12.014. Epub 2013 Jan 29. | Ann Epidemiol. 2013 | 23375342 | Not specific biomarker |
| Alcohol consumption, mediating biomarkers, and risk of type 2 diabetes among middle-aged women. | Beulens JW, et al | Diabetes Care. 2008 Oct;31(10):2050-5. doi: 10.2337/dc08-0814. Epub 2008 Jul 15. | Diabetes Care. 2008 | 18628567 | Not specific biomarker |
| Low serum concentration of sulfatide and presence of sulfated lactosylceramid are associated with Type 2 diabetes. The Skaraborg Project. | Buschard K, et al | Diabet Med. 2005 Sep;22(9):1190-8. | Diabet Med. 2005 | 16108848 | cross-sectional for T2D |
| Glucose and non-glucose predictors of future onset of type 2 diabetes in newly diagnosed essential hypertensives. | Fong MC, et al | J Chin Med Assoc. 2009 Nov;72(11):564-72. doi: 10.1016/S1726-4901(09)70431-9. | J Chin Med Assoc. 2009 | 19948433 | small study, hypertensive sample |
| Associations of sex hormone-binding globulin and testosterone with diabetes among men and women (the Saku Diabetes study): a case control study. | Goto A, et al | Cardiovasc Diabetol. 2012 Oct 16;11:130. doi: 10.1186/1475-2840-11-130. | Cardiovasc Diabetol. 2012 | 23066943 | cross-sectional for T2D |
| Prediction of type 2 diabetes mellitus with alternative definitions of the metabolic syndrome: the Insulin Resistance Atherosclerosis Study. | Hanley AJ, et al | Circulation. 2005 Dec 13;112(24):3713-21. | Circulation. 2005 | 16344402 | not reporting measure of association |
| Urinary chiro- and myo-inositol levels as a biological marker for type 2 diabetes mellitus. | Hong JH, et al | Dis Markers. 2012;33(4):193-9. doi: 10.3233/DMA-2012-0925. | Dis Markers. 2012 | 22960342 | cross-sectional for T2D |
| Evaluation of various biomarkers as potential mediators of the association between coffee consumption and incident type 2 diabetes in the EPIC-Potsdam Study. | Jacobs S, et al | Am J Clin Nutr. 2014 Sep;100(3):891-900. doi: 10.3945/ajcn.113.080317. Epub 2014 Jul 23. | Am J Clin Nutr. 2014 | 25057154 | not reporting measure of association |
| Do non-glycaemic markers add value to plasma glucose and hemoglobin a1c in predicting diabetes? Yuport health checkup center study. | Kashima S, et al | PLoS One. 2013 Jun 20;8(6):e66899. doi: 10.1371/journal.pone.0066899. Print 2013. | PLoS One. 2013 | 23818970 | not reporting measure of association |
| Growth differentiation factor 15 predicts future insulin resistance and impaired glucose control in obese nondiabetic individuals: results from the XENDOS trial. | Kempf T, et al | Eur J Endocrinol. 2012 Nov;167(5):671-8. doi: 10.1530/EJE-12-0466. Epub 2012 Aug 23. | Eur J Endocrinol. 2012 | 22918303 | cross-sectional for T2D |
| The effect of insulin on net lipid oxidation predicts worsening of insulin resistance and development of type 2 diabetes mellitus. | Koska J, et al | Am J Physiol Endocrinol Metab. 2007 Jul;293(1):E264-9. | Am J Physiol Endocrinol Metab. 2007 | 17616607 | not reporting measure of association |
| Random glucose is useful for individual prediction of type 2 diabetes: results of the Study of Health in Pomerania (SHIP). | Kowall B, et al | Prim Care Diabetes. 2013 Apr;7(1):25-31. doi: 10.1016/j.pcd.2012.12.001. Epub 2012 Dec 30. | Prim Care Diabetes. 2013 | 23280257 | not reporting measure of association |
| Liver aminotransferases and risk of incident type 2 diabetes: a systematic review and meta-analysis. | Kunutsor SK, et al | Am J Epidemiol. 2013 Jul 15;178(2):159-71. doi: 10.1093/aje/kws469. Epub 2013 May 31. Review. | Am J Epidemiol. 2013 | 23729682 | not reporting measure of association |
| Associations between interleukin-1 (IL-1) gene variations or IL-1 receptor antagonist levels and the development of type 2 diabetes. | Luotola K, et al | J Intern Med. 2011 Mar;269(3):322-32. doi: 10.1111/j.1365-2796.2010.02294.x. | J Intern Med. 2011 | 21205020 | Studied MetS population |
| Biomarkers for type 2 diabetes and impaired fasting glucose using a nontargeted metabolomics approach. | Menni C, et al | Diabetes. 2013 Dec;62(12):4270-6. doi: 10.2337/db13-0570. Epub 2013 Jul 24. | Diabetes. 2013 | 23884885 | Cross-sectional |
| Low serum amylase in association with metabolic syndrome and diabetes: A community-based study. | Nakajima K, et al | Cardiovasc Diabetol. 2011 Apr 17;10:34. doi: 10.1186/1475-2840-10-34. | Cardiovasc Diabetol. 2011 | 21496338 | Cross-sectional |
| Evaluation of the combined use of adiponectin and C-reactive protein levels as biomarkers for predicting the deterioration in glycaemia after a median of 5.4 years. | Ong KL, et al | Diabetologia. 2011 Oct;54(10):2552-60. doi: 10.1007/s00125-011-2227-0. Epub 2011 Jul 5. | Diabetologia. 2011 | 21727999 | No estimate for T2D |
| Chronic kidney disease, insulin resistance, and incident diabetes in older adults. | Pham H, et al | Clin J Am Soc Nephrol. 2012 Apr;7(4):588-94. doi: 10.2215/CJN.11861111. Epub 2012 Mar 1. | Clin J Am Soc Nephrol. 2012 | 22383749 | Not specific biomarker |
| Use of multiple metabolic and genetic markers to improve the prediction of type 2 diabetes: the EPIC-Potsdam Study. | Schulze MB, et al | Diabetes Care. 2009 Nov;32(11):2116-9. doi: 10.2337/dc09-0197. Epub 2009 Aug 31. | Diabetes Care. 2009 | 19720844 | not reporting measure of association |
| Relationship between urinary bisphenol A levels and diabetes mellitus. | Shankar A, Teppala S. | J Clin Endocrinol Metab. 2011 Dec;96(12):3822-6. doi: 10.1210/jc.2011-1682. Epub 2011 Sep 28. | J Clin Endocrinol Metab. 2011 | 21956417 | cross-sectional for T2D |
| High plasma immunoglobulin (Ig) A and low IgG antibody titers to oxidized low-density lipoprotein are associated with markers of glucose metabolism. | SÃ¤mpi M, et al | J Clin Endocrinol Metab. 2010 May;95(5):2467-75. doi: 10.1210/jc.2009-1858. Epub 2010 Mar 23. | J Clin Endocrinol Metab. 2010 | 20332251 | cross-sectional for T2D |
| Serum fructosamine as a marker of 5-year risk of developing diabetes mellitus in patients exhibiting stress hyperglycaemia. | Wahid ST, et al | Diabet Med. 2002 Jul;19(7):543-8. | Diabet Med. 2002 | 12099956 | Study restricted to patients attending ER |
| The metabolic syndrome defined by factor analysis and incident type 2 diabetes in a chinese population with high postprandial glucose. | Wang JJ, et al | Diabetes Care. 2004 Oct;27(10):2429-37. | Diabetes Care. 2004 | 15451912 | Not specific biomarker |
| Increased C-reactive protein is associated with future development of diabetes mellitus in essential hypertensive patients. | Weng CM, et al | Heart Vessels. 2010 Sep;25(5):386-91. doi: 10.1007/s00380-009-1218-2. Epub 2010 Jul 31. | Heart Vessels. 2010 | 20676960 | small study, hypertensive sample |
