# Supplementary material for: A Systematic Review of Biomarkers and Risk of Incident Type 2 Diabetes: An Overview of Epidemiological, Prediction and Aetiological Research Literature
Source: PLoS One. 2016 Oct 27;11(10):e0163721. doi: 10.1371/journal.pone.0163721 (PMC5082867; doi:10.1371/journal.pone.0163721)
Supplement: S3 Table — (DOC) [file pone.0163721.s007.doc]

**S3 Table. 167 Biomarkers for Risk of Incident T2D.**

| **Category** | **Biomarker** | **Type** | **Study/Year** | **Biomarker level** | **Biomarker assay*** | **Measures of association for T2D risk** | **Type of association** | **P value** | **Multivariable adjustment**† |
| --- | --- | --- | --- | --- | --- | --- | --- | --- | --- |
| Adipokine | Adiponectin | Novel | Choi, et al/ 2004 | 13.8±1.76 μg/ml | RIA | OR, 0.80(0.6-0.99) | 10 μg/ml | 0.02 | 2 |
|  |  |  | Daimon, et al/ 2003 | 9.06±2.41 μg/ml | ELISA | OR, 0.766 | 0.1 log μg/ml | 0.029 | 1,2,3 |
|  |  |  | Fagerberg, et al/ 2011 | 10.35 (7.6) (T2D) vs 15.6 (9.91) (non-case) μg/ml | ELISA | OR, 0.22 (0.07-0.69) | Tertile 3 | 0.009 | 3 |
|  |  |  | Heidemann, et al/ 2008 | 11.4 (7.6–16.3) (T2D) vs 17.8 (12.9–22.7) (control) μg/ml | ELISA | OR, 0.17 (0.12–0.25) | Quintile 5 | <0.001 | 1,2 |
|  |  |  | Herder, et al/ 2011 | mean.geom (antilog se) 9.37 (1.02) vs 11.52 (1.01) μg/ml | ELISA | HR, 0.33 (0.23-0.47) | SD | <0.001 | 1,2,3 |
|  |  |  | Lyssenko et al. 2012 | Median (IQR), inter99 controls: 8.4 (6.6–10.8) mg/dL | ELISA | OR, 0.69 (0.48–0.95) | SD | NR | None |
|  |  |  | Montonen, et al/ 2011 | 5.65±2.64 (T2D) vs 8.18±3.96 (non-case) μg/ml | ELISA | RR 0.18 (0.12-0.28) | Quintile 5 | <0.001 | 1,2 |
|  |  |  | Rathmann, et al/ 2010 | 6.4 (4.9-9.9) (T2D) vs 9.2 (6.5-12.5) (non-case) μg/mL | RIA | Change in C-index: from 0.844 to 0.846 | log | NA | 1,2,3 |
|  |  |  | Raynor et al./2013 | 6.9/9.3 μg/ml | ELISA | Change in C-index: from 0.8607 to 0.8624 | SD | <0.0001 | 1,2,3 |
|  |  |  | Stranges et al./2008 | 12.66 ng/ml | RIA | OR, t1: 1.00, t2: 0.72 (0.31-1.67), t3: 0.28 (0.10-0.82) | Tertile 3 | 0.021 | 1 |
|  |  |  | Thorand et al./2010 | M: 8.16 μg/l, F: 11.22 μg/l | ELISA | HR, T1: 1.0, T2: 2.52 (1.84-3.45), T3: 4.72 (3.47-6.40) | Tertile 3 | <0.001 | 1 |
|  |  |  | Krakoff J et al/ 2003 | Median controls (IQR): 5.3 (4.3–6.3) (ug/ml) | ELISA | 0.63 (0.41-0.98) | SD | 0.04 | 1,2,3 |
|  |  |  | Salomaa, et al/ 2010 | 5602.3 (3645.0-8838.1) (ng/ml) | ELISA | NR (only graphically shown) | SD | <0.001 | 1,2,3 |
|  | Adiponectin high-molecular-weight |  | Heidemann, et al/ 2008 | 3.6 (2.2–5.8) (T2D) vs 6.6 (4.4–9.8) (control) μg/ml | ELISA | OR 0.10 (0.06–0.15) | Quintile 5 | <0.001 | 1,2 |
|  | Fatty acid-binding protein-4 (FABP-4) | Novel | Rhee, et al/2012 | 10.55±5.5 ng/ml | ELISA | OR, 1.943 (1.151, 3.279), 4.483 (2.170, 9.259) | log | 0.013, <0.001 | 1,2,3 |
|  | Leptin | Novel | Herder, et al/ 2011 | mean.geom (antilog se) 14.26 (1.05) (T2D) vs 9.11 (1.03) (non-case) μg/l | ELISA | HR 1.29 (0.97-1.73) | SD | 0.079 | 1,2,3 |
|  |  |  | Norberg, et al/ 2007 | M: 8.1, F: 17.4 ng/ml | RIA | OR, M: 5.8 (3.3-10.3), F: 5.5 (2.8-1.1) | NR | <0.001 | 1 |
|  |  |  | Raynor et al./2013 | 26.1/19.4 ng/ml | ELISA | Change in C-index: from 0.8607 to 0.8617 | SD | 0.04 |  |
|  |  |  | Sun et al./2010 | 29.2±13.8 ng/ml | RIA | OR, q1: 1.0, q2: 0.76 (0.55-1.04), q3: 0.90 (0.67-1.21), q4: 0.95 (0.71-1.28), q5: 0.82 (0.62-1.10) | Quartile | 0.46 | 1,2 |
|  |  |  | Thorand et al./2010 | M: 7.97 μg/l, F: 28.85 μg/l | ELISA | HR, T1:1.0, T2: 2.60 (1.82-3.71), T3: 5.18 (3.69-7.29) | Tertile | <0.001 | 1 |
|  |  |  | Welsh, et al/ 2009 | 12.4 ng/ml | RIA | HR, M: 2.75 (2.14-3.52), F: 1.54 (1.22-1.94) | log | M: <0.0001, F; 0.0003 | 1,2 |
|  |  |  | Salomaa 2010 | 8120.3 (4128.0-16268.0) (pg/ml) | ELISA | NR (only graphically shown) | SD | 0.4581 | 1,2,3 |
|  | Leptin/adiponectin ratio | Novel | Thorand et al./2010 | M: 0.98, F: 2.57 | ELISA | HR, T1: 1.0, T2: 2.98 (2.03-4.38), T3: 7.87 (5.74-11.31) | Tertile | <0.001 | 1 |
|  | Resistin | Novel | Heidemann, et al 2008 | 16.4 (12.4–23.6) (T2D) vs 15.1 (11.5–21.2) (control) ng/ml | ELISA | OR 1.28 (0.93–1.76) | Quintile 5 | 0.15 | 1,2 |
|  | Retinol-binding protein-4 (RBP-4) | Novel | Rhee EJ et al./2012 | 50.0±56.3 μg/ml | ELISA | NR (not significant) | log | NR (not significant) | 1, 2 |
|  | Soluble leptin receptor (sOB-R) | Novel | Sun et al./2010 | 28.0±8.2 ng/ml | RIA | OR, q1: 1.0, q2: 0.73 (0.55-0.96), q3: 0.51 (0.38-0.68), q4: 0.42 (0.31-0.57), q5: 0.39 (0.28-0.54) | Quartile | <0.0001 | 1,2 |
| Blood cells | White bloodcell count (WBC) | Clinical | Raynor et al./2013 | 6.4/6.0 (1,000/mm3) | Coulter counter | Change in C-index: from 0.8411 to 0.8430 | SD | <0.0001 | 1,2,3 |
|  |  |  | Stranges et al./2008 | 6.67 x 10^9^/l | Coulter counter | OR, t1: 1.00, t2: 2.35 (1.04-5.32), t3: 2.11 (0.94-4.73) | Tertile | 0.086 | 1 |
|  |  |  | Vozarova et al. 2002 | 8.555 cell/mm3 | Coulter counter | HR, 2.7 (1.3-5.4) | 90th percentile | 0.007 | 1 |
|  | RDW | Clinical | Engström et al. 2014 | 40.1±3.3 (T2D) vs 40.9±3.4 fL (non-cases) | automatic analyser | HR, 0.68 (0.59-0.77) | Top quartile | <0.001 | 1,2 |
| Blood pressure | Na-Li countertransport (Na-Li CT) activity | Novel | Vaccaro et al./2005 | 364.6±184.8 μmol/l RBC/h | In-house assay (Canessa) | LR, β: 0.002, SE: 0.001 | Tertile | 0.004 | 2 |
| Blood volume | B-type natriuretic peptide | Novel | Salomaa, et al 2010 | 12.7 (7.2-27.0) (pg/mL) | CMIA | NR (only graphically shown) | SD | 0.0155 | 1,2,3 |
|  | Midregional-pro atrial natriuretic peptide (pmol/L) | Novel | Salomaa 2010 | 44.4 (31.9-59.0) (pmol/l) | ILA | NR (only graphically shown) | SD | 0.0019 | 1,2,3 |
|  | N-terminal-pro B-type natriuretic peptide (pg/mL) | Novel | Salomaa 2010 | 41.1 (20.8-82.6) | ECLIA | NR (only graphically shown) | SD | 0.0155 | 1,2,3 |
| Bone metabolism | Osteocalcin | Novel | Ngarmukos et al./2012 | 13.04 ± 0.48 μg/l | ECLIA | OR, 0.90 (0.81-0.99) | 1-unit | <0.05 | 1,2,3 |
| Calcitonin-related system inflammation | Procalcitonin | Novel | Abbasi,et al /2012 | 0.019 (0.015-0.023) (T2D) vs 0.016 (0.013-0.019) (non-case) ng/ml | ILA | OR, 1.32 (1.09-1.60) | log | 0.004 | 1,2 |
| Circadian rhythm | Melatonin | Novel | McMullan et al. 2013 | Median (5-95 pct range): 36.3 ng/mg (6.9-110.8) | ELISA | OR(T1-T3): 2.17 (1.18-3.98) | log2 | <0.001 | 1,2,3 |
| Electrolytes | Bicarbonate | Clinical | Mandel et al. 2012 | Median (IQR) controls: 22.4 (20.5–23.9) mmol/l | ET | OR(Q1-Q4): 0.75 (0.54–1.05) | Quartile 4 | 0.04 (trend) | 1,2,3 |
|  | Calcium | Clinical | Becerra-Tomàs et al, 2014 | t1:9.01, t3:10.2 mg/dl | automatic analyser | HR, 1.37 (0.88-2.14) | 1-unit | NR (not significant) | 1,2,3 |
|  | Magnesium | Clinical | Raynor et al./2013 | 1.63/1.65 mg/dl | Ginder and Heath technique | Change in C-index: from 0.8411 to 0.8409 | SD | <0.0001 | 1, 2, 3 |
| Endothelial dysfunction | C-terminal pro-endothelin-1 | Novel | Salomaa, et al 2010 | 51.2 (45.5-61.3) (pmol/L) | ILA | NR (only graphically shown) | SD | 0.4161 | 1,2,3 |
|  | Midregional-pro adrenomedullin (MR-proADM) | Novel | Salomaa 2010 | 0.5 (0.4-0.6) (nmol/l) | ILA | NR (only graphically shown) | SD | 0.7566 | 1,2,3 |
|  | Soluble E-selectin (sE-selectin) | Novel | Chao, et al 2010 | 49.3 (33.6-71.0) (T2D) vs 36.9 (26.0-50.9) (non-case) ng/ml | ELISA | C-index 0.93 | log | NA | 1,2,3 |
|  |  |  | Herder, et al 2011 | mean.geom (antilog se) 71.45 (2.00) (T2D) vs 54.01 (0.71) (non-case) ng/ml | ELISA | HR 1.67 (1.37-2.03) | SD | <0.001 | 1,2,3 |
|  |  |  | Krakoff J et al. 2003 | 76 (59–94) (ng/ml) | ELISA | 1.34 (0.91-1.99) | SD | 0.14 | 1,2,3 |
|  |  |  | Meigs et al. 2004 | Median (IQR): 45.4 ng/mL (5.8-60.1) | ELISA | RR(Qint1-Quint5): 4.61 (2.85-7.46) | Quintile 5 | <0.001 | 1,2,3 |
|  |  |  | Song et al./2007 | q1: 20, q2: 32, q3: 43, q4: 68 ng/ml | ELISA | RR, q1: 1.00, q2: 1.95 (1.54-2.47), q3: 2.39 (1.89-3.04), q4: 5.48 (4.33-6.94) | Quartile | <0.001 | 1 |
|  |  |  | Stranges et al./2008 | 51.41 (ng/ml) | ELISA | OR, t1: 1.00, t2: 1.88 (0.78-4.56), t3: 3.39 (1.47-7.83) | Tertile | 0.003 | 1 |
|  |  |  | Thorand et al./2006 | M: 78.3 ng/ml, F: 64.6 ng/ml | ELISA | HR, M: T1: 1.0, T2: 1.38 (0.96-1.99), T3: 3.01 (2.18-4.17), F: T1: 1.0, T2: 1.37 (0.90-2.10), T3: 3.29 (2.25-4.83) | Tertile | <0.001 | None |
|  | soluble intercellular adhesion molecule 1 (ICAM-1) | Novel | Chao, et al 2010 | 323.6 (268.2-383.9) (T2D) vs 280.4 (234.0-330.4) (non-case) ng/ml | ELISA | HR, 1.003 | log | <0.01 | 1,2,3 |
|  |  |  | Dallmeier, et al 2012 | 258 (223-292) (T2D) vs 236 (206-273) (non-case) ng/ml | ELISA | OR 1.18 (0.99–1.42) | 1-SD for log-transformed | 0.07 | 1,2,3 |
|  |  |  | Herder, et al 2011 | mean.geom (antilog se) 849.06 (14.91) (T2D) vs 754.24 (7.85) (non-case) ng/ml | ELISA | HR 1.29 (1.02-1.62) | SD | 0.031 | 1,2,3 |
|  |  |  | Hoogeveen, et al 2007 | 288.6 (T2D) vs 265.2 (non-case) ng/ml | ELISA | HR 1.5 (1.02-2.23) | Tertile 3 | NR | 1,2,3 |
|  |  |  | Krakoff J et al. 2003 | 338 (298–385) (ng/ml) | ELISA | NR (only graphically shown) | SD |  | 1,2,3 |
|  |  |  | Stranges et al./2008 | 268.32 ng/ml | ELISA | OR, t1: 1.00, t2: 0.93 (0.44-1.97), t3: 0.88 (0.42-1.87) | Tertile | 0.745 | 1 |
|  |  |  | Thorand et al./2006 | M: 879.5 ng/ml, F: 812.1 ng/ml | ELISA | HR, M: T1: 1.0, T2: 1.09 (0.78-1.54), T3: 2.16 (1.59-2.93), F: T1: 1.0, T2: 1.42 (0.96-2.12), T3: 2.24 (1.55-3.25) | Tertile | <0.001 | None |
|  | Vascular cell adhesion molecule 1 (VCAM-1) | Novel | Chao, et al 2010 | 765.2 (595.2-972.1) (T2D) vs 696.3 (543.0-861.8) (non-case) ng/ml | ELISA | C-index 0.93 | log | NA | 1,2,3 |
|  |  |  | Krakoff J et al. 2003 | 490 (444–562) (ng/ml) | ELISA | NR (only graphically shown) | SD |  | 1,2,3 |
|  |  |  | Meigs et al. 2004 | Median (IQR): 526 ng/mL (234.5-614.2) | ELISA | RR(Qint1-Quint5): 0.64 (0.41-1.00) | Quintile 5 | 0.05 | 1,2,3 |
|  |  |  | Song et al./2007 | q1: 464, q2: 619, q3: 779, q4: 1013 ng/ml | ELISA | RR, q1: 1.00, q2: 1.16 (0.94-1.43), q3: 1.29 (1.02-1.62), q4: 2.05 (1.62-2.59) | Quartile | <0.0001 | 1 |
| Extrcellular matrix system | Tissue inhibitor of metalloproteinase 1 (ng/mL) | Novel | Salomaa 2010 | 87.7 (75.6-98.8) (ng/mL) | CMIA | Nr (only graphically shown) | SD | 0.0801 | 1,2,3 |
| Fatty acids | a-linolenic acid (ALA) | Novel | Djousse´, et al 2011 | 0.14 (0.11-0.18) % | chromatography | OR 0.57 (0.36-0.90) | Quartile | 0.03 | 1,2,3 |
|  | Arachidonic acid | Novel | Mahendran et al. 2014 | 11.9±1.1 % | chromatography | OR, 1.24 (0.87-1.77) | log | 0.23 | 1,2 |
|  | dihomo-linoleic acid | Novel | Mahendran et al. 2014 | 1.5±0.3 % | chromatography | OR, 1.04 (0.92-1.18) | log | 0.63 | 1,2 |
|  | eicosapentaenoic acid (EPA)+docosahexaenoic acid (DHA) | Novel | Djousse´, et al 2011 | 3.42 (2.82-4.22) % | chromatography | OR .064 (0.41-1.01) | Quartile | 0.05 | 1,2,3 |
|  |  |  | Wu, et al 2011 | NR | NR | RR 0.94(0.75-1.17) | 100 g/d | 0.56 | 1,2,3 |
|  | DHA |  | Mahendran et al. 2014 | 6.2±1.1 % | chromatography | OR, 1.00 (0.71-1.41) | log | 0.99 | 1,2 |
|  | EPA |  | Mahendran et al. 2014 | 1.5±0.6 % | chromatography | OR, 1.04 (0.58-1.87) | log | 0.9 | 1,2 |
|  | Non-esterified fatty acid (NEFA) | Novel | Norberg et al./2007 | M: 0.29, F: 0.40 mM | RT | OR, M: 2.9 (1.7-4.9), F: 1.6 (0.9-2.8) | NR | M: 0.002, F: 0.31 | 2 |
|  |  |  | Steffen et al. 2015 | q1:0.13-0.39, q2:0.4-0.52, q3:0.53-0.67, q4:0.68-2.11 | Colorimetric method | HR, 1.56 (1.21-2.00) | Quartile 5 | 0.006 | 1,2 |
|  | Linoleic acid | Novel | Mahendran et al. 2014 | 8.3±1.1 % | chromatography | OR, 0.54 (0.35-0.82) | log | 0.004 | 1,2 |
|  | myristic acid | Novel | Ma, et al 2015 | q1:0.19(0.08-0.22),q5:0.37(0.34-1.6) % | chromatography | HR, 0.98 (0.65-1.14) | Quartile 5 | 0.7 | 1,2 |
|  | Oleic acid | Novel | Mahendran et al. 2014 | 11.9±0.8 % | chromatography | OR, 0.98 (0.59-1.62) | log | 0.92 | 1,2 |
|  | palmitic acid | Novel | Ma, et al 2015 | q1:23.4(19.5-24),q5:27.3(26.5-32.4) % | chromatography | HR, 1.89 (1.27-2.83) | Quartile 5 | 0.001 | 1,2 |
|  |  |  | Mahendran et al. 2014 | 22.5±0.9 % | chromatography | OR, 0.74 (0.48-1.12) | log | 0.15 | 1,2 |
|  | stearic acid | Novel | Ma, et al 2015 | q1:12.1(8.2-12.6), q5:14.9914.4-18.9) % | chromatography | HR, 1.62 (1.09-2.41) | Quartile 5 | 0.006 | 1,2 |
|  |  |  | Mahendran et al. 2014 | 15.5±0.5 % | chromatography | OR, 1.25 (0.63-2.5) | log | 0.52 | 1,2 |
|  | 7-hexadecenoic acid | Novel | Ma, et al 2015 | q1:0.07(0.04-0.07), q5:0.12(0.1-0.78) % | chromatography | HR, 0.83 (0.67-1.22) | Quartile 5 | 0.33 | 1,2 |
|  | vaccenic acid | Novel | Ma, et al 2015 | q1:1.1(0.79-1.1), q5:1.6(1.5-2.4) % | chromatography | HR, 0.56 (0.38-0.83) | Quartile 5 | 0.005 | 1,2 |
|  |  |  | Mahendran et al. 2014 | 1.1±0.1 % | chromatography | OR, 0.99 (0.71-1.41) | log | 0.97 | 1,2 |
|  | pentadecanoic acid | Novel | Santaren, et al 2014 | 0.25±0.06 % | chromatography | OR, 0.73 (0.56-0.95) | SD | 0.02 | 1,2 |
|  | palmitoleic acid | Novel | Mozaffarian, et al 2010 | 0.49±0.2 % of total fatty acids | chromatography | HR 1.12 (0.98-1.29) | SD | NR | 1,2 |
|  |  |  | Ma, et al 2015 | q1: 0.29(0.11-0.33), q5:0.73(0.61-1.9) % | chromatography | HR, 1.27 (0.84-1.92) | Quartile 5 | 0.24 | 1,2 |
|  |  |  | Mahendran et al. 2014 | 0.4±0.2 % | chromatography | OR, 1.35 (1.07-1.69) | log | 0.01 | 1,2 |
|  | trans-Palmitoleic acid | Novel | Mozaffarian, et al 2010 | 0.18±0.05 % of total fatty acids | chromatography | HR 0.38 (0.24-0.62) | Quintile 5 | <0.001 | 1,2 |
|  |  |  | Mozaffarian, et al 2013 | 0.06±0.03 % of total fatty acids | chromatography | HR 0.52 (0.32-0.85) | Quintile 5 | 0.02 | 1,2 |
| Fibrinolytic system | Activated partial thromboplastin time (aPTT) | Clinical | Raynor et al./2013 | 29.0/29.3 s | Automated coagulometer | Change in C-index: from 0.8411 to 0.8415 | SD | <0.0001 | 1, 2, 3 |
|  | Complement C3 (CC3) | Clinical | Raynor et al./2013 | 173.2/151.8 mg/dl | ITA | Change in C-index: from 0.8607 to 0.8623 | SD | <0.0001 | 1, 2, 3 |
|  | D-Dimer | Clinical | Salomaa, et al 2010 | 163.7 (100.0-250.0) (ng/ml) | MEIA | NR (only graphically shown) | SD | 0.5354 | 1,2,3 |
|  | Factor VIII | Clinical | Raynor et al./2013 | 135.1/126.1 (%) | One-stage assay | Change in C-index: from 0.8411 to 0.8418 | SD | <0.0001 | 1, 2, 3 |
|  | Plasminogen activator inhibitor-1 (PAI-1) | Novel | Alessi, et al/2011 | 14.3±10.6 (T2D) vs 8.12±6.9(non-case) IU/ml | TriniLIZE immunoreactivity assay | OR 1.40 (1.14-1.72) | 1-unit | 0.001 | 1,2,3 |
|  |  |  | Festa, et al 2002 | 24 (15–37.5) (T2D) vs 16 (9–27) (non-case) ng/ml | IA | OR 1.8 (1.45-2.25) | SD | 0.0001 | 1,2,3 |
|  |  |  | Hernestål-Boman, et al 2012 | 35.0 (23.9 - 46.1) (T2D) vs 21.2 (14.6 - 31.3) (non-case) ng/ml | ELISA | OR 1.61 (1.06-2.23) | SD | <0.05 | 1,2,3 |
|  |  |  | Stranges et al./2008 | 38.27 ng/ml | ELISA | OR, t1: 1.00, t2: 0.79 (0.37-1.69), t3: 0.74 (0.33-1.68) | Tertile | 0.47 | 1 |
|  | tissue plasminogen activator (tPA) |  | Hernestål-Boman, et al 2012 | 11.0 (8.7 - 13.7) (T2D) vs 7.7 (5.6 - 10.5) (non-case) ng/ml | ELISA | OR 1.22 (0.77-1.94 | SD | >0.05 | 1,2,3 |
|  |  |  | Wannamethee et al./2008 | 13.37 ng/ml | ELISA | RR, t1: 1.00, t2: 2.36 (1.40-3.99), t3: 4.54 (2.76-7.47) | Tertile | <0.0001 | 1,2 |
|  | tPA/PAI-1 complex | Novel | Hernestål-Boman, et al 2012 | 7.9 (5.2 - 0.4) (T2D) vs 3.8 (2.5 - 5.8) (non-case) ng/ml | ELISA | OR 1.70 (1.02-2.82) | SD | <0.05 | 1,2,3 |
|  | Vitronectin | Novel | Alessi, et al/2011 | 132±40 (T2D) vs 112±35(non-case) % | ELISA | OR 1.24 (1.01-1.53) | SD | 0.045 | 1,2,3 |
|  | von Willebrand Factor (VWF) | Clinical | Hernestål-Boman, et al 2012 | 142.9 (119.1 - 178.9) (T2D) vs 124.0 (96.0 - 159.6) % | ELISA | 1.33 (0.88-2.02) | SD | >0.05 | 1,2,3 |
|  |  |  | Krakoff J et al. 2003 | 106 (80–132) | ELISA | 0.73 (0.46-1.16) | SD | 0.19 | 1,2,3 |
|  |  |  | Thorand et al./2006 | M: 136.8%, F: 137.1% | ELISA | HR, M: T1: 1.0, T2: 1.67 (1.01-2.76), T3: 1.43 (0.85-2.40), F: T1: 1.0, T2: 0.91 (0.45-1.81), T3: 1.31 (0.69-2.49) | Tertile | 0.38 | None |
|  |  |  | Wannamethee et al./2008 | 146.4 IU/dl | ELISA | RR, t1: 1.00, t2: 1.04 (0.70-1.56), t3: 1.39 (0.94-2.03) | Tertile | 0.007 | 1,2 |
| Glycemia | Glucose | Clinical | Abbasi,et al /2012 | 4.9±1.2 mmol/l | ET | C-index 0.82 -0.91 | 1-unit | NA | 1,2,3 |
|  |  |  | Abbasi,et al /2012 | 4.9±1.2;4.7±0.6 mmol/l | ET | C-index 0.894; 0.849 | 1-unit | NA | 1,2,3 |
|  |  |  | Rathmann, et al 2010 | 5.9±0.56 (T2D) vs 5.43±0.49 (non-case) mmol/l | hexokinase method | OR, 1.07 | 1-unit mg/dl | 0.0001 | 1,2,3 |
|  |  |  | Rolandsson, et al 2001 |  | NR | OR(Q1-Q4): 7.2 (4.8-11.4); OR(Q1-Q4)males: 8.4 (4.2-16.5); OR(Q1-Q4)females: 6.6 (3.9-12.0) | Quartile | Nr | 1,2 |
|  |  |  | Wannamethee et al./2011 | ≥6.1 mmol/l | Glucose oxidase method | OR, 5.04 (3.73, 6.80) | ≥6.1 mmol ⁄ l | <0.0001 | 1,2,3 |
|  |  |  | Nguyen et al./2010 | NR | Glucose oxidase method | OR, 3.28 (1.29-8.33) | top decile | <0.05 | 1,2,3 |
|  |  |  | Neeland et al./2012 | 101 (92-114) mg/dl | Standard laboratory assay | OR, 1.88 (1.38-2.96) | SD | <0.001 | 1,2 |
|  |  |  | Tabak et al./2009 | 5.71±0.91 mmol/l | Glucose oxidase method | NR | NR | <0.0001 | 1 |
|  |  |  | Norberg et al./2006 | M: 6.0, F: 5.8 mmol/l | Reflotron analyser | OR, M: 18.8 (2.88-123.4), F: 10.5 (1.98-56.86) | 6.1–6.9 mmol ⁄ l | <0.001 | 1,2,3 |
|  |  |  | Norberg et al./2007 | M: 5.9, F: 5.9 mM | Reflotron analyser | OR, M: 8.1 (3.3-20.2), F:3.6 (1.8-7.1) | NR | <0.001 | 1,2,3 |
|  |  |  | Rolandsson et al./2001 | ≥5.6 mmol l-1 | NR | OR, q1:1.0, q2: 2.4 (1.6, 3.8), q3: 4.8 (3.2, 7.6), q4: 7.2 (4.8, 11.4) | Quartile | <0.05 | None |
|  |  |  | Schöttker et al./2011 | 100-125 mg/dl | NR | HR, 32.80 | >125 mg/dl | <0.001 | None |
|  |  |  | Song et al./2007 | 6.78 (5.89-8.17) mmol/ml | ET | RR, 3.65 (3.22-4.14) | 1-SD for log-transformed | <0.0001 | 1 |
|  |  |  | Soulimane et al./2011 | Inter99: 5.5±0.4, AusDiab: 5.4±0.5, DESIR: 5.3±0.5 mmol/l | Inter99: Hexokinase/G6PD, AusDiab: glucose oxidase (baseline)/hexokinase (folow-up), DESIR: glucose oxidase/peroxidase | NR | NR | Inter99: 0.03, AusDiab: <0.0001, DESIR: 0.10 | 1, 2 |
|  | Glucose (2hPG) |  | Daimon, et al 2003 | 99.3±21.7 mg/dl | ET | OR 1.443 | 10 mg/dl | NR | 1,2,3 |
|  |  |  | Norberg et al./2007 | M: 7.9, F: 8.4 mM | Reflotron analyser | OR, M: 1.7 (1.4-2.1), F: 1.4 (1.2-1.7) | NR | <0.001 | 2 |
|  |  |  | Rathmann, et al 2010 | 8.11±1.77 (T2D) vs 6.06±1.55 (non-case) mmol/l | hexokinase method | OR, 1.03 | 1-unit mg/dl | <0.0001 | 1,2,3 |
|  |  |  | Rolandsson et al./2001 | >7.5 mmol l-1 | NR | OR, q1: 1.0, q2: 2.6 (1.6, 4.2), q3: 5.2 (3.2, 8.4), q4:7.8 (4.8, 12.6) | Quartiel | <0.05 | None |
|  |  |  | Rolandsson, et al 2001 |  | NR | OR(Q1-Q4): 7.8 (4.8-12.6); OR(Q1-Q4)males: 9.9 (5.1-18.9); OR(Q1-Q4)females: 6.0 (4.0-12.0) | Quartile | Nr | 1,2 |
|  | HbA1c | Clinical | Abbasi,et al /2012 | 5.39±0.58% | ITL | C-index 0.86 -0.93 | 1-unit | NA | 1,2,3 |
|  |  |  | Abbasi,et al /2012 | 5.39±0.58% | ITL | C-index 0.92 | 1-unit | NA | 1,2,3 |
|  |  |  | Norberg et al./2006 | 4.70% | HPLC | OR, M: t1: 1.0, t2: 1.2 (0.28-5.34), t3: 16.0 (2.23-115.3), F: t1: 1.0, t2: 2.0 (0.45-8.89), t3: 19.6 (2.52-152.4) | Tertile | <0.001 | None |
|  |  |  | Pradhan, et al 2007 | 5.03±0.37% | TINIA | RR, 8.6 (6.5-11.6) | Quintile 5 | <0.001 | 1,2,3 |
|  |  |  | Rathmann, et al 2010 | 5.8±0.4 (T2D) vs 5.6±0.3 (non-case) % | TINIA | OR, 4.87 | 1-unit | <0.0001 | 1,2,3 |
|  |  |  | Schöttker et al./2011 | 5.7-6.4% | HPLC | HR, 4.57 (3.30-6.46) | 5.7–6.4% | <0.001 | 1,3 |
|  |  |  | Soulimane et al./2011 | Inter99: 5.4±0.4, AusDiab: 5.5±0.3, DESIR: 5.4±0.4 | Inter99: IE-HPLC, AusDiab: HPLC, DESIR: HPLC | NR | NR | Inter99: <0.0001, AusDiab: 0.003, DESIR: 0.0007 | 1, 2 |
|  |  |  | Selvin et al./2014 | 5.7±1.1 % | HPLC | HR, 8.53 (7.32-9.95) | top tertile | <0.0001 | 1.2.3 |
|  | Glycated albumin | Novel | Selvin et al./2014 | 13.4±3.4 % | enzymatic assay | HR, 2.97 (2.53-3.48) | top tertile | <0.0001 | 1.2.3 |
|  | HOMA-B | Clinical | Song et al./2007 | 75.1 (44.6-118) | Index | RR, 0.82 (0.77-0.87) | 1-SD for log-transformed | <0.0001 | 1 |
|  | HOMA-IR | Clinical | Nguyen et al./2010 | NR (levels in childhood) | Index | OR, 5.84 (2.51-13.60) | top decile | <0.0001 | 1,2,3 |
|  |  |  | Lee et al. 2014 | 1.1 (0.7-1.6) | Index | RR, 1.54 (1.00-2.39) | Top quartile | 0.1 | 1,2 |
|  |  |  | Song et al./2007 | 4.03 (2.48-6.28) | Index | RR, 3.57 (3.20-3.98) | 1-SD for log-transformed | <0.0001 | 1 |
|  | HOMA-S | Clinical | Tabak et al./2009 | 103.4±58.8 | RIA/ELISA | NR | NR | <0.0001 | 1 |
|  | Insulin | Clinical | Carnethon, et al 2002 | F-B:104.6±74.2/F-W:66.9±50 M-B:80.6±59.2/M-W:79.3±56.3 pmol/l | RIA | HR, F:1.31 (1.26-1.36)/M: 1.31 (1.24-1.38) | SD | NR | 1,2 |
|  |  |  | Gautier, et al 2010 | 35.7 (26.7-48.0)(BMI<27) vs 56.5 (42.4-79.5) (BMI≥27) pmol/l | NR | HR, BMI <27, 1.12 (0.87–1.43)/ BMI≥27, 1.39 (1.07–1.80) | log | 0.38/ 0.012 | 1,2,3 |
|  |  |  | Nguyen et al./2010 | NR (levels in childhood) | RIA | OR, 5.54 (2.33-13.19) | top decile | 0.0001 | 1,2,3 |
|  |  |  | Norberg et al./2007 | M: 15.0, F: 14.9 mU/l | MEIA | OR, M: 8.2 (4.3-15.6), F: 3.4 (2.0-5.7) | NR | <0.001 | 1 |
|  |  |  | Pradhan, et al 2003 | 77.5 (52.5-98.5) (T2D) vs 39.3 (28.8-56.7) pmol/l | double-antibody | OR, 5.6 (1.8–18) | Top quartile | <0.001 | 1,2 |
|  |  |  | Rathmann, et al 2010 | 13.4 (10.1-21.2) (T2D) vs 9.3 (6.8-13.5) (non-case) mU/l | MEIA | Change in C-index: from 0.844 to 0.845 | NR | NA | 1,2,3 |
|  |  |  | Salomaa 2010 | 5.3 (3.6-7.6) | CMIA | NR (only graphically shown) | SD | 0.0041 | 1,2,3 |
|  |  |  | Song et al./2007 | 12.6 (8.14-18.6) μIU/ml | ELISA | RR, 2.72 (2.48-2.99) | 1-SD for log-transformed | <0.0001 | 1 |
|  | Proinsulin | Novel | Norberg et al./2007 | M: 29.9, F: 23.5 pM | ELISA | OR, M: 16.8 (7.0-40.3), F: 5.7 (2.9-11.2) | NR | <0.001 | 2 |
|  |  |  | Pradhan, et al 2003 | ≤4, 13% (T2D) vs 72% (non-case)/ 4.01-6.99, 6% (T2D) vs 5% (non-case/ ≥7 pmol/l, 82% (T2D) vs 24% (non-case) | double-antibody | OR, 16 (5.8–47) | Top quartile | <0.001 | 1,2 |
|  |  |  | Schulze et al./2005 | 25.1 ± 21.8 pmol/l | RIA | OR, q1: 1.00, q2: 1.21 (0.49-2.97), q3: 2.93 (1.28-6.74), q4: 12.53 (5.65-27.79) | Quartile | <0.001 | 1,2,3, |
|  | Proinsulin/Insulin ratio |  | Schulze et al./2005 | 0.232 ± 0.175 | RIA | OR, q1: 1.00, q2: 1.04 (0.47-2.27), q3: 3.10 (1.54-6.25), q4: 6.09 (3.16-11.74) | Quartile | <0.001 | 1,2,3 |
| Gut hormone | Glucagon-like peptide-1 (GLP-1) | Novel | Zheng et al. 2014 | 2.71±0.84 (T2D) vs 3.27±0.99 (non-cases) pmol/l | enzymatic assay | RR, 0.59 (0.22-1.58) | top quartile | 0.01 | 1,2 |
| Hepatokine | Fetuin A | Novel | Ix 2008 | Median 0.87 g/L (IQR, 0.71-1.04 g/L | ELISA | HR(T1-3): 2.41 (1.28-4.53) | Tertile | 0.006 | 1,2,3 |
|  |  |  | Stefan et al. 2008 | q1: 158, q2: 201, q3: 227, q4: 255, q5: 304 μg/ml | ADIVA/ELISA | RR, 1.05 (1.02-1.07) | 10μg/ml | <0.001 | 1,2,3, |
|  |  |  | Stefan et al. 2014 | q1: 347, q5: 626 μg/ml | ITA | RR, 1.35 (1.16-1.58) | SD | NR | 1,2 |
|  | Fibroblast growth factor (FGF)-21 | Novel | Bobbet, et al 2013 | 226.4 (19.8-1296) (T2D) vs 315.6 (109.7-1081) (non-case) pg/ml | ELISA | OR, 2 (0.91-4.3) | Tertile | 0.086 | 1,2 |
| Immune system | Glutamate decarboxylase autoantibodies | Novel | Rolandsson, et al 2001 |  | RLBA | Not associated and not shown | NR | NR | 1,2,3 |
|  | Glutamic acid decarboxylase antibodies (GADA) | Novel | Vigo et al./2007 | 1->2.37 U/ml | RIA | HR, 1.04 (0.55-1.96) | > 1 U/ml | 0.03 | 2 |
|  | Insulinoma antigen 2 (IA-2) autoantibodies | Novel | Rolandsson, et al 2001 |  | RLBA | Not associated and not shown | NR | Nr | 1,2,3 |
|  | Neopterin (nmol/L) | Novel | Salomaa 2010 | 6.0 (4.8-6.9) (nmol/L) | ELISA | NR (only graphically shown) | SD | 0.0028 | 1,2,3 |
| Inflammation | C-reactive protein (CRP) | Clinical | Krakoff J et al. 2003 | 4.9 (2.1–8.6) mg/l | ELISA | IRR(sdizedvals): 0.96 (0.60-1.55) | SD | 0.88 | 1,2,3 |
|  |  |  | Lee et al. 2009 | Mean (SD) controls: 4.2 (2) mg/L | Automated clinical chemistry system | OR(T1-T3): 1.49 (1.03-2.15) | Tertile | 0.03 | 1,2 |
|  |  |  | Lyssenko et al. 2012 | Median (IQR), inter99 controls: 0.9 (0.4–2.4) mg/dL | ITA | 1.58 (1.31–1.88) | SD | NR | None |
|  |  |  | Marques-Vidal et al. 2012 | Unclearly reported | IA- latex HS | OR 1.53 (0.90–2.60) | top quartile | 0.12 | 1,2,3 |
|  |  |  | Norberg et al./2007 | M: 2.8, F: 3.7 mg/l | NR | OR, M: 1.9 (1.3-2.6), F: 2.2 (1.5-3.3) | NR | 0.005 | 2 |
|  |  |  | Stranges et al./2008 | 4.42 μg/ml | nephelometry | OR, t1: 1.00, t2: 2.00 (0.83-4.82), t3: 2.59 (1.09-6.18) | Tertile | 0.036 | 1 |
|  |  |  | Thorand et al./2003 | 2.1 mg/l | IRA | HR, q1: 1.0, q2: 1.80 (0.90-3.59), q3: 2.85 (1.49-5.43), q4: 2.84 (1.50-5.36) | Quartile | 0.003 | None |
|  |  |  | Thorand et al./2007 | M: 2.1, F: 3.3 mg/l | IRMA | HR, M: t1: 1.0, t2: 1.67 (1.17-2.40), t3: 2.10 (1.48-3.00), F: t1: 1.0, t2: 3.37 (1.93-5.91), t3: 7.90 (4.64-13.45) | Tertile | M: <0.001, F: <0.001 | 1 |
|  |  |  | Abbasi,et al /2012 | 1.2 (0.5-2.8) mg/l | nephelometry | OR,1.06 (0.97-1.16) | log2 | 0.16 | 1,2 |
|  |  |  | Brunner, et al/2008 | 2.58±5.21 mg/l | INA | OR, 1.17 (1.07-1.28) | log2 | 0.001 | 1,2,3 |
|  |  |  | Chao, et al 2010 | 4.0 (2.0-7.6) (T2D) vs 2.1 (0.8-4.4) mg/l | ITA | C-index 0.93 | log | NA | 1,2,3 |
|  |  |  | Dallmeier, et al 2012 | 3.51 (1.49-8.15) (T2D) vs 1.84 (0.90-4.39) (non-case) mg/l | nephelometry | OR 1.17 (0.94-1.46) | 1-SD for log-transformed | 0.16 | 1,2,3 |
|  |  |  | Doi Y, et al 2005 | F: 0.36 (0.06–3.22)/ M: 0.49 (0.07–7.14) mg/l | LEA | OR, F:2.25 (1.01–5.01) /M: 2.63 (1.23–5.65) | top tertile | F: <0.049/M: 0.014 | 1,2,3 |
|  |  |  | Eugen-Olsen, et al 2010 | 1.13 (0.23–6.79) to 3.20 (0.44–17.44) | ITA | HR 2.46 (1.1-5.51) | > 3mg/l | NR | 1,2,3 |
|  |  |  | Festa, et al 2002 | 2.40 (1.29–5.87) (T2D) vs 1.67 (0.75–3.41) (non-case) mg/l | IA | OR 1.12 (0.91–1.38) | SD | 0.3 | 1,2,3 |
|  |  |  | Herder, et al 2011 | mean.geom (antilog se) 2.53 (1.05) (T2D) vs 1.36 (1.03) (non-case) mg/l | hs-IRA | HR 0.98 (0.84-1.16) | SD | 0.846 | 1,2,3 |
|  |  |  | Hu, et al 2004 | 0.36 (T2D) vs 0.16 (non-case) mg/dl | hs-IRA | RR 4.36 (2.8-6.8) | Top quintile | <0.001 | 1,2 |
|  |  |  | Laaksonen et al. 2004 | 0.1-9.99 mg/L | IMA | OR, 1.85 (0.96-3.58) | > 3mg/l | 0.084 | 1,2,3 |
|  |  |  | Liu et al. 2007 | Median (IQR) in white controls: 2.03 0.90-4.12) pg/mL | ITA | RR(SD): 1.21 (1.11-1.31) | SD | <0.001 | 1,2,3 |
|  |  |  | Montonen, et al 2011 | 3.45±4.88 (T2D) vs 1.79±3.22 (non-case) mg/l | CL | RR 3.52 (2.12-5.84) | Top quintile | <0.001 | 1,2 |
|  |  |  | Onat, et al 2011 | F:2.2(1.033)/M:1.92(1.033) mg/l | nephelometry | RR, F:1.13(0.95-1.34)/M:1.43(1.16-1.76) | log | F:<0.05/M:NS | 1,2,3 |
|  |  |  | Pradhan, et al 2001 | 0.69 (0.42-1.0) (T2D) vs 0.26 (0.1-0.61) (non-case) mg/dl | LEINA | RR, 4.3 (1.1-7.1) | Top quartile | 0.01 | 1,2,3 |
|  |  |  | Salomaa, et al 2010 | 1.2 (0.6-2.4) | LIA | NR (only graphically shown) | SD | 0.0101 | 1,2,3 |
|  | Dipeptidyl peptidase IV (DPP-IV) | Novel | Luft et al. 2010 | Mean (controls): 388.9 ng/ml | ELISA | HR: 0.90 (0.58-1.40) | top quartile | 0.24 | 1,2,3 |
|  | Dipeptidyl peptidase IV (DPP-IV) activity | Novel | Zheng et al. 2014 | 7.27±1.27 (T2D) vs 5.8±1.03 (non-cases) nmol/min.ml | enzymatic assay | RR, 5.1 (1.48-17.61) | top quartile | 0.01 | 1,2 |
|  | Fibrinogen | Clinical | Dallmeier, et al 2012 | 382 (338-445) (T2D) vs 366 (324-413) (non-case) mg/dl | ELISA | OR 0.91 (0.75–1.11) | 1-SD for log-transformed | 0.35 | 1,2,3 |
|  |  |  | Festa, et al 2002 | 287.8±58.8 (T2D) vs 275.1±56.1 (non-case) mg/dl | clot-rate assay | OR 1.01 (0.83–1.22) | log | 0.9 | 1,2,3 |
|  |  |  | Stranges et al./2008 | 248.85 mg/dl | NR | OR, t1: 1.00, t2: 1.47 (0.68-3.17), t3: 1.40 (0.58-3.35) | Tertile | 0.435 | 1 |
|  | interferon-c-inducible protein-10 (IP-10) | Novel | Herder, et al 2011 | mean.geom (antilog se) 250.24 (1.04) (T2D) vs 213.23 (1.02) (non-case) pg/ml | Bead-based multiplex assay | HR 1.26 (0.96-1.65) | SD | 0.09 | 1,2,3 |
|  | Interleukin 1β (IL1β) | Novel | Marques-Vidal et al. 2012 | Unclearly reported | multiplexed particle-based flow cytometric cytokine assay | OR 0.74 (0.47–1.18) | Top quartile | 0.12 | 1,2,3 |
|  | Interleukin 2Ra (IL2Ra) | Novel | Lyssenko et al. 2012 | Median (IQR), inter99 controls: 90.7 (41.4–173) ug/dL | CLIMA | 1.11 (0.85–1.40) | SD | Nr | None |
|  | Interleukin-1 Receptor Antagonist | Novel | Salomaa 2010 | 242.6 (175.4-318.8) (pg/ml) | ELISA | NR (only graphically shown) | SD | 0.0022 | 1,2,3 |
|  | Interleukin-18 (IL-18) | Novel | Herder, et al 2011 | mean.geom (antilog se) 189.63 (1.03) (T2D) vs 158.23 (1.02) (non-case) pg/ml | Bead-based multiplex assay | HR 1.11 (1.01-1.22) | SD | 0.027 | 1,2,3 |
|  |  |  | Salomaa 2010 | 237.3 (176.6-307.9) (pg/mL) | ELISA | NR (only graphically shown) | SD | 0.0078 | 1,2,3 |
|  |  |  | Thorand et al./2005 | 188.7 pg/ml | Luminex | HR, q1: 1.0, q2: 1.13 (0.84-1.52), q3: 1.57 (1.18-2.09), q4: 1.96 (1.49-2.58) | Quartile | <0.001 | None |
|  | Interleukin-6 (IL-6) | Novel | Chao, et al 2010 | 2.6 (1.6-4.6) (T2D) vs 1.5 (1.0-2.8) (no-case) pg/ml | ELISA | C-index 0.93 | log | NA | 1,2,3 |
|  |  |  | Dallmeier, et al 2012 | 3.52 (2.27-5.08) (T2D) vs 2.38 (1.65-3.74) (non-case) pg/mLOR 1.07 (0.87–1.31) | ELISA | OR 1.07 (0.87–1.31) | 1-SD for log-transformed | 0.55 | 1,2,3 |
|  |  |  | Herder, et al 2011 | mean.geom (antilog se) 3.02 (1.04) (T2D) vs 1.97 (1.03) (non-case) pg/ml | ELISA | HR 1.08 (0.93-1.24) | SD | 0.309 | 1,2,3 |
|  |  |  | Hu, et al 2004 | 2.38 (T2D) vs 1.84 (non-case) pg/ml | ELISA | RR 1.91 (1.27, 2.86) | Top quintile | <0.001 | 1,2 |
|  |  |  | Krakoff J et al. 2003 | 4.9 (4.0–6.2) (pg/ml) | ELISA | 0.75 (0.45-1.30) | SD | 0.28 | 1,2,3 |
|  |  |  | Liu et al. 2007 | Median (IQR) in white controls: 1.44 (0.95-2.23) pg/mL | us-ELISA | RR(SD): 1.19 (1.08-1.31) | SD | <0.001 | 1,2,3 |
|  |  |  | Marques-Vidal et al. 2012 | Unclearly reported | multiplexed particle-based flow cytometric cytokine assay | OR 1.18 (0.73–1.93) | Top quartile | 0.72 | 1,2,3 |
|  |  |  | Norberg et al./2007 | M: 2.6, F: 3.1 pg/ml | ELISA | OR, M: 1.4 (0.97-2.1), F: 2.1 (1.3-3.4) | NR | M: 0.84, F: 0.04 | 2 |
|  |  |  | Pradhan, et al 2001 | 2 (1.43-2.78) (T2D) vs (1.38 (0.91-2.05) (non-case) pg/ml | ELISA | RR, 1.5 (0.5-4.8) | Top quartile | 0.23 | 1,2,3 |
|  |  |  | Rhee EJ et al./2012 | 4.9±6.7 pg/ml | ELISA | NR (not significant) | log | NR (not significant) | 1, 2 |
|  |  |  | Stranges et al./2008 | 2.88 pg/ml | ELISA | OR, t1: 1.00, t2: 1.20 (0.51-2.86), t3: 1.98 (0.92-4.29) | | 0.077 | 1 |
|  |  |  | Thorand et al./2007 | M: 2.9, F: 3.2 pg/ml | ELISA | HR, M: t1: 1.0, t2: 1.64 (1.15-2.34), t3: 2.58 (1.83-3.64), F: t1: 1.0, t2: 1.65 (1.03-2.66), t3: 4.08 (2.62-6.36) | Tertile | M: <0.001, F: <0.001 | 1 |
|  | Interleukin-8 (IL-8) | Novel | Herder, et al 2011 | mean.geom (antilog se) 7.95 (1.03) (T2D) vs 6.97 (1.02) (non-case) | Bead-based multiplex assay | HR 0.93 (0.75-1.16) | SD | 0.514 | 1,2,3 |
|  | Intracellular adhesion molecule-1 (ICAM-1) | Novel | Meigs et al. 2004 | Median (IQR): 247 ng/mL (21.6-277) | ELISA | RR(Q1-Q5): 1.75 (1.05-2.92) | Top quintile | 0.03 | 1,2,3 |
|  |  |  | Raynor et al./2013 | 277.3/255.8 ng/ml | ELISA | Change in C-index: from 0.8607 to 0.8644 | SD | <0.0001 | 1, 2, 3 |
|  |  |  | Sattar et al./2009 | 388.6±1.42 ng/ml | ELISA | HR, 1.82 (1.27-2.63) | log | 0.0013 | 1, 2, 3 |
|  |  |  | Song et al./2007 | q1: 196, q2: 259, q3: 302, q4: 382 ng/ml | ELISA | RR, q1: 1.00, q2: 1.11 (0.88-1.40), q3: 1.58 (1.26-1.98), q4: 3.32 (2.67-4.11) | Quartile | <0.0001 | 1 |
|  | macrophage-migration inhibitory factor (MIF) | Novel | Herder, et al 2011 | mean.geom (antilog se) 18.27 (1.02) (T2D) vs 17.93 (1.01) (non-case) ng/ml | ELISA | HR 0.95 (0.75-1.21) | SD | 0.697 | 1,2,3 |
|  | Monocyte chemoattractant protein-1 (MCP-1) | Novel | Rhee EJ et al./2012 | 341.6±257.0 pg/ml | ELISA | NR (not significant) | log | NR (not significant) | 1, 2 |
|  |  |  | Dallmeier, et al 2012 | 146 (122-171) (T2D) vs 138 (116-163) (non-case) pg/mlOR 1.17 (0.96–1.43) | ELISA | OR 1.17 (0.96–1.43) | 1-SD for log-transformed | 0.13 | 1,2,3 |
|  |  |  | Herder, et al 2011 | 207.29 (0.819) (T2D) vs 177.88 (0.74) (non-case) pg/ml | Bead-based multiplex assay | HR 1.16 (0.92-1.45) | SD | 0.213 | 1,2,3 |
|  | Myeloperoxidase (µg/L) | Novel | Salomaa 2010 | 14.8 (10.4-19.5) | CMIA | NR (only graphically shown) | SD | 0.3107 | 1,2,3 |
|  | Osteoprotegerin | Novel | Dallmeier, et al 2012 | 37 (30-48) (T2D) vs 35 (28-44) (non-case) pmol/l | ELISA | OR 0.98 (0.8-1.2) | 1-SD for log-transformed | 0.86 | 1,2,3 |
|  | oxidised LDL (ox-LDL) | Novel | Hoogeveen, et al 2007 | 42.6 (T2D) vs 39.3 (non-case) U/l | ELISA | HR 1.12 (0.77-1.62) | Top tertile | NR | 1,2,3 |
|  | regulated on activation, normal T-cell expressed and secreted (RANTES) | Novel | Herder, et al 2011 | mean.geom (antilog se) 24700 (1.03) (T2D) vs 23169 (1.02) (non-case) pg/ml | ELISA | HR 1.05 (0.82-1.34) | SD | 0.702 | 1,2,3 |
|  | soluble CD163 | Novel | Møller, et al 2011 | F: 1.71(1.31-2.26)/ M: 1.76(1.37-2.36) mg/l | ELISA | OR 2.6 (2.1-3.2) | log | <0.001 | 1,2 |
|  | soluble CD36 | Novel | Handberg, et al 2010 | F: 2±0.8 (T2D) vs 1.5±0.8 (referent)/ M: 2.3±0.6 (T2D) vs 2±0.9 (referent) | ELISA | OR, 1.13 (0.77-1.640 | log2 | 0.54 | 1,2,3 |
|  | soluble urokinase plasminogen activator receptor(suPAR) | Novel | Eugen-Olsen, et al 2010 | 4.03 (1.3-19.9) ng/ml | ELISA | HR 1.23 (1.09-1.39) | 1-ng | NR | 1,2,3 |
|  |  |  | Haugaard, et al 2012 | 4.47±1.33 (T2D) vs 4.21±1.35 (non-case) ng/ml | ELISA | HR, non-smoker middle-aged, 4.49 (1.44–14.0)/ non-smoker older 2.89 (1.33–6.29) | Top quartile | 0.004/0.0008 | 1,2,3 |
|  |  |  | Krakoff J et al. 2003 | 3.3 (2.2–4.5) (ng/ml) | ELISA | NR (only graphically shown) | SD |  | 1,2,3 |
|  | transforming growth factor-b1 (TGF-b1) | Novel | Herder, et al 2011 | mean.geom (antilog se) 34.87 (1.01) (T2D) vs 34.22 (1.01) (non-case) ng/ml | ELISA | HR 1.20 (0.94-1.53) | SD | 0.145 | 1,2,3 |
|  | Tumor necrosis factor α (TNF-α) | Novel | Daimon, et al 2003 | 11.4±1.35 pg/ml | ELISA | OR 1.17 (0.815-1.67) | 0.1 log μg/ml | 0.4 | No |
|  |  |  | Krakoff J et al. 2003 | 3.0 (2.4–4.1) (pg/ml) | ELISA | NR (only graphically shown) | SD |  | 1,2,3 |
|  |  |  | Marques-Vidal et al. 2012 | Unclearly reported | multiplexed particle-based flow cytometric cytokine assay | OR 0.65 (0.41–1.04) | top quartile | 0.054 | 1,2,3 |
|  |  |  | Norberg et al./2007 | M: 1.2, F: 1.6 pg/ml | ELISA | OR, M: 1.03 (0.7-1.4), F: 1.1 (0.7-1.6) | NR | M: 0.12, F: 0.50 | 2 |
|  | Tumor necrosis factor α receptor 2 (TNF-α–R2) | Novel | Chao, et al 2010 | 2632.7(2190.6-3296.6) (T2D) vs 2361.4 (1927.5-2883.6) (non-case) pg/ml | ELISA | C-index 0.93 | log | NA | 1,2,3 |
|  |  |  | Dallmeier, et al 2012 | 2100 (1804-2570) (T2D) vs 1888 (1586-2256) (non-case) pg/mL | ELISA | OR 1.07 (0.88–1.30) | 1-SD for log-transformed | 0.49 | 1,2,3 |
|  |  |  | Hu, et al 2004 | 2646.5 (T2D) vs 2383.8 (non-case) pg/ml | ELISA | RR 1.64 (1.10-2.45) | Top quintile | <0.001 | 1,2 |
|  |  |  | Rhee EJ et al./2012 | 3.4±1.7 pg/ml | ELISA | OR, 1.45 (0.80-2.62) | log | 0.218 | 1, 2 |
|  |  |  | Liu et al. 2007 | Median (IQR) in white controls: 2517 (2048-3045) pg/mL | ELISA | RR(SD): 1.16 (1.05-1.27) | SD | 0.003 | 1,2,3 |
|  | Visfatin | Novel | Rhee EJ et al./2012 | 24.1±56.0 ng/ml | ELISA | NR (NS) | NR | NR (NS) | 1, 2 |
| Iron metabolism | Ferritin | Clinical | Lyssenko et al. 2012 | Median (IQR), inter99 controls: 365.4 (285.7–446.9) U/mL | CLIMA | 1.07 (0.81–1.30) | SD | NR | None |
|  |  |  | Montonen, et al 2012 | F: 6.39 (2-11)/ M: 19 (8-28) mg/l | CL | RR 1.73 (1.15-2.61) | Top quintile | 0.002 | 1,2,3 |
|  |  |  | Raynor et al./2013 | 202.9/144.4 ng/ml | ITA | Change in C-index: from 0.8607 to 0.8644 | SD | <0.0001 | 1, 2, 3 |
|  |  |  | Salomaa, et al 2010 | 56.1 (27.6-124.5) (ng/ml) | CMIA | NR (only graphically shown) | SD | 0.0013 | 1,2,3 |
|  |  |  | Sun et al./2013 | M: q1: 62.5, q2: 109, q3: 149, q4: 197, 325; F: q1: 46, q2; 79.5, q3: 149, q4: 142, q5: 235 | PEITA | RR, 1.90 | Quartile | 0.002 | 1,2,3 |
|  | soluble transferrin receptor (sTfR) | Novel | Montonen, et al 2012 | F: 1 (0.8-1.2)/ M: 1.1 (0.9-1.3) mg/l | Nephelometry | RR 1.21 (0.78-1.89) | Top quintile | 0.8 | 1,2,3 |
|  | Transferrin | Clinical | Mainous et al. 2002 | 8% participants had levels >45% | Nr | OR, 0.89 (0.59-1.34) | >45% | Nr | 1,2,3 |
| Lipid | Apolipoprotein A1 | Clinical | Salomaa, et al 2010 | 1.5 (1.3-1.7) (g/l) | ITA | NR (only graphically shown) | SD | 0.0349 | 1,2,3 |
|  | Apolipoprotein A-II (Apo A-II) | Clinical | Onat et al./2009 | M: 0.304, F: 0.337 g/l | Nephelometry | RR, 4.5 (1.3-15.6) | >30, > 33 g/l | NR | 1 |
|  | Apolipoprotein B | Clinical | Ley et al. 2010 | Mean (SD): 0.99 (0.27) g/L | SM | OR 1.50 (1.11-2.02) | SD | NR | 1,2,3 |
|  |  |  | Onat, et al 2007 | F: 114.7±42/ M: 114.7±40.8 | nephelometry | RR, F: 1.68 (0.87-3.26)/ M:0.86 | >120 mg/dl | NR | 1,2 |
|  |  |  | Salomaa, et al 2010 | 1.0 (0.8-1.2) (g/l) | ITA | NR (only graphically shown) | SD | <0.001 | 1,2,3 |
|  | High density lipoprotein cholesterol (HDL cholesterol) | Clinical | Gast GS, et al 2012 | 1.26±0.31 mmol/l | ET | HR 1.13 (1.02-1.26) | 0.1-mmol/L descrease | NR | 1,2,3 |
|  |  |  | Gautier, et al 2010 | 1.65 (1.39-1.96)(BMI<27) vs 1.44 (1.19, 1.70) (BMI≥27) mmol/l | automatic analyser | HR, BMI <27, 0.89 (0.68–1.15)/ BMI≥27, 0.77 (0.58–1.02) | SD | 0.37/ 0.064 | 1,2,3 |
|  |  |  | Ley et al. 2010 | 1.26 (0.28) mmol/l | SM | OR 0.84 (064-1.11) | SD | NR | 1,2,3 |
|  |  |  | Montonen, et al 2011 | 1.13±0.28 (T2D) vs 1.37±0.36 (non-case) mmol/l | CL | RR 0.26 (0.16-0.4) | Top quintile | <0.001 | 1,2 |
|  |  |  | Norberg et al./2007 | M: 1.0, F: 1.2 mM | RT | OR, M: 0.1 (0.04-0.3) F: 0.1 (0.04-0.3) | NR | <0.001 | 2 |
|  |  |  | Wannamethee et al./2011 | M: <1.03, F: <1.28 mmol/l | Hitachi 747 analyser | OR, 1.79 (1.37, 2.35) | < 1.28 mmol ⁄ l | <0.0001 | 1,2,3 |
|  | TG/HDL ratio | Clinical | Lee et al. 2014 | NR | Colorimetric method | RR, 2.21 (1.57-3.11) | Top quartile | 0.0002 | 1,2 |
|  | TyG index | Clinical | Lee et al. 2014 | 8.7±0.6 | Colorimetric method | RR, 4.09 (2.74-6.21) | Top quartile | <0.0001 | 1,2 |
|  | Low-density lipoprotein (LDL) | Clinical | Ley et al. 2010 | 2.42 (0.74) mmol/l | SM | OR 1.25 (0.94-1.66) | SD | Nr | 1,2,3 |
|  | Triglycerides (TG)s | Clinical | Gautier, et al 2010 | 0.88 (0.63, 1.21)(BMI<27) vs 1.25 (0.90, 1.82)(BMI≥27) mmol/l | automatic analyser | HR, BMI <27, 1.34 (1.06–1.69)/ BMI≥27, 1.41 (1.10–1.81) | | 0.014/0.0067 | 1,2,3 |
|  |  |  | Hjellvik, et al 2012 | F: 2.4 (T2D) vs 1.3 (non-case)/ M: 3.2 (T2D) vs 2.1 (non-case) mmol/l | NR | RR, F: BMI≤24.1, 4.4 (1.9-10.2), BMI≥30.2, 1.2 (0.7-2.1)/ M: BMI≤25.9, 2.8 (1.5–5.2), BMI ≥30.5, 2.2 (1.4–3.6) | Quantile | NR | 1,2,3 |
|  |  |  | Ley et al. 2010 | Median (IQR): 1.10 (0.81–1.53) | SM | OR 1.49 (1.12-1.98) | SD | Nr | 1,2,3 |
|  |  |  | Norberg et al./2006 | M: 2.3, F: 1.7 mmol/l | HPLC | OR, M: 2.1 (0.64-6.61), F: 1.0 (0.33-3.26) | NR | <0.001 | None |
|  |  |  | Norberg et al./2007 | M: 2.3, F: 1.8 mM | RT | OR, M: 2.6 (1.8-3.7), F: 2.2 (1.4-3.3) | NR | <0.001 | 2 |
|  |  |  | Wannamethee et al./2011 | ≥1.7 mmol/l | Hitachi 747 analyser | OR, 1.42 (1.08, 1.87) | ≥ 1.7 mmol ⁄ l | <0.0001 | 1,2,3 |
| Liver function | Alanine aminotransferase (ALT) | Clinical | Abbasi,et al /2012 | 20.2±11.9; 23.9±20.1 U/l | ET | HR 1.05 (0.93-1.18); OR 1.29 (1.11-1.50) | log2 | NR | 1,2,3 |
|  |  |  | André, et al 2005 | F:20.1±12.3/M:30.8±18.0 U/l | ET | OR, F: 0.7 (0.2-2.1)/M: 1.5 (0.6-3.8) | Top quartile | F: 0.64/M: <0.17 | 1,2 |
|  |  |  | Choi, et al 2013 | 17.1±5.4 (control) to 45.9±20.8 (subjects with↑ALT and steatosis) | ET | HR 1.20 (0.82-1.54) | >30 IU/L | NA | 1,2,3 |
|  |  |  | Doi Y, et al 2007 | F: 11 (6-24)/ M: 14 (7-38) U/l | ET | OR, F: 4.4 (1.38-14.06)/ M:2.32 (0.91-9.07) | Top quartile | F: 0.0077/ M:0.016 | |
|  |  |  | Ford ES, et al 2008 | 20.5±13.8 (U/l) | spectrophotometric | HR 1.93 (1.27–2.92) | Top quintile | <0.001 | 1,2,3 |
|  |  |  | Gautier, et al 2010 | 19.9 (15.0-26.9)(BMI<27) vs 26.8 (19.9-37.7) (BMI≥27) | automatic analyser | HR, BMI <27, 1.34 (1.08–1.68)/ BMI≥27, 1.37 (1.06–1.77) | SD | <0.009/ 0.018 | 1,2,3 |
|  |  |  | Goessling W, et al 2008 | 24±14 U/l | Kinetic method | OR 1.48 (1.30–1.69) | SD | <0.001 | 1,2,3 |
|  |  |  | Kim et al. 2008 | Median 19.5 (IQR 14.5-25.5) | Toshiba autoanalyser | OR, 4.31 (1.56-11.88) | Top quintile | 0.014 | 1,2,3 |
|  |  |  | Lee et al. 2003 | NR | automatic analyser | RR, 5.6 (2.7-11.5) | > 50 U/L | <0.001 | 1,2,3 |
|  |  |  | Montonen, et al 2011 | 34.3±23.2 (T2D) vs 22±14.9 U/l | CL | RR, 3.01 (1.94-4.67) | Top quintile | <0.001 | 1,2 |
|  |  |  | Nguyen et al./2011 | 27.1 ± 21.1 UI/l | ET | OR, 1.16 (1.00-1.35) | SD | 0.05 | 2,3 |
|  |  |  | Shlomai et al./2010 | M: >22 U/l, F: >10 U/l | Olympus kit and reagents | HR, 5.630 (4.749-6.674) | log | 0 | 1,3 |
|  |  |  | Vozarova et al./2002 | 45 ± 29 U/l | Colorimetric method | RH, 1.9 (1.1-3.3) | 90th percentile | 0.02 | 1,2,3, |
|  | Albumin | Clinical | Abbasi,et al /2012 | 38.9±4.9; 45.8±2.7 g/l | Colorimetric method | HR 0.42 (0.24-0.74); OR 0.31 (0.87-1.05) | log2 | NR | 1,2,3 |
|  |  |  | Raynor et al./2013 | 3.9/3.8 g/dl | Colorimetric method | Change in C-index: from 0.8411 to 0.8409 | SD | 0.22 | 1,2,3 |
|  |  |  | Stranges et al./2008 | 4.27 g/dl | NIA | OR, t1: 1.00 ( t2: 0.62 (0.31-1.24), t3: 0.36 (0.15-0.85) | Tertile | 0.019 | 1 |
|  | Asparate aminotransferase (AST) | Clinical | Abbasi,et al /2012 | 22.7±9.1; 25.7±10.4 U/l | ET | HR 1.04 (0.87-1.22); OR 1.16 (0.89-1.50) | log2 | NR | 1,2,3 |
|  |  |  | André, et al 2005 | F:18.0±8.2/M:22.9±10.5U/l | ET | OR, F: 0.7 (0.3-1.9)/M: 1.5(0.6-3.6) | Top quintile | F: <0.64/M: 0.32 | 1,2 |
|  |  |  | Doi Y, et al 2007 | F: 19 (12-33)/ M:22 (14-45) | ET | OR, F: 1.26 (0.55-2.92)/M: 1.87 (0.77-4.53) | Top quintile | F:0.17/M:0.17 | 1,2,3 |
|  |  |  | Goessling W, et al 2008 | 20±9 U/l | Kinetic method | OR 1.33 (1.17–1.53) | SD | <0.001 | 1,2,3 |
|  |  |  | Lee et al. 2003 | Median (IQR): 23 (18-28) U/L | SMA- CII continuous-flow analyzer | RR 2.9 (1.2-6.9) | 90th percentile | 0.002 | 1,2,3 |
|  | Bilirubin | Clinical | André, et al 2005 | F:10.2±5.4/M:12.4±5.8U/l | ET | OR, F: 0.6 (0.2-1.8)/M: 1.1(0.5-2.4) | Top quintile | F: 0.60/M: <0.93 | 1,2 |
|  | γ-glutamyltranspeptidase (GGT) | Clinical | Abbasi,et al /2012 | 25.5±20; 36.6±28.9 U/l | ET | HR 1.45 (1.34-1.58); OR 1.22 (1.09-1.38) | log2 | <0.01 | 1,2,3 |
|  |  |  | Abbasi,et al /2012 | 25.5±20U/l | ET | C-index 0.88 | log | NA | 1,2,3 |
|  |  |  | André, etal/ 2005 | F:22.2±21.3/M:41.2±40.1U/l | ET | OR, F: 2.6 (0.6-12.5)/M: 3.9 (1.1-13.6) | Top quintile | F: <0.06/M: <0.01 | 1,2,3 |
|  |  |  | Doi Y, et al 2007 | F: 13 (8-35)/ M:22 (11-95) U/l | ET | OR, F: 5.73 (1.62-20.19)/M: 2.54 (1.03-6.26) | Top quintile | F:0.0017/M: 0.008 | 1,2,3 |
|  |  |  | Ford ES, et al 2008 | 25.8±62.5 (U/l) | spectrophotometric | HR 2.67 (1.63–4.37) | Top quintile | <0.001 | 1,2,3 |
|  |  |  | Gautier, et al 2010 | 19.7 (14.7-29.7) (BMI<27) vs 29.4 (19.7-46.2) (BMI≥27) | automatic analyser | HR, BMI <27, 1.59 (1.29–1.97)/BMI≥27,1.07 (0.82–1.38) | SD | <0.0001/ 0.63 | 1,2,3 |
|  |  |  | Kim et al. 2008 | Median 15.5 (IQR 10.5-24.5) | automatic analyser | OR 3.07 (1.21-7.76) | Top quartile | 0.001 | 1,2,3 |
|  |  |  | Lee et al. 2003 | NR | automatic analyser | RR 25.8 (6.9-96.4) | > 50 U/L | <0.001 | 1,2,3 |
|  |  |  | Lee et al. 2003 | Median (IQR): 12 (17-25) U/L | measured colori- metrically by the nitroanilide method | RR 2.0 (1.1-3.5) | 90th percentile | <0.05 | 1,2,3 |
|  |  |  | Lindsay et al. 2001 | Mean(1SDrange): 1.16 (1.159–1.168) g/dL | zinc sulfate turbidity method | HR 1.14 (1.05-1.24) | SD | <0.05 | 1,2,3 |
|  |  |  | Marques-Vidal et al. 2012 | Unclearly reported | SM | OR 1.45 (0.85–2.46) | Top quartile | 0.17 | 1,2,3 |
|  |  |  | Montonen, et al 2011 | 48.1±63.3 (T2D) vs 27.6±71.7 U/l | CL | RR 4.00 (2.42-6.62) | Top quintile | <0.001 | 1,2 |
|  |  |  | Nguyen et al./2011 | 23.9 ± 18.7 UI/l | ET | OR, 1.20 (1.06-1.35) | SD | <0.01 | 2,3 |
|  |  |  | Raynor et al./2013 | 21.0/05.9 U/l | Automated chemistry assay | Change in C-index: from 0.8607 to 0.8613 | SD | 0.002 | 1,2,3 |
|  |  |  | Salomaa 2010 | 26.5 (17.0-37.0) | RT | NR (only graphically shown) | SD | <0.001 | 1,2,3 |
|  |  |  | Xu et al./2011 | q1: 14, q2: 18, q3: 23, q4: 37 IU/l | Autoanalyzer | HR, q1-2: 1.0, q3: 2.50 (0.55-11.37), q4: 2.74 (1.45-5.18) | Quartile | 0.001 | 1 |
| Metabolomics | 2-Aminoadipic acid (2-AAA) | Novel | Wang et al./2013 | 1.55 μM | HILIC/LC-MS | OR, 1.59 (1.28-1.97) | SD | <0.0001 | 1,2,3 |
|  | a-hydroxybutyrate (a-HB) | Novel | Ferrannini, et al 2013 | 3.83 (1.62) (T2D) vs 3.49 (1.55) (non-case) μg/mL | MS | OR, 1.26 (1.07–1.48) | SD | NR | 2 |
|  | diacyl phosphatidylcholine C32:1 | Novel | Floegel, et al 2013 | 16.9±9.76 (subcohort) vs 20.4±11.3 (T2D) μmol/l | FIA-MS | HR 1.15 (1.03–1.29) | SD | NR | 1,2,3 |
|  | diacyl phosphatidylcholine C36:1 |  | Floegel, et al 2013 | 57.3±15.6 (subcohort) vs 62.1±17.2 (T2D) μmol/l | FIA-MS | HR 1.25 (1.10–1.41) | SD | NR | 1,2,3 |
|  | diacyl phosphatidylcholine C38:3 |  | Floegel, et al 2013 | 55.4±14.8 (subcohort) vs 65.3±18.2 (T2D) μmol/l | FIA-MS | HR 1.38 (1.22–1.55) | SD | NR | 1,2,3 |
|  | diacyl phosphatidylcholine C40:5 |  | Floegel, et al 2013 | 11.4±3.49 (subcohort) vs 12.8±4.17 (T2D) μmol/l | FIA-MS | HR 1.19 (1.06–1.32) | SD | NR | 1,2,3 |
|  | Fructosamine | Novel | Neeland et al./2012 | 211 (196-224) μmol/l | NR | OR, 1.42 (1.14-1.75) | SD | <0.001 | 1,2 |
|  |  | Novel | Selvin et al./2014 | 237 (89-706) μmol/l | Colorimetric method | HR, 2.26 (1.91-2.67) | Top tertile | <0.0001 | 1,2,3 |
|  | Glycine | Novel | Wang-Sattler et al./2012 | 263.02 ± 74.35 μM | LC/FIA-MS | OR, 0.73 (0.55-0.97) | SD | 0.04 | 1,2 |
|  | Hexose | Novel | Floegel, et al 2013 | 4698±984 (subcohort) vs 5783±2167) (T2D) μmol/l | FIA-MS | HR 1.51 (1.28–1.80) | SD | NR | 1,2,3 |
|  | Homocysteine (µmol/L) | Novel | Salomaa 2010 | 11.5 (9.5-13.7) | FPIA | NR (only graphically shown) | SD | 0.0684 | 1,2,3 |
|  | Isoleucine | Novel | Wang et al./2011 | 85.1 (72.3, 102.5) μM | LC-MS | OR, 1.70 (1.27-2.28) | SD | 0.0004 | 1,2,3 |
|  | Lactate | Novel | Juraschek et al. 2013 | mean: 7.67 mg/dL (IQR: 5.80-9.70) | automatic analyser | HR 2.05 (1.28-3.28) | Top quartile | 0.001 | 1,2,3 |
|  | Leucine | Novel | Wang et al./2011 | 145.1 (123.4, 165.8) μM | LC-MS | OR, 1.62 (1.20-2.17) | SD | 0.001 | 1,2,3 |
|  | Linoleoylglycerophosphocholine (L-GPC) | Novel | Ferrannini, et al 2013 | 14.0 (3.56) (T2D) vs 15.92 (5.48) (non-case) μg/mL | MS | OR 0.67 (0.54–0.84) | SD | NR | 2 |
|  | Lyso phosphatidylcholine | Novel | Floegel, et al 2013 | 35.2±13.7 (subcohort) vs 29.6±11.1 (T2D) μmol/l | FIA-MS | HR 0.84 (0.73–0.96) | SD | NR | 1,2,3 |
|  | Lysophosphatidylcholine (LPC) (18:2) | Novel | Wang-Sattler et al./2012 | 28.09 ± 8.99 μM | LC/FIA-MS | OR, 0.70 (0.51-0.94) | SD | 0.02 | 1,2 |
|  | Lysophosphatidylethanolamines (LPE) 18:2 | Novel | Rhee EP et al./2011 | NA | LC/MS | OR, 1.39 (1.07-1.81) | log | 0.016 | 1, 2, 3 |
|  | PC 34:2 | Novel | Rhee EP et al./2011 | NA | LC/MS | OR, 1.47 (1.06-2.04) | log | 0.021 | 1, 2, 3 |
|  | PC 36:2 | Novel | Rhee EP et al./2011 | NA | LC/MS | OR, 1.35 (1.02-1.80) | log | 0.039 | 1, 2, 3 |
|  | Phenylalanine | Novel | Floegel, et al 2013 | 56.4±12 (subcohort) vs 61±12.9 (T2D) μmol/l | FIA-MS | HR 1.25 (1.12–1.39) | SD | NR | 1,2,3 |
|  |  |  | Wang et al./2011 | 62.0 (57.0, 67.9) μM | LC-MS | OR, 2.02 (1.40-2.92) | SD | 0.0002 | 1,2,3 |
|  | TAG 44:1 | Novel | Rhee EP et al./2011 | NA | LC/MS | OR, 1.41 (1.02-1.94) | log | 0.036 | 1, 2, 3 |
|  | TAG 46:1 |  | Rhee EP et al./2011 | NA | LC/MS | OR, 1.44 (1.01-2.06) | log | 0.043 | 1, 2, 3 |
|  | TAG 48:0 |  | Rhee EP et al./2011 | NA | LC/MS | OR, 1.41 (1.01-1.95) | log | 0.042 | 1, 2, 3 |
|  | TAG 48:1 |  | Rhee EP et al./2011 | NA | LC/MS | OR, 1.47 (1.05-2.05) | log | 0.026 | 1, 2, 3 |
|  | TAG 50:0 |  | Rhee EP et al./2011 | NA | LC/MS | OR, 1.74 (1.19-2.57) | log | 0.005 | 1, 2, 3 |
|  | TAG 52:1 |  | Rhee EP et al./2011 | NA | LC/MS | OR, 1.94 (1.18-3.20) | log | 0.009 | 1, 2, 3 |
|  | Tyrosine | Novel | Wang et al./2011 | NR | LC-MS | OR, 1.85 (1.35-2.55) | SD | 0.0001 | 1,2,3 |
|  | Valine | Novel | Wang et al./2011 | 191.4 (172.2, 213.0) μM | LC-MS | OR, 1.57 (1.17-2.09) | SD | 0.002 | 1,2,3 |
| Muscle function | Troponin I | Clinical | Salomaa 2010 | 0.0 (0.0-0.0) (ng/ml) | CMIA | NR (only graphically shown) | SD | 0.6805 | 1,2,3 |
| Muscle metabolism | Creatine-Kinase MB | Clinical | Salomaa, et al 2010 | 1.1 (0.7-1.6) (ng/ml) | CMIA | NR (only graphically shown) | SD | 0.0072 | 1,2,3 |
| Nutrition status | δ13Carbon isotope | Novel | Patel, et al 2014 | ‒22.8±0.4 ‰ | MS | HR, 0.75 (0.65-0.83) | Top tertile | <0.001 | 1,2 |
|  | δ15Nitrogen isotope | Novel | Patel, et al 2014 | 10.2±0.4 ‰ | MS | HR, 1.22 (1.09-1.38) | Top tertile | 0.001 | 1,2 |
| Oxidation product (xanthines) | Uric acid | Clinical | Abbasi,et al /2012 | 259.4±68.5 | ET | C-index 0.89; 0.93 | unit | NA | 1,2,3 |
|  |  |  | Chien, et al 2008 | 0.324 (0.211-0.486) mmol/l | ET | HR 1.40 (1.02-1.92) | Top quintile | 0.027 | 1,2,3 |
|  |  |  | Krishnan E et al. 2012 | Mean (SD): 5.2 (1.4) (mg/dl) | uricase method | HR 2.69 (1.91-3.79) | Top quartile | Nr | 1,2,3 |
|  |  |  | Nan et al./2008 | M: 423, F: 311 μmol/l | ET | HR, M: 1.40 (1.27, 1.55), F: 1.23 (1.13, 1.35) | SD | M: <0.001, F; <0.05 | 2 |
|  |  |  | Rathmann, et al 2010 | 388.5±82.9 (T2D) vs 326.6±78.7 (non-case) μmol/l | uricase method | OR, 1.49 | 1-unit | 0.0001 | 1,2,3 |
|  |  |  | Sluijs et al./2013 | q1: 174, q2: 210, q3: 243, q4: 299 μmol/l | ET | HR, q1: 1.00, q2: 1.56 (1.17, 2.09), q3: 2.36 (1.77, 3.15), q4: 4.91 (3.67, 6.57) | Quartile | <0.001 | 1 |
|  |  |  | Wang et al./2011 | 314.9±97.8 μmol/l | Autoanalyser | HR, q1: 1.00 (ref.)/q2: 1.04 (0.49-2.22)/q3: 1.00 (0.67-1.49)/q4:1.57 (1.24-1.99) | Quartile | <0.001 | 1,2,3 |
| Oxidative status | F2-Isoprostanes | Novel | Il'yasova, et al 2012 | 0.14 (75th-25th) ng/mg creatinine | LC | OR 0.52 (0.39-0.67) | NR | <0.001 | 1,2,3 |
|  | Paraoxonase-1 activity | Novel | Salomaa 2010 | 92.3 (52.0-170.6) (nmol/min/mL) | Charlton-Menys V, Liu Y, Durrington PN. Semiautomated method for determination of serum paraoxonase activity using paraoxon as substrate. Clin Chem 2006; 52: 453-7. | NR (only graphically shown) | SD | 0.8439 | 1,2,3 |
|  | Urine isoprostanes | Novel | Dallmeier, et al 2012 | 147 (104-204) (T2D) VS 126 (87-186) (non-case) ng/mmol | EIA | OR 1.16 (0.92–1.44) | 1-SD for log-transformed | 0.21 | 1,2,3 |
| Renal function | Cystatin C | Novel | Sahakyan et al./2011 | 0.85 (0.75-0.99) mg/L | nephelometry | OR 2.19 (1.02-4.68) | log | NR | 1,2,3 |
|  | Cystatin C |  | Salomaa, et al 2010 | 0.8 (0.7-0.9) mg/L | LIA | NR (only graphically shown) | SD | 0.1818 | 1,2,3 |
|  | UAE single urine sample | Clinical | Halimi JM, et al 2008 | F: 9.5% 20-200, 1.3% >200/ M: 10.5% 20-200, 1.6% >200 mg/l | nephelometry | OR, 20-200 mg/l1.76 (1.07–2.88)/ >200 2.98 (1.28–6.93) | Cut-off values | 0.0257/ 0.01 | 1,2,3 |
|  | Urine albumin excretion (UAE) 24h | Clinical | Brantsma, et al/2005 | 14.6(8.7-36) (T2D) vs 8.9 (6.2-15.2) mg/24h | nephelometry | OR, 1.53 (1.25-1.88) | log | <0.001 | 1,2,3 |
|  | Urine albumin/creatinine ratio (ACR) | Clinical | Wang et al./2006 | 11.0 (7.8-15.6) mg/mmol | Beckman immunoassay | OR, 3.32 (1.61-6.85) | Tertile 3 | 0.004 | None |
| Sex hormone | Progesterone | Clinical | Nilsson et al./2009 | M: 1.8, F: 1.4 nmol/l | AutoDELFIA | OR, q1 vs q2-q3: 0.70, q4 vs q2-q3: 1.47 | Quartile | <0.05 | 1 |
|  | Genistein | Novel | Ko et al./2014 | M: 428±456 (T2D) vs. 414±466, F:337±385 (T2D) vs. 413±544 ng/ml | LC-MS | OR, M:1.15 (0.74-1.81), F:0.58 (0.35-0.95) | Top quartile | M:0.23, F:0.024 | 1,2,3 |
|  | Glycitein | Novel | Ko et al./2014 | M:12±12.7 (T2D) vs. 11.1±10.4, F:12.4±20.5 (T2D) vs. 10.6±8.5 ng/ml | LC-MS | OR, M:1.92 (0.82-4.47), F:1.24 (0.7-2.21) | Top quartile | M:0.28, F:0.54 | 1,2,3 |
|  | Diadzein | Novel | Ko et al./2014 | M:185±297 (T2D) vs. 198±730, F:180±753 (T2D) vs. 153±219 ng/ml | LC-MS | OR, M:1.10 (0.46-2.66), F:0.87 (0.47-1.61) | Top quartile | M:0.95, F:0.62 | 1,2,3 |
|  | Equol | Novel | Ko et al./2014 | M:120±329 (T2D) vs. 85±176, F:77.3±207 (T2D) vs. 97±292 ng/ml | LC-MS | OR, M:1.48 (0.65-3.38), F:1.01 (0.56-1.82) | Top quartile | M:0.20, F:0.98 | 1,2,3 |
| Skeletal muscle mass/renal function | serum creatinine | Clinical | Harita, et al 2009 | 0.4-1.60 mg/dl | ET | OR, 1.91 (1.44–2.54) | 0.4-0.6 mg/dl | NR | 1,2,3 |
|  |  |  | Salomaa, et al 2010 | 0.9 (0.8-1.0) | Kinetic Assay | NR (only graphically shown) | SD | 0.9899 | 1,2,3 |
| Stress-adaptation system | Copeptin (c-terminal pre pro-vasopressin) | Novel | Abbasi,et al /2012 | F:3.6 (2.4–5.5)/ M:6.2 (4.0–9.4) pg/ml | ELISA | OR, F: 1.49 (1.24-1.79)/M: 1.01 (0.85-1.19) | log2 | F: <0.001/M: 0.95 | 1,2,3 |
|  |  |  | Enhörning, et al/ 2013 | 5.08 (3.19–8.09) pmol/ l | chemiluminescence/coated tube format | OR, 1.18 (1.32-1.46) | Quartile | 0.04 | 1,2,3 |
|  |  |  | Salomaa, et al 2010 | 3.6 (2.1-6.1) | ILA | NR (only graphically shown) | SD | 0.1261 | 1,2,3 |
| Vascular damage | Cathepsin S | Novel | Jobs et al. 2013 | Mean (SD): 17.8 ug/L (3.8) | ELISA | OR 1.48 (1.08-2.01) | SD | 0.01 | 1,2,3 |
|  | CD 40 ligand | Novel | Dallmeier, et al 2012 | 1.21 (0.55-4.04) (T2D) vs 1.26 (0.54-4.03)(non-case) ng/ml | ELISA | OR 1.06 (0.88–1.28) | 1-SD for log-transformed | 0.51 | 1,2,3 |
|  | Lipoprotein-associated phospholipase A2 (LpaPA2) activity | Novel | Dallmeier, et al 2012 | 277 (216-357) (T2D) vs 282 (226-352) (non-case) (nmol/min/ml) | ET | OR 1.14 (0.92–1.42) | 1-SD for log-transformed | 0.24 | 1,2,3 |
|  | Lipoprotein-associated phospholipase A2 (LpaPA2) activity | | Salomaa 2010 | 231.6 (197.8-282.3) (nmol/min/ml) | CAM | NR (only graphically shown) | SD | 0.3073 | 1,2,3 |
|  | Lipoprotein-associated phospholipase A2 (LpaPA2) mass | Novel | Salomaa 2010 | 247.8 (213.9-288.4) (ng/ml) | ELISA Lp-PLA2 PLAC | NR (only graphically shown) | SD | 0.6986 | 1,2,3 |
|  | Lipoprotein-associated phospholipase A2 (LpaPA2) mass | | Dallmeier, et al 2012 | 324 (268-412) (T2D) vs 302 (245-371) (non-case) (ng/ml) | EA | OR 0.92 (0.76–1.12) | 1-SD for log-transformed | 0.41 | 1,2,3 |
|  | P-selectin | Novel | Dallmeier, et al 2012 | 5.21 (4.50-6.29) (T2D) vs 5.15 (4.25-6.13) (non-case) pg/ml | ELISA | OR 0.89 (0.74–1.07) | 1-SD for log-transformed | 0.21 | 1,2,3 |
|  | Soluble thrombomodulin (sTM) | Novel | Thorand et al./2007 | 4.7±0.12 ng/ml | ELISA | HR, 0.73 (0.58-0.91) | SD | 0.006 | 1,2,3 |
| Vascular/endothelial system | Placental growth factor | Novel | Salomaa 2010 | 14.8 (12.3-17.1) (pg/ml) | CMIA | NR (only graphically shown) | SD | 0.8113 | 1,2,3 |
| Vitamin | Vitamin B12 | Clinical | Salomaa 2010 | 74.7 (59.1-101.5) (pmol/l) | MEIA | NR (only graphically shown) | SD | 0.1198 | 1,2,3 |
|  | Vitamin B12 |  | Salomaa 2010 | 490.0 (386.0-637.0) (pg/ml) | CMIA | NR (only graphically shown) | SD | 0.9133 | 1,2,3 |
| Vitamin D status | 25-hydroxyvitamin D (25(OH)D) | Clinical | Grimnes G, et al/ 2010 | 52.8±16.8 (non-smoker) vs 73.0±20.3 (smoker) nmol/l | ECLIA | HR, non-smoker, 1.37 (0.89–2.10)/ smoker, 1.47 (0.62–3.48) | Quartile | 0.15/ 0.38 | 1,2 |
|  |  |  | Husemoen, et al/ 2012 | 48 (12-118) nmol/l | LC | OR 0.94 (0.86–1.03) | 10 nmol/L | 0.18 | 1,2 |
|  |  |  | Mattila et al/ 2007 | Quartile 1, <30 nmol/l; 2, 30 – 41 nmol/l; 3, 42–55 nmol/l; and 4, >55 nmol/l. | RIA | RR 0.58 (0.32–1.06) | Top quartile | 0.06 | 1,2 |
|  |  |  | Schöttker et al./2013 | 46.1 nmol/l | LC-MS/MS | HR, 1.35 (1.13-1.61) | Bottom quintile | <0.05 | 1, 2 |
|  |  |  | Schafer et al. 2014 | 23.0±10.9 ng/ml | LC-MS/MS | HR, 0.97 (0.86-1.11) | SD | NR | 1,2 |
|  |  |  |  |  |  |  |  |  |  |

| *Platform used for biomarker assay |  |
| --- | --- |
| Chemiluminescence | CL |
| [Fluorescence Polarization Immunoassay](http://www.boomer.org/c/p3/c03/c0309.html) | FPIA |
| Chemiluminescent immunometric assay | CLIMA |
| Chemiluminescent microparticle immunoassay | CMIA |
| Colorimetric activity assay CAM | CAM |
| Electrochemiluminescence sandwich immunoassay | ECLIA |
| Enzymatic technique | ET |
| Enzyme assay | EA |
| Enzyme immunoassay | EIA |
| Enzyme-linked immunosorbent assay | ELISA |
| [High-performance liquid chromatography](http://en.wikipedia.org/wiki/High-performance_liquid_chromatography) | HPLC |
| High-sensitivity immunoradiometric assay | hs-IRA |
| Immunoassay | IA |
| Immunoluminometric assay | ILA |
| Immunometric assay | IMA |
| Immunonephelometric assay | INA |
| Immunoturbidimetric assay | ITA |
| immunoturbidimetric latex | ITL |
| Latex immunoassay | LIA |
| latex-enhanced assay | LEA |
| Latex-enhanced immunonephelometric | LEINA |
| liquid chromatography/flow injection analysis-tandem mass spectrometry | LC/FIA-MS |
| liquid chromatography-tandem mass spectrometry | LC-MS/MS |
| Mass spectrometry | MS |
| Microparticle enzyme immunoassay | MEIA |
| Partilce-enhanced immunoturbidimetric | PEITA |
| Radioimmunoassay | RIA |
| Radioligand-binding assay | RLBA |
| Routine method | RT |
| Standard methods | SM |
| Turbidimetric inhibition immunoassay | TINIA |
| Ultra sensitive enzyme-linked immunosorbent assay | us-ELISA |

| †Multivariable adjustment: |
| --- |
| 1- age and sex |
| 2- non-invasive variables including BMI or waist circumference, family history of diabetes, smoking, hypertension |
| 3- glucose and other markers |
