# Supplementary material for: A Systematic Review of Biomarkers and Risk of Incident Type 2 Diabetes: An Overview of Epidemiological, Prediction and Aetiological Research Literature
Source: PLoS One. 2016 Oct 27;11(10):e0163721. doi: 10.1371/journal.pone.0163721 (PMC5082867; doi:10.1371/journal.pone.0163721)
Supplement: S4 Table — (DOC) [file pone.0163721.s008.doc]

**S4 Table. Associations of Biomarkers with Risk of Incident T2D by BMI Strata.**

| **Category** | **Biomarker** | **Study/Year** | **Biomarker level** | **Per unit/SD/pertentile** | **Adjustment*** | **Effect estimates stratified by BMI** |
| --- | --- | --- | --- | --- | --- | --- |
| Adipokine | Adiponectin | Heidemann, et al. 2008 | 16.4 (12.4–23.6) (T2D) vs 15.1 (11.5–21.2) (control) ng/ml | Quintile 5 | 1,2 | OR, BMI ≥25,1.09 (0.74–1.61) /BMI <25, 1.82 (0.99–3.35) |
|  | Adiponectin high-molecular-weight | Heidemann, et al./ 2008 | 11.4 (7.6–16.3) (T2D) vs 17.8 (12.9–22.7) (control) μg/ml | Quintile 5 | 1,2 | OR, BMI ≥25,0.19 (0.12–0.31) /BMI <25, 0.10 (0.05–0.20) |
|  | Resistin | Heidemann, et al./ 2008 | 3.6 (2.2–5.8) (T2D) vs 6.6 (4.4–9.8) (control) μg/ml | Quintile 5 | 1,2 | OR, BMI ≥25,0.11 (0.06–0.20) /BMI <25, 0.05 (0.02–0.12) |
| Fibrinolytic system | Plasminogen activator inhibitor-1 (PAI-1) | Festa, et al. 2002 | 24 (15–37.5) (T2D) vs 16 (9–27) (non-case) ng/ml | SD | 1,2,3 | OR, BMI ≥27.4,1.56 (1.18–2.08)/BMI < 27.4, 1.89 (1.30–2.75) |
| Glycemia | Insulin | Gautier, et al. 2010 | 35.7 (26.7-48.0)(BMI<27) vs 56.5 (42.4-79.5) (BMI≥27) pmol/l | log | 1,2,3 | HR, BMI <27, 1.12 (0.87–1.43)/ BMI≥27, 1.39 (1.07–1.80) |
| Inflammation | Fibrinogen | Festa, et al. 2002 | 2.40 (1.29–5.87) (T2D) vs 1.67 (0.75–3.41) (non-case) mg/l | SD | 1,2,3 | OR, BMI ≥27.4,1.04 (0.81–1.34)/BMI < 27.4, 1.46 (1.05–2.05) |
|  | C-reactive protein (CRP) | Festa, et al. 2002 | 287.8±58.8 (T2D) vs 275.1±56.1 (non-case) mg/dl | log | 1,2,3 | OR, BMI ≥27.4,0.98 (0.78–1.24) /BMI < 27.4, 1.34 (0.96–1.88) |
|  |  | Hu, et al. 2004 | 0.36 (T2D) vs 0.16 (non-case) mg/dl | Top quintile | 1,2 | RR, BMI ≥ 30, 3.38 (1.31-8.77)/ BMI < 30, 3.57 (2.13-5.96) |
|  |  | Hu, et al. 2004 | 2.38 (T2D) vs 1.84 (non-case) pg/ml | Top quintile | 1,2 | RR, BMI ≥ 30, 1.46 (0.57–3.76)/ BMI < 30, 1.84 (1.16–2.94) |
|  | Interleukin 6 (IL-6) | Hu, et al. 2004 | 2646.5 (T2D) vs 2383.8 (non-case) pg/ml | Top quintile | 1,2 | RR, BMI ≥ 30, 0.93 (0.41–2.09)/ BMI < 30, 2.25 (1.39–3.62) |
|  |  | Møller, et al. 2011 | F: 1.71(1.31-2.26)/ M: 1.76(1.37-2.36) mg/l | log | 1,2 | AR: M: BMI> 25 7-36%/F: BMI>25 4-19% |
|  | soluble CD163 | Pradhan, et al. 2001 | 0.69 (0.42-1.0) (T2D) vs 0.26 (0.1-0.61) (non-case) mg/dl | Top quartile | 1,2,3 | RR, BMI ≥ 29, 5.3/ BMI < 29, 5.6 |
|  | Tumor necrosis factor α receptor 2 (TNF-α–R2) | Pradhan, et al. 2001 | 2 (1.43-2.78) (T2D) vs (1.38 (0.91-2.05) (non-case) pg/ml | Top quartile | 1,2,3 | RR, BMI ≥ 29, 3/ BMI < 29, 2.6 |
|  | Interleukin-18 (IL-18) | Thorand et al. 2005 | 188.7 pg/ml | Top quartile | 1,2,3 | HR, BMI <25, 2.3/BMI 25-30 1.5/ BMI≥30 1.84 |
| Lipid | high density lipoprotein cholesterol (HDL cholesterol) | Gautier, et al. 2010 | 1.65 (1.39-1.96)(BMI<27) vs 1.44 (1.19, 1.70) (BMI≥27) mmol/l | SD | 1,2,3 | HR, BMI <27, 0.89 (0.68–1.15)/ BMI≥27, 0.77 (0.58–1.02) |
|  | Triacylglycerol | Gautier, et al. 2010 | 0.88 (0.63, 1.21)(BMI<27) vs 1.25 (0.90, 1.82)(BMI≥27) mmol/l | SD | 1,2,3 | HR, BMI <27, 1.34 (1.06–1.69)/ BMI≥27, 1.41 (1.10–1.81) |
|  |  | Hjellvik, et al. 2012 | F: 2.4 (T2D) vs 1.3 (non-case)/ M: 3.2 (T2D) vs 2.1 (non-case) mmol/l | Quantile | 1,2,3 | RR, F: BMI≤24.1, 4.4 (1.9-10.2)/ BMI≥30.2, 1.2 (0.7-2.1)/ M: BMI≤25.9, 2.8 (1.5–5.2), BMI ≥30.5, 2.2 (1.4–3.6) |
| Liver function | Alanine aminotransferase (ALT) | André, etal/ 2005 | F:22.2±21.3/M:41.2±40.1U/l | Top quintile | 1,2,3 | OR, BMI< 25, 2.7 (1.0-7.3)/ BMI ≥25, 3.4 (1.1-9.6) |
|  |  | Choi, et al. 2013 | 17.1±5.4 (control) to 45.9±20.8 (subjects with↑ALT and steatosis) | >30 IU/L | 1,2,3 | HR, BMI ≥24.3,1.71 (1.10-2.65) |
|  |  | Doi Y, et al. 2007 | F: 19 (12-33)/ M:22 (14-45) | Top quintile | 1,2,3 | OR, BMI ≥24.3, 1.72 (0.97-3.03) |
|  | Asparate aminotransferase (AST) | Doi Y, et al. 2007 | F: 13 (8-35)/ M:22 (11-95) U/l | Top quintile | 1,2,3 | OR, BMI ≥24.3, 1.99 (1.32-3.00) |
|  | γ-glutamyltranspeptidase (GGT) | Gautier, et al. 2010 | 19.9 (15.0-26.9)(BMI<27) vs 26.8 (19.9-37.7) (BMI≥27) | SD | 1,2,3 | HR, BMI <27, 1.34 (1.08–1.68)/ BMI≥27, 1.37 (1.06–1.77) |
|  |  | Gautier, et al. 2010 | 19.7 (14.7-29.7) (BMI<27) vs 29.4 (19.7-46.2) (BMI≥27) | SD | 1,2,3 | HR, BMI <27, 1.59 (1.29–1.97)/  BMI≥27,1.07 (0.82–1.38) |
|  |  | Goessling W, et al. 2008 | 24±14 U/l | SD | 1,2,3 | OR, P for BMI-interaction=0.01 |
|  |  | Lee et al.. 2003 | NR | > 50 U/L | 1,2,3 | RR, Interaction with BMI reported |
| oxidative status | F2-Isoprostanes | Il'yasova, et al. 2012 | 0.14 (75th-25th) ng/mg creatinine | NR | 1,2,3 | OR, BMI ≥ 30, 0.37 (0.23–0.56)/ BMI < 30, 0.66 (0.45–0.91) |
| skeletal muscle mass/renal function | serum creatinine | Harita, et al. 2009 | 0.4-1.60 mg/dl | 0.4-0.6 mg/dl | 1,2,3 | OR, BMI ≤23.3, 1.96 (1.28-2.98)/ BMI > 23.3, 1.85 (1.26-2.73) |
| Vitamin D status | 25-hydroxyvitamin D (25(OH)D) | Grimnes G, et al./ 2010 | 52.8±16.8 (non-smoker) vs 73.0±20.3 (smoker) nmol/l | Quartile | 1,2 | HR, non-smoker, BMI < 23.1, 0.67 (0.42-1.07)/  smoker, BMI <23.1, 0.64 (0.42-0.98) |
|  |  | Husemoen, et al./ 2012 | 48 (12-118) nmol/l | 10 nmol/L | 1,2 | OR, BMI ≥25,0.92 (0.85-1.01) /BMI <25, 0.90 (0.77-1.06) |
