# Supplementary material for: A Systematic Review of Biomarkers and Risk of Incident Type 2 Diabetes: An Overview of Epidemiological, Prediction and Aetiological Research Literature
Source: PLoS One. 2016 Oct 27;11(10):e0163721. doi: 10.1371/journal.pone.0163721 (PMC5082867; doi:10.1371/journal.pone.0163721)
Supplement: S5 Table — (DOC) [file pone.0163721.s009.doc]

**S5 Table. 17 Mendelian Randomisation Studies of the Identified Biomarkers**

| **Title** | **Description** | **Details** | **Short details** | **PMID** | **Biomarker** |
| --- | --- | --- | --- | --- | --- |
| Bilirubin as a potential causal factor in type 2 diabetes risk: a Mendelian randomization study. | Abbasi A, et al | Diabetes. 2014 Nov 3. doi:pii: DB_140228. [Epub ahead of print] | Diabetes. 2014 | 25368098 | Bilirubin |
| The Causal Effect of Vitamin D Binding Protein (DBP) Levels on Calcemic and Cardiometabolic Diseases: A Mendelian Randomization Study. | Leong A, et al | PLoS Med. 2014 Oct;11(10):e1001751. doi: 10.1371/journal.pmed.1001751. | PLoS Med. 2014 | 25350643 | VitDBP |
| Association between circulating 25-hydroxyvitamin D and incident type 2 diabetes: a mendelian randomisation study. | Ye Z, et al | Lancet Diabetes Endocrinol. 2014 Sep 30. doi:pii: S2213-8587(14)70184-6. 10.1016/S2213-8587(14)70184-6. [Epub ahead of print] | Lancet Diabetes Endocrinol. 2014 | 25281353 | VitD |
| Associations of common variants in methionine metabolism pathway genes with plasma homocysteine and the risk of type 2 diabetes in han chinese. | Huang T, et al | J Nutrigenet Nutrigenomics. 2014;7(2):63-74. doi: 10.1159/000365007. Epub 2014 Jul 25. | J Nutrigenet Nutrigenomics. 2014 | 25074646 | homocysteine |
| Genetic evidence for a normal-weight metabolically obese" phenotype linking insulin resistance | Yaghootkar H, et al | Diabetes. 2014 Jul 21. doi:pii: DB_140318. [Epub ahead of print] | Diabetes. 2014 | 25048195 | IR |
| Lipoprotein(a) concentrations, isoform size, and risk of type 2 diabetes: a Mendelian randomisation study. | Kamstrup PR, Nordestgaard BG | Lancet Diabetes Endocrinol. 2013 Nov;1(3):220-7. doi: 10.1016/S2213-8587(13)70064-0. Epub 2013 Aug 27. | Lancet Diabetes Endocrinol. 2013 | 24622370 | Lpa |
| Association of homocysteine with type 2 diabetes: a meta-analysis implementing Mendelian randomization approach. | Huang T, et al | BMC Genomics. 2013 Dec 10;14:867. doi: 10.1186/1471-2164-14-867. | BMC Genomics. 2013 | 24320691 | homocysteine |
| Common quantitative trait locus downstream of RETN gene identified by genome-wide association study is associated with risk of type 2 diabetes mellitus in Han Chinese: a Mendelian randomization effect. | Chung CM, et al | Diabetes Metab Res Rev. 2014 Mar;30(3):232-40. doi: 10.1002/dmrr.2481. | Diabetes Metab Res Rev. 2014 | 24123702 | resistin |
| The association between circulating lipoprotein(a) and type 2 diabetes: is it causal? | Ye Z, et al | Diabetes. 2014 Jan;63(1):332-42. doi: 10.2337/db13-1144. Epub 2013 Oct 2. | Diabetes. 2014 | 24089516 | Lpa |
| Plasma 25-hydroxyvitamin D and its genetic determinants in relation to incident type 2 diabetes: a prospective case-cohort study. | Buijsse B, et al | Eur J Epidemiol. 2013 Sep;28(9):743-52. doi: 10.1007/s10654-013-9844-5. Epub 2013 Sep 4. | Eur J Epidemiol. 2013 | 24002339 | VitD |
| Mendelian randomization studies do not support a causal role for reduced circulating adiponectin levels in insulin resistance and type 2 diabetes. | Yaghootkar H, et al | Diabetes. 2013 Oct;62(10):3589-98. doi: 10.2337/db13-0128. Epub 2013 Jul 8. Review. | Diabetes. 2013 | 23835345 | adiponectin |
| Genetically elevated fetuin-A levels, fasting glucose levels, and risk of type 2 diabetes: the cardiovascular health study. | Jensen MK, et al | Diabetes Care. 2013 Oct;36(10):3121-7. doi: 10.2337/dc12-2323. Epub 2013 Jun 25. | Diabetes Care. 2013 | 23801724 | fetuin-a |
| A comprehensive investigation of variants in genes encoding adiponectin (ADIPOQ) and its receptors (ADIPOR1/R2), and their association with serum adiponectin, type 2 diabetes, insulin resistance and the metabolic syndrome. | Peters KE, et al | BMC Med Genet. 2013 Jan 25;14:15. doi: 10.1186/1471-2350-14-15. | BMC Med Genet. 2013 | 23351195 | adiponectin |
| Pancreatic beta-cell function and type 2 diabetes risk: quantify the causal effect using a Mendelian randomization approach based on meta-analyses. | Song Y, et al | Hum Mol Genet. 2012 Nov 15;21(22):5010-8. doi: 10.1093/hmg/dds339. Epub 2012 Aug 29. | Hum Mol Genet. 2012 | 22936689 | beta-cell |
| A prospective study of leukocyte telomere length and risk of type 2 diabetes in postmenopausal women. | You NC, et al | Diabetes. 2012 Nov;61(11):2998-3004. doi: 10.2337/db12-0241. Epub 2012 Jul 24. | Diabetes. 2012 | 22829448 | leukocytr telomere length |
| Polymorphisms related to the serum 25-hydroxyvitamin D level and risk of myocardial infarction, diabetes, cancer and mortality. The TromsÃ¸ Study. | Jorde R, et al | PLoS One. 2012;7(5):e37295. doi: 10.1371/journal.pone.0037295. Epub 2012 May 23. | PLoS One. 2012 | 22649517 | VitD |
| Novel loci for adiponectin levels and their influence on type 2 diabetes and metabolic traits: a multi-ethnic meta-analysis of 45,891 individuals. | Dastani Z, et al | PLoS Genet. 2012;8(3):e1002607. doi: 10.1371/journal.pgen.1002607. Epub 2012 Mar 29. | PLoS Genet. 2012 | 22479202 | adiponectin |
| Association of TMPRSS6 polymorphisms with ferritin, hemoglobin, and type 2 diabetes risk in a Chinese Han population. | Gan W, et al | Am J Clin Nutr. 2012 Mar;95(3):626-32. doi: 10.3945/ajcn.111.025684. Epub 2012 Feb 1. | Am J Clin Nutr. 2012 | 22301935 | ferritin/TMPRSS6 |
| Mendelian randomization study of B-type natriuretic peptide and type 2 diabetes: evidence of causal association from population studies. | Pfister R, et al | PLoS Med. 2011 Oct;8(10):e1001112. doi: 10.1371/journal.pmed.1001112. Epub 2011 Oct 25. | PLoS Med. 2011 | 22039354 | BNP |
| Genome-wide association identifies nine common variants associated with fasting proinsulin levels and provides new insights into the pathophysiology of type 2 diabetes. | Strawbridge RJ, et al | Diabetes. 2011 Oct;60(10):2624-34. doi: 10.2337/db11-0415. Epub 2011 Aug 26. | Diabetes. 2011 | 21873549 | proinsulin |
| No evidence for a causal link between uric acid and type 2 diabetes: a Mendelian randomisation approach. | Pfister R, et al | Diabetologia. 2011 Oct;54(10):2561-9. doi: 10.1007/s00125-011-2235-0. Epub 2011 Jun 30. | Diabetologia. 2011 | 21717115 | uric acid |
| Mendelian randomization studies do not support a role for raised circulating triglyceride levels influencing type 2 diabetes, glucose levels, or insulin resistance. | De Silva NM, et al | Diabetes. 2011 Mar;60(3):1008-18. doi: 10.2337/db10-1317. Epub 2011 Jan 31. | Diabetes. 2011 | 21282362 | triglyceride |
| Erythrocyte membrane phospholipid fatty acids, desaturase activity, and dietary fatty acids in relation to risk of type 2 diabetes in the European Prospective Investigation into Cancer and Nutrition (EPIC)-Potsdam Study. | Kröger J, et al | Am J Clin Nutr. 2011 Jan;93(1):127-42. doi: 10.3945/ajcn.110.005447. Epub 2010 Oct 27. | Am J Clin Nutr. 2011 | 20980488 | delta-6 desaturase |
| Genetic evidence that raised sex hormone binding globulin (SHBG) levels reduce the risk of type 2 diabetes. | Perry JR, et al | Hum Mol Genet. 2010 Feb 1;19(3):535-44. doi: 10.1093/hmg/ddp522. Epub 2009 Nov 18. | Hum Mol Genet. 2010 | 19933169 | SHBG |
| Circulating beta-carotene levels and type 2 diabetes-cause or effect? | Perry JR, et al | Diabetologia. 2009 Oct;52(10):2117-21. doi: 10.1007/s00125-009-1475-8. Epub 2009 Aug 7. | Diabetologia. 2009 | 19662379 | beta-carotene |
| Sex hormone-binding globulin and risk of type 2 diabetes in women and men. | Ding EL, et al | N Engl J Med. 2009 Sep 17;361(12):1152-63. doi: 10.1056/NEJMoa0804381. Epub 2009 Aug 5. | N Engl J Med. 2009 | 19657112 | SHBG |
| Inflammation, insulin resistance, and diabetes--Mendelian randomization using CRP haplotypes points upstream. | Brunner EJ, et al | PLoS Med. 2008 Aug 12;5(8):e155. doi: 10.1371/journal.pmed.0050155. | PLoS Med. 2008 | 18700811 | CRP |
| Effect of macrophage migration inhibitory factor (MIF) gene variants and MIF serum concentrations on the risk of type 2 diabetes: results from the MONICA/KORA Augsburg Case-Cohort Study, 1984-2002. | Herder C, et al | Diabetologia. 2008 Feb;51(2):276-84. Epub 2007 Aug 22. | Diabetologia. 2008 | 17712545 | Macrophage migration inhibitory factor |
| PPARG2 Pro12Ala and ADAMTS9 rs4607103 as insulin resistance loci" and "insulin secretion loci" in Italian individuals. The GENFIEV study and the Verona Newly Diagnosed Type 2 Diabetes Study (VNDS) 4." | Trombetta M, et al | Acta Diabetol. 2013 Jun;50(3):401-8. doi: 10.1007/s00592-012-0443-9. Epub 2012 Nov 17. | Acta Diabetol. 2013 | 23161442 | HOMA-IR/beta cell |
