# Supplementary material for: A Systematic Review of Biomarkers and Risk of Incident Type 2 Diabetes: An Overview of Epidemiological, Prediction and Aetiological Research Literature
Source: PLoS One. 2016 Oct 27;11(10):e0163721. doi: 10.1371/journal.pone.0163721 (PMC5082867; doi:10.1371/journal.pone.0163721)
Supplement: S6 Table — (DOC) [file pone.0163721.s010.doc]

**S6 Table. 20 Meta-analysis Studies of the Identified Biomarkers**

| **Title** | **Description** | **Details** | **Short Details** | **OMID** | **Biomarkers** |
| --- | --- | --- | --- | --- | --- |
| Gamma-glutamyl transferase and risk of type II diabetes: an updated systematic review and dose-response meta-analysis. | Kunutsor SK, et al | Ann Epidemiol. 2014 Sep 8. doi:pii: S1047-2797(14)00401-3. 10.1016/j.annepidem.2014.09.001. [Epub ahead of print] | Ann Epidemiol. 2014 | 25263236 | GGT |
| Association of iron indices and type 2 diabetes: a meta-analysis of observational studies. | Orban E, et al | Diabetes Metab Res Rev. 2014 Jul;30(5):372-94. doi: 10.1002/dmrr.2506. | Diabetes Metab Res Rev. 2014 | 24327370 | ferritin, |
| Association of homocysteine with type 2 diabetes: a meta-analysis implementing Mendelian randomization approach. | Huang T, et al | BMC Genomics. 2013 Dec 10;14:867. doi: 10.1186/1471-2164-14-867. | BMC Genomics. 2013 | 24320691 | leptin |
| Liver aminotransferases and risk of incident type 2 diabetes: a systematic review and meta-analysis. | Kunutsor SK, et al | Am J Epidemiol. 2013 Jul 15;178(2):159-71. doi: 10.1093/aje/kws469. Epub 2013 May 31. Review. | Am J Epidemiol. 2013 | 23729682 | ALT, AST |
| Blood 25-hydroxy vitamin D levels and incident type 2 diabetes: a meta-analysis of prospective studies. | Song Y, et al | Diabetes Care. 2013 May;36(5):1422-8. doi: 10.2337/dc12-0962. | Diabetes Care. 2013 | 23613602 | Vitamin D |
| Serum uric acid levels and incidence of impaired fasting glucose and type 2 diabetes mellitus: A meta-analysis of cohort studies. | Jia Z, et al | Diabetes Res Clin Pract. 2013 Jul;101(1):88-96. doi: 10.1016/j.diabres.2013.03.026. Epub 2013 Apr 19. | Diabetes Res Clin Pract. 2013 | 23608549 | uric acid |
| High serum uric acid and increased risk of type 2 diabetes: a systemic review and meta-analysis of prospective cohort studies. | Lv Q, et al | PLoS One. 2013;8(2):e56864. doi: 10.1371/journal.pone.0056864. Epub 2013 Feb 20. Review. | PLoS One. 2013 | 23437258 | uric acid |
| Ferritin levels and risk of type 2 diabetes mellitus: an updated systematic review and meta-analysis of prospective evidence. | Kunutsor SK, et al | Diabetes Metab Res Rev. 2013 May;29(4):308-18. doi: 10.1002/dmrr.2394. Review. | Diabetes Metab Res Rev. 2013 | 23381919 | ferritin |
| Inflammatory markers and risk of type 2 diabetes: a systematic review and meta-analysis. | Wang X, et al | Diabetes Care. 2013 Jan;36(1):166-75. doi: 10.2337/dc12-0702. Review. | Diabetes Care. 2013 | 23264288 | IL-6, CRP |
| Low 25-hydroxyvitamin D and risk of type 2 diabetes: a prospective cohort study and metaanalysis. | Afzal S, et al | Clin Chem. 2013 Feb;59(2):381-91. doi: 10.1373/clinchem.2012.193003. Epub 2012 Dec 11. | Clin Chem. 2013 | 23232064 | Vitamin D |
| Vitamin D, type 2 diabetes and other metabolic outcomes: a systematic review and meta-analysis of prospective studies. | Khan H, et al | Proc Nutr Soc. 2013 Feb;72(1):89-97. doi: 10.1017/S0029665112002765. Epub 2012 Oct 30. Review. | Proc Nutr Soc. 2013 | 23107484 | Vitamin D |
| Body iron stores and heme-iron intake in relation to risk of type 2 diabetes: a systematic review and meta-analysis. | Zhao Z, et al | PLoS One. 2012;7(7):e41641. doi: 10.1371/journal.pone.0041641. Epub 2012 Jul 26. Review. | PLoS One. 2012 | 22848554 | ferritin |
| Omega-3 fatty acids and incident type 2 diabetes: a systematic review and meta-analysis. | Wu JH, et al | Br J Nutr. 2012 Jun;107 Suppl 2:S214-27. doi: 10.1017/S0007114512001602. Review. | Br J Nutr. 2012 | 22591895 | EPA+DHA, ALA |
| Circulating 25-hydroxyvitamin D concentration and the risk of type 2 diabetes: results from the European Prospective Investigation into Cancer (EPIC)-Norfolk cohort and updated meta-analysis of prospective studies. | Forouhi NG, et al | Diabetologia. 2012 Aug;55(8):2173-82. doi: 10.1007/s00125-012-2544-y. Epub 2012 Apr 15. Review. | Diabetologia. 2012 | 22526608 | Vitamin D |
| Mendelian randomization study of B-type natriuretic peptide and type 2 diabetes: evidence of causal association from population studies. | Pfister R, et al | PLoS Med. 2011 Oct;8(10):e1001112. doi: 10.1371/journal.pmed.1001112. Epub 2011 Oct 25. | PLoS Med. 2011 | 22039354 | NT-proBNP |
| Levels of vitamin D and cardiometabolic disorders: systematic review and meta-analysis. | Parker J, et al | Maturitas. 2010 Mar;65(3):225-36. doi: 10.1016/j.maturitas.2009.12.013. Epub 2009 Dec 23. Review. | Maturitas. 2010 | 20031348 | Vitamin D |
| Adiponectin levels and risk of type 2 diabetes: a systematic review and meta-analysis. | Li S, et al | JAMA. 2009 Jul 8;302(2):179-88. doi: 10.1001/jama.2009.976. Review. | JAMA. 2009 | 19584347 | adponectin |
| Association between serum uric acid and development of type 2 diabetes. | Kodama S, et al | Diabetes Care. 2009 Sep;32(9):1737-42. doi: 10.2337/dc09-0288. Epub 2009 Jun 23. | Diabetes Care. 2009 | 19549729 | uric acid |
| Association of C-reactive protein with type 2 diabetes: prospective analysis and meta-analysis. | Lee CC, et al | Diabetologia. 2009 Jun;52(6):1040-7. doi: 10.1007/s00125-009-1338-3. Epub 2009 Mar 27. | Diabetologia. 2009 | 19326095 | CRP |
| Alanine aminotransferase, gamma-glutamyltransferase, and incident diabetes: the British Women's Heart and Health Study and meta-analysis. | Fraser A, et al | Diabetes Care. 2009 Apr;32(4):741-50. doi: 10.2337/dc08-1870. Epub 2009 Jan 8. | Diabetes Care. 2009 | 19131466 | ALT, GGT |
