# Supplementary material for: A Systematic Review of Biomarkers and Risk of Incident Type 2 Diabetes: An Overview of Epidemiological, Prediction and Aetiological Research Literature
Source: PLoS One. 2016 Oct 27;11(10):e0163721. doi: 10.1371/journal.pone.0163721 (PMC5082867; doi:10.1371/journal.pone.0163721)
Supplement: S8 Table — (DOC) [file pone.0163721.s012.doc]

**S8 Table. 51 Prediction Studies of the Identified Biomarkers**

| **Title** | **Description** | **Details** | **Short details** | **PMID** | **Biomarkers** |
| --- | --- | --- | --- | --- | --- |
| Modelling of OGTT curve identifies 1 h plasma glucose level as a strong predictor of incident type 2 diabetes: results from two prospective cohorts. | Alyass A et al | Diabetologia. 2014 Oct 8. [Epub ahead of print] | Diabetologia. 2014 | 25292440 | Glucose (1h) |
| Relationships between adipokines, biomarkers of endothelial function and inflammation and risk of type 2 diabetes. | Julia C, et al | Diabetes Res Clin Pract. 2014 Aug;105(2):231-8. doi: 10.1016/j.diabres.2014.05.001. Epub 2014 May 17. | Diabetes Res Clin Pract. 2014 | 24931702 | adiponectin, leptin e-selectin MCP-1 sICAM sVCAM CRP |
| Fructosamine and glycated albumin for risk stratification and prediction of incident diabetes and microvascular complications: a prospective cohort analysis of the Atherosclerosis Risk in Communities (ARIC) study. | Selvin E, et al | Lancet Diabetes Endocrinol. 2014 Apr;2(4):279-88. doi: 10.1016/S2213-8587(13)70199-2. Epub 2014 Jan 15. | Lancet Diabetes Endocrinol. 2014 | 24703046 | Fructosamine, HbA1c |
| Use of high-normal levels of haemoglobin A(1C) and fasting plasma glucose for diabetes screening and for prediction: a meta-analysis. | Kodama S, et al | Diabetes Metab Res Rev. 2013 Nov;29(8):680-92. doi: 10.1002/dmrr.2445. Review. | Diabetes Metab Res Rev. 2013 | 23963843 | HbA1c (5.7%), Glucose (5.6mmol/l) |
| Do non-glycaemic markers add value to plasma glucose and hemoglobin a1c in predicting diabetes? Yuport health checkup center study. | Kashima S,et al | PLoS One. 2013 Jun 20;8(6):e66899. doi: 10.1371/journal.pone.0066899. Print 2013. | PLoS One. 2013 | 23818970 | Glucose, HbA1c Triglycerides LDL HDL AST ALT GGT WBC uric acid Creatinine |
| Metabolic syndrome is a poor predictor of diabetes in a Japanese health screening population. | Oda E, Aizawa Y | Intern Med. 2013;52(7):721-5. Epub 2013 Apr 1. | Intern Med. 2013 | 23545665 | Glucose, HbA1c |
| Liver function tests and risk prediction of incident type 2 diabetes: evaluation in two independent cohorts. | Abbasi A, et al | PLoS One. 2012;7(12):e51496. doi: 10.1371/journal.pone.0051496. Epub 2012 Dec 17. | PLoS One. 2012 | 23284703 | GGT, ALT, AST, albumin |
| Adipocytokines, hepatic and inflammatory biomarkers and incidence of type 2 diabetes. the CoLaus study. | Marques-Vidal P, et al | PLoS One. 2012;7(12):e51768. doi: 10.1371/journal.pone.0051768. Epub 2012 Dec 12. | PLoS One. 2012 | 23251619 | IL-1B, IL-6, TNF-a, CRP, leptin, adiponectin |
| Addition of inflammatory biomarkers did not improve diabetes prediction in the community: the framingham heart study. | Dallmeier D, et al | J Am Heart Assoc. 2012 Aug;1(4):e000869. doi: 10.1161/JAHA.112.000869. Epub 2012 Aug 24. | J Am Heart Assoc. 2012 | 23130155 | CRP, CD40 fibrinogen ICAM IL-6 urine isoprostanes LpaPA2 mass LpaPA2 activity MCP-1 P-selectin osteoprotegerin TNFR2 |
| Glycated haemoglobin and the risk of cardiovascular disease, diabetes and all-cause mortality in the Copenhagen City Heart Study. | Eskesen K, et al | J Intern Med. 2013 Jan;273(1):94-101. doi: 10.1111/j.1365-2796.2012.02594.x. Epub 2012 Nov 1. | J Intern Med. 2013 | 23009556 | HbA1c |
| Novel risk factors and the prediction of type 2 diabetes in the Atherosclerosis Risk in Communities (ARIC) study. | Raynor LA, et al | Diabetes Care. 2013 Jan;36(1):70-6. doi: 10.2337/dc12-0609. Epub 2012 Aug 28. | Diabetes Care. 2013 | 22933437 | WBC, fibrinogen albumin vWF aPTT factor VIII magnesium adiponectin leptin GGT ALT AST fetuin a ferritin lactate CRP oxidized LDL ICAM-1 sialic acid IL-6 IL-18 Complement C3 RBP-4  Asymmetric dimethylarginine (ADMA) |
| A risk score for predicting the incidence of type 2 diabetes in a middle-aged Korean cohort: the Korean genome and epidemiology study. | Lim NK, et al | Circ J. 2012;76(8):1904-10. Epub 2012 May 28. | Circ J. 2012 | 22640983 | HbA1c |
| Combined use of serum adiponectin and tumor necrosis factor-alpha receptor 2 levels was comparable to 2-hour post-load glucose in diabetes prediction. | Woo YC, et al | PLoS One. 2012;7(5):e36868. doi: 10.1371/journal.pone.0036868. Epub 2012 May 16. | PLoS One. 2012 | 22615828 | Glucose(2h), insulin adiponectin TNF-a R2 A-FABP CRP IL-6 |
| A two-step screening, measurement of HbA1c in association with FPG, may be useful in predicting diabetes. | Nomura K, et al | PLoS One. 2012;7(4):e36309. doi: 10.1371/journal.pone.0036309. Epub 2012 Apr 27. | PLoS One. 2012 | 22558430 | Glucose, HbA1c |
| Sex differences in the association between plasma copeptin and incident type 2 diabetes: the Prevention of Renal and Vascular Endstage Disease (PREVEND) study. | Abbasi A, et al | Diabetologia. 2012 Jul;55(7):1963-70. doi: 10.1007/s00125-012-2545-x. Epub 2012 Apr 15. | Diabetologia. 2012 | 22526609 | Copeptin |
| Screening for pre-diabetes to predict future diabetes using various cut-off points for HbA(1c) and impaired fasting glucose: the Toranomon Hospital Health Management Center Study 4 (TOPICS 4). | Heianza Y, et al | Diabet Med. 2012 Sep;29(9):e279-85. doi: 10.1111/j.1464-5491.2012.03686.x. | Diabet Med. 2012 | 22510023 | Glucose, HbA1c |
| Haemoglobin A1c cut-off point to identify a high risk group of future diabetes: results from the Omiya MA Cohort Study. | Kato M, et al | Diabet Med. 2012 Jul;29(7):905-10. doi: 10.1111/j.1464-5491.2012.03572.x. | Diabet Med. 2012 | 22248349 | Glucose, HbA1c |
| Predictive value of HbA1c for incident diabetes among subjects with impaired glucose tolerance--analysis of the Indian Diabetes Prevention Programmes. | Ramachandran A, et al | Diabet Med. 2012 Jan;29(1):94-8. doi: 10.1111/j.1464-5491.2011.03392.x. | Diabet Med. 2012 | 21790773 | HbA1c |
| HbA1c, fasting plasma glucose and the prediction of diabetes: Inter99, AusDiab and D.E.S.I.R. | Soulimane S, et al | Diabetes Res Clin Pract. 2012 Jun;96(3):392-9. doi: 10.1016/j.diabres.2011.06.003. Epub 2011 Jul 7. | Diabetes Res Clin Pract. 2012 | 21741107 | Glucose, HbA1c |
| Cut-off values of fasting and post-load plasma glucose and HbA1c for predicting Type 2 diabetes in community-dwelling Japanese subjects: the Hisayama Study. | Mukai N, et al | Diabet Med. 2012 Jan;29(1):99-106. doi: 10.1111/j.1464-5491.2011.03378.x. | Diabet Med. 2012 | 21726278 | Glucose, HbA1c |
| Two risk score models for predicting incident Type 2 diabetes in Japan. | Doi Y, et al | Diabet Med. 2012 Jan;29(1):107-14. doi: 10.1111/j.1464-5491.2011.03376.x. | Diabet Med. 2012 | 21718358 | Glucose |
| Plasma procalcitonin and risk of type 2 diabetes in the general population. | Abbasi A, et al | Diabetologia. 2011 Sep;54(9):2463-5. doi: 10.1007/s00125-011-2216-3. Epub 2011 Jun 15. No abstract available. | Diabetologia. 2011 | 21674177 | Procalcitonin, CRP |
| Immunological and cardiometabolic risk factors in the prediction of type 2 diabetes and coronary events: MONICA/KORA Augsburg case-cohort study. | Herder C, et al | PLoS One. 2011;6(6):e19852. doi: 10.1371/journal.pone.0019852. Epub 2011 Jun 6. | PLoS One. 2011 | 21674000 | CRP, IL-6 IL-18 TGF-B1 MIF MCP-1 IL-8 IP-10 leptin sE-selectin sICAM-1 |
| Biomarkers for the prediction of type 2 diabetes and cardiovascular disease. | Herder C, et al | Clin Pharmacol Ther. 2011 Jul;90(1):52-66. doi: 10.1038/clpt.2011.93. Epub 2011 Jun 8. Review. | Clin Pharmacol Ther. 2011 | 21654741 | RANTES, adiponectin |
| Identifying individuals at high risk for diabetes: The Atherosclerosis Risk in Communities study. | Schmidt MI, et al | Diabetes Care. 2005 Aug;28(8):2013-8. | Diabetes Care. 2005 | 16043747 | Glucose |
| Serum uric acid associates with the incidence of type 2 diabetes in a prospective cohort of middle-aged and elderly Chinese. | Wang T, et al | Endocrine. 2011 Aug;40(1):109-16. doi: 10.1007/s12020-011-9449-2. Epub 2011 Mar 23. | Endocrine. 2011 | 21431449 | uric acid |
| Metabolite profiles and the risk of developing diabetes. | Wang TJ, et al | Nat Med. 2011 Apr;17(4):448-53. doi: 10.1038/nm.2307. Epub 2011 Mar 20. | Nat Med. 2011 | 21423183 | Isoleucine, phenylalanine, tyrosine, valaine, leucine |
| The lack of utility of circulating biomarkers of inflammation and endothelial dysfunction for type 2 diabetes risk prediction among postmenopausal women: the Women's Health Initiative Observational Study. | Chao C, et al | Arch Intern Med. 2010 Sep 27;170(17):1557-65. doi: 10.1001/archinternmed.2010.312. | Arch Intern Med. 2010 | 20876407 | WBC, TNF-a R2 IL-6 CRP e-selectin ICAM-1 VCAM-1 |
| Prediction models for incident type 2 diabetes mellitusâ€¨in the older population: KORA S4/F4 cohort study. | Rathmann W, et al | Diabet Med. 2010 Oct;27(10):1116-23. doi: 10.1111/j.1464-5491.2010.03065.x. | Diabet Med. 2010 | 20854378 | Glucose, HbA1c, uric acid |
| Fasting plasma glucose and 5-year incidence of diabetes in the JPHC diabetes study - suggestion for the threshold for impaired fasting glucose among Japanese. | Noda M, et al | Endocr J. 2010;57(7):629-37. Epub 2010 May 28. | Endocr J. 2010 | 20508383 | Glucose |
| Plasma copeptin and the risk of diabetes mellitus. | Enhörning S, et al | Circulation. 2010 May 18;121(19):2102-8. doi: 10.1161/CIRCULATIONAHA.109.909663. Epub 2010 May 3. | Circulation. 2010 | 20439785 | Copeptin |
| Thirty-one novel biomarkers as predictors for clinically incident diabetes. | Salomaa V, et al | PLoS One. 2010 Apr 9;5(4):e10100. doi: 10.1371/journal.pone.0010100. | PLoS One. 2010 | 20396381 | adiponectin, neopterin MR-proANP NT-proBNP BNP homocysteine Cystatin-C PLA-a D-Dimer Troponin PLGF Vitamin B12 Creatinine PON-1 MR-proADM PLA-M CT-proET1 MPO leptin Copeptin Active-B12 TIMP-1 CRP CK-MB GGT IL-18 insulin ferritin ApoA ApoB |
| Soluble CD36 (sCD36) clusters with markers of insulin resistance, and high sCD36 is associated with increased type 2 diabetes risk. | Handberg A, et al | J Clin Endocrinol Metab. 2010 Apr;95(4):1939-46. doi: 10.1210/jc.2009-2002. Epub 2010 Feb 5. | J Clin Endocrinol Metab. 2010 | 20139232 | sCD36 |
| Fasting plasma glucose levels within the normoglycemic range in childhood as a predictor of prediabetes and type 2 diabetes in adulthood: the Bogalusa Heart Study. | Nguyen QM, et al | Arch Pediatr Adolesc Med. 2010 Feb;164(2):124-8. doi: 10.1001/archpediatrics.2009.268. | Arch Pediatr Adolesc Med. 2010 | 20124140 | Glucose |
| Risk prediction models for the development of diabetes in Mauritian Indians. | Gao WG, et al | Diabet Med. 2009 Oct;26(10):996-1002. doi: 10.1111/j.1464-5491.2009.02810.x. | Diabet Med. 2009 | 19900231 | Glucose, Triglycerides |
| Use of multiple metabolic and genetic markers to improve the prediction of type 2 diabetes: the EPIC-Potsdam Study. | Schulze MB, et al | Diabetes Care. 2009 Nov;32(11):2116-9. doi: 10.2337/dc09-0197. Epub 2009 Aug 31. | Diabetes Care. 2009 | 19720844 | Glucose, HbA1c, Triglycerides, HDL, GGT, ALT,AST |
| Impaired fasting glucose cutoff value of 5.6 mmol/l combined with other cardiovascular risk markers is a better predictor for incident Type 2 diabetes than the 6.1 mmol/l value: Tehran lipid and glucose study. | Harati H, et al | Diabetes Res Clin Pract. 2009 Jul;85(1):90-5. doi: 10.1016/j.diabres.2009.04.006. Epub 2009 May 2. | Diabetes Res Clin Pract. 2009 | 19414206 | Glucose |
| Predicting diabetes: clinical, biological, and genetic approaches: data from the Epidemiological Study on the Insulin Resistance Syndrome (DESIR). | Balkau B, et al | Diabetes Care. 2008 Oct;31(10):2056-61. doi: 10.2337/dc08-0368. Epub 2008 Aug 8. | Diabetes Care. 2008 | 18689695 | Glucose, Triglycerides/ Glucose, GGT |
| Additional contribution of emerging risk factors to the prediction of the risk of type 2 diabetes: evidence from the Western New York Study. | Stranges S, et al | Obesity (Silver Spring). 2008 Jun;16(6):1370-6. doi: 10.1038/oby.2008.59. Epub 2008 Mar 20. | Obesity (Silver Spring). 2008 | 18356828 | WBC, albumin, E-selectin |
| Adipokines and incident type 2 diabetes in an Aboriginal Canadian [corrected] population: the Sandy Lake Health and Diabetes Project. | Ley SH, et al | Diabetes Care. 2008 Jul;31(7):1410-5. doi: 10.2337/dc08-0036. Epub 2008 Mar 13. Erratum in: Diabetes Care. 2008 Aug;31(8):1713. | Diabetes Care. 2008 | 18339973 | CRP, IL-6  serum amyloid A (SAA) |
| Does the new American Diabetes Association definition for impaired fasting glucose improve its ability to predict type 2 diabetes mellitus in Spanish persons? The Asturias Study. | Valdés S, et al | Metabolism. 2008 Mar;57(3):399-403. doi: 10.1016/j.metabol.2007.10.017. | Metabolism. 2008 | 18249214 | Glucose |
| The metabolic syndrome as a predictor of incident diabetes mellitus in Mauritius. | Cameron AJ, et al | Diabet Med. 2007 Dec;24(12):1460-9. Epub 2007 Nov 1. | Diabet Med. 2007 | 17976203 | Glucose, Triglycerides HDL |
| Liver enzymes as a predictor for incident diabetes in a Japanese population: the Hisayama study. | Doi Y, et al | Obesity (Silver Spring). 2007 Jul;15(7):1841-50. | Obesity (Silver Spring). 2007 | 17636103 | GGT, ALT AST insulin CRP |
| Predicting the 20-year diabetes incidence rate. | Dankner R, et al | Diabetes Metab Res Rev. 2007 Oct;23(7):551-8. | Diabetes Metab Res Rev. 2007 | 17315136 | Glucose, HOMA-IR |
| Use of HbA1c in predicting progression to diabetes in French men and women: data from an Epidemiological Study on the Insulin Resistance Syndrome (DESIR). | Droumaguet C, et al | Diabetes Care. 2006 Jul;29(7):1619-25. | Diabetes Care. 2006 | 16801588 | Glucose, HbA1c |
| Urinary albumin excretion and its relation with C-reactive protein and the metabolic syndrome in the prediction of type 2 diabetes. | Brantsma AH, et al | Diabetes Care. 2005 Oct;28(10):2525-30. | Diabetes Care. 2005 | 16186291 | CRP, UAE(24h) |
| Fasting plasma glucose cutoff value for the prediction of future diabetes development: a study of middle-aged Koreans in a health promotion center. | Kim DJ, et al | J Korean Med Sci. 2005 Aug;20(4):562-5. | J Korean Med Sci. 2005 | 16100444 | Glucose |
| The metabolic syndrome as predictor of type 2 diabetes: the San Antonio heart study. | Lorenzo C, et al | Diabetes Care. 2003 Nov;26(11):3153-9. | Diabetes Care. 2003 | 14578254 | Glucose(2h) |
| Serum fructosamine as a marker of 5-year risk of developing diabetes mellitus in patients exhibiting stress hyperglycaemia. | Wahid ST, et al | Diabet Med. 2002 Jul;19(7):543-8. | Diabet Med. 2002 | 12099956 | Glucose, Fructosamine |
| Prediction of diabetes with body mass index, oral glucose tolerance test and islet cell autoantibodies in a regional population. | Rolandsson O, et al | J Intern Med. 2001 Apr;249(4):279-88. | J Intern Med. 2001 | 11298847 | Glucose |
| Prediction of diabetes mellitus (NIDDM). | Ito C, et al | Diabetes Res Clin Pract. 1996 Oct;34 Suppl:S7-11. | Diabetes Res Clin Pract. 1996 | 9015664 | Glucose |
